# Supplementary material for: Genome mining unveils a class of ribosomal peptides with two amino termini
Source: Nat Commun. 2023 Mar 23;14:1624. doi: 10.1038/s41467-023-37287-1 (PMC10036551; doi:10.1038/s41467-023-37287-1)
Supplement: Supplementary file 1 — Supplementary Information [file 41467_2023_37287_MOESM1_ESM.pdf]

Supplementary Information

Genome mining unveils a class of ribosomal peptides with two amino termini

Hengqian Ren[1,2,#], Shravan R. Dommaraju[2,3,#], Chunshuai Huang[2], Haiyang Cui[2], Yuwei Pan[4], Marko Nesic[2,3], Lingyang Zhu[5], David Sarlah[2,3], Douglas A. Mitchell[2,3,6,*], and Huimin Zhao[1,2,3,7,*]

[1]Department of Chemical and Biomolecular Engineering, University of Illinois at Urbana-Champaign, Urbana, IL, USA.

[2]Carl R. Woese Institute for Genomic Biology, University of Illinois at Urbana-Champaign, Urbana, IL, USA.

[3]Department of Chemistry, University of Illinois at Urbana-Champaign, Urbana, IL, USA.

[4]Department of Molecular and Cellular Biology, University of Illinois at Urbana-Champaign, Urbana, IL, USA.

[5]School of Chemical Sciences, NMR Laboratory, University of Illinois at Urbana-Champaign, Urbana, IL, USA.

[6]Department of Microbiology, University of Illinois at Urbana-Champaign, Urbana, IL, USA.

[7]Department of Bioengineering, University of Illinois at Urbana-Champaign, Urbana, IL, USA.

[#]These authors contributed equally to this work: Hengqian Ren, Shravan R. Dommaraju.

* To whom correspondence should be addressed: douglasm@illinois.edu, zhao5@illinois.edu

Table of Contents

Supplementary Methods 1

Supplementary Table 1: Daptide protein co-occurrence table 7

Supplementary Table 2: Comparison of the Dmp chemical shifts observed in 1 (daptide) and hominicin 8

Supplementary Table 3: Comparison of the Ala-23 chemical shifts observed in 1 (daptide) and Ala-20 in hominicin 9

Supplementary Table 4: Indicator strains and corresponding culturing conditions used in the antimicrobial activity screening 10

Supplementary Table 5: Oligonucleotides used for cloning and site-directed mutagenesis 11

Supplementary Table 6: DNA sequences of the *E. coli* codon-optimized *mpa* genes 13

Supplementary Table 7: Naming scheme for daptide biosynthetic genes 14

Supplementary Note: Algorithm for pairing of RRE families with precursor peptide families 15

Supplementary Figure 1: Generalized workflow for bioinformatic analysis of the RRE-Finder exploratory mode dataset 17

Supplementary Figure 2: Daptide precursor peptide sequence logo 18

Supplementary Figure 3: HHpred results for MpaB 19

Supplementary Figure 4: Alternative identified daptide BGCs 20

Supplementary Figure 5: Daptide precursor peptide frequency within a BGC 21

Supplementary Figure 6: Taxonomic comparison of daptide protease lengths 22

Supplementary Figure 7: Phylogenetic tree of aminotransferases 23

Supplementary Figure 8: Annotation of the cloned region from *M. paraoxydans* DSM 15019 24

Supplementary Figure 9: Media and host screen for *mpa* BGC expression 25

Supplementary Figure 10: Purification of daptides 1-3 26

Supplementary Figure 11: HR-MS/MS analysis of 1 27

Supplementary Figure 12: HR-MS/MS analysis of 2 28

Supplementary Figure 13: HR-MS/MS analysis of 3 29

Supplementary Figure 14: NMR spectra of compound 1 in 1,1,3,3,3-hexafluoroisopropanol-d2 (HFIP-*d*2) 47

Supplementary Figure 15: NMR spectra of authentic (*R*)-Dmp and (*S*)-Dmp standards 53

Supplementary Figure 16: Determination of MpaA1 amino acid stereochemistry using LC-MS 54

Supplementary Figure 17: Determination of MpaA1 Dmp stereochemistry 55

Supplementary Figure 18: The *sca* BGC identified from *Streptomyces capuensis* NRRL B-3501 56

Supplementary Figure 19: Heterologous expression and product characterization of *sca*. 57

Supplementary Figure 20: Determination of ScaA1/A2 residue stereochemistry 58

\Supplementary Figure 21: Secondary structure of daptides 1-3 60

Supplementary Figure 22: Comparison of melittin to daptide 1 61

Supplementary Figure 23: Hemolytic activity of daptides 1-3 62

Supplementary Figure 24: Heterologous expression and *mpa* gene omissions in *S. albus* J1074 63

Supplementary Figure 25: Expression of the refactored *mpaABCDE* under various cultivation conditions 64

Supplementary Figure 26: Expression of the refactored *mpaABCDE* with chaperone plasmids 65

Supplementary Figure 27: Expression of the refactored *mpaABCDE* with pGro7 chaperone plasmid under various arabinose concentrations 66

Supplementary Figure 28: Expression of the refactored *mpaABCDE* with pGro7 chaperone plasmid with extended expression time 67
Supplementary Figure 29: MALDI-TOF-MS analysis of MpaA1 leader region variants co-expressed with MpaB and MpaC 68
Supplementary Figure 30: MALDI-TOF-MS analysis of MpaA1 *C*-terminal Thr variants that were co-expressed with MpaB and MpaC 69
Supplementary Figure 31: MALDI-TOF-MS analysis of MpaA1 insertion and deletion variants at the C-terminus that were co-expressed with MpaB and MpaC 70
Supplementary Figure 32: MALDI-TOF-MS analysis of MpaA1 variants in the core peptide region that were co-expressed with MpaB and MpaC 71
Supplementary Figure 33: Assessment of the AlphaFold-Multimer prediction for the MpaB-MpaC-MpaA1 complex 72
Supplementary Figure 34: Electrostatic surface potential of MpaB and MpaC. 73
Supplementary Figure 35: Identification of a hominicin-like BGC from *Staphylococcus pseudintermedius* .. 74
Supplementary Figure 36: The plasmid map of directly cloned *mpa* for heterologous expression in *S. albus* J1074 75
Supplementary Figure 37: Plasmid maps of gene omission for heterologous expression in *S. albus* J1074: pSET-mpaABCDMPT 76
Supplementary Figure 38: Plasmid maps of gene omission for heterologous expression in *S. albus* J1074: pSET-mpaABCDMP 77
Supplementary Figure 39: Plasmid maps of gene omission for heterologous expression in *S. albus* J1074: pSET-mpaABCDM 78
Supplementary Figure 40: Plasmid maps of gene omission for heterologous expression in *S. albus* J1074: pSET-mpaA1BCD 79
Supplementary Figure 41: Plasmid maps of codon-optimized and refactored genes for heterologous expression in *E. coli*: MpaA1A2A3BCDM 80
Supplementary Figure 42: Plasmid maps of codon-optimized and refactored genes for heterologous expression in *E. coli*: MpaABCDM 81
Supplementary Figure 43: Plasmid maps of codon-optimized and refactored genes for heterologous expression in *E. coli*: MpaABCD 82
Supplementary Figure 44: Plasmid maps of codon-optimized and refactored genes for heterologous expression in *E. coli*: MpaABCM 83
Supplementary Figure 45: Plasmid maps of codon-optimized and refactored genes for heterologous expression in *E. coli*: MpaABDM 84
Supplementary Figure 46: Plasmid maps of codon-optimized and refactored genes for heterologous expression in *E. coli*: MpaABC 85
Supplementary Figure 47: Plasmid maps of codon-optimized and refactored genes for heterologous expression in *E. coli*: MpaAB 86
Supplementary Figure 48: Plasmid maps of codon-optimized and refactored genes for heterologous expression in *E. coli*: MpaAC 87

Supplementary Methods

Bacterial strains and reagents. *E. coli* strain NEB10β was used for all cloning experiments. *E. coli* strain WM6026 which was a gift from William Metcalf (University of Illinois at Urbana-Champaign) was used as a conjugation donor for *Streptomyces* heterologous hosts[1]. *S. albus* J1074 and *S. lividans* TK24 were used for heterologous expression of the cloned *mpa* BGC. *E. coli* BL21(DE3) was used for the expression of refactored *mpa* pathway. *M. paraoxydans* DSM 15019 was obtained from the German Collection of Microorganisms and Cell Cultures GmbH (Braunschweig, Germany). *S. albus* J1074 and *S. lividans* TK24 were obtained from the Agricultural Research Service Culture Collection (Peoria, IL). *E. coli* strain NEB10β and BL21(DE3) were purchased from New England Biolabs (Ipswich, MA). Components of cell culture media were purchased from ThermoFisher Scientific (Waltham, MA). Antibiotics were purchased from GoldBio (St. Louis, MO). The hexafluoroisopropanol-d_2 (HFIP; $(CF_3)_2CDOD$; ≥99 atom % D) was purchased from Sigma-Aldrich (St. Louis, MO). The (*R*)-2-(dimethylamino)propionamide and (*S*)-2-(dimethylamino)propionamide were purchased from Alfa Chemistry (Ronkonkoma, NY). All other chemicals were purchased from ThermoFisher Scientific (Waltham, MA). No further purification was performed for any purchased chemicals before use. Restriction enzymes, NEBuffers, T4 DNA polymerase, *E. coli* DNA ligase, dNTPs, NAD^+, NEBuilder HiFi DNA assembly mastermix, and Q5 DNA polymerase were purchased from New England Biolabs (Ipswich, MA). All DNA oligonucleotides were ordered from Integrated DNA Technologies (Coralville, IA). Codon optimized genes were synthesized by Twist Bioscience (South San Francisco, CA).

High-resolution mass spectrometry. Lyophilized, HPLC-purified daptides **1-3** were resuspended in aq. 60% acetonitrile/0.1% acetic acid. Samples were directly infused onto a ThermoFisher Scientific Orbitrap Fusion ESI-MS using an Advion TriVersa Nanomate 100. Calibration was performed with Pierce LTQ Velos ESI Positive Ion Calibration Solution (ThermoFisher). The MS was operated with the following parameters: 100,000 resolution, 2 *m/z* isolation width (MS/MS), 0.4 activation q value (MS/MS), and 30 ms activation time (MS/MS). Fragmentation was performed using collision-induced dissociation (CID) at normalized collision energy. Each peptide was subjected to 30, 70, and 100

normalized collision energy (MS/MS). Data analysis was conducted using the Qualbrowser application of Xcalibur software (version 4.1.31.9, ThermoFisher Scientific).

NMR spectroscopy. The sample was prepared by dissolving ~5 mg of **1** (HPLC-purified and lyophilized) in 500 µL of hexafluoroisopropanol-d2 (HFIP; $(CF_3)_2CDOD$; ≥99 atom % D, Sigma-Aldrich). NMR spectra were recorded on an Agilent VNMRS 750 MHz narrow bore magnet spectrometer equipped with a 5 mm triple resonance (^1H-^{13}C-^{15}N) triaxial gradient probe and pulse-shaping capabilities. Samples were held at 25 °C during acquisition. Standard Varian pulse sequences were used for the following experiments: ^1H, ^{13}C, ^1H-^1H COSY, ^1H-^{13}C HSQC, and ^1H-^{13}C HMBC. Spectra were recorded with VNMRJ 3.2A software. All NMR data were processed using MestReNova 11.0.3. Chemical shifts were referenced internally to the solvent peak (δ_H = 4.4 ppm, δ_c = 68.1, HFIP).

Synthetic procedure for the (*R*)-/(*S*)-Dmp standards

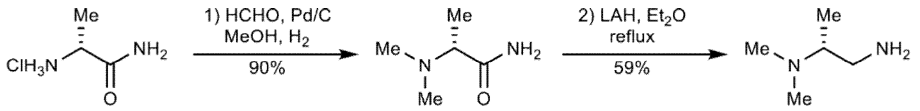

1) (*R*)-2-(dimethylamino)propionamide was prepared according to a known procedure[2]: A mixture of (*R*)-alaninamide hydrochloride (500 mg, 4.01 mmol, 1 equiv.), HCHO (37% aqueous, 0.97 mL) and Pd/C (10% wt, 50 mg) in MeOH (15 mL) was stirred in a flask equipped with a H_2 balloon for 16 h. Upon completion, the mixture was filtered through celite. The filtrate was evaporated, diluted with a mixture of MeOH/DCM (10 mL/10 mL), basified with NH_4OH (28% aqueous, 1 mL), and concentrated. Flash column chromatography (SiO_2) of the residue with DCM/MeOH/28% aqueous NH_4OH (60:10:1) afforded (*R*)-2-(dimethylamino)propionamide (420 mg, 3.62 mmol, 90%).

2) Conditions for amide reduction were reported previously[3]. To a solution of (*R*)-2-(dimethylamino)-propionamide (420 mg, 3.62 mmol) in dry ether (18 mL) was added LAH (412 mg, 10.8 mmol, 3 equiv) at 0 °C portionwise. Upon completed addition, the mixture was heated to reflux and left to stir overnight. The mixture was cooled down to 0 °C, followed by sequential addition of water (0.5 mL), 15% aqueous NaOH (0.5 mL) and water (1.5 mL). After 15 min of stirring, the obtained precipitate was removed by filtration, and the filtrate was dried with $MgSO_4$. Careful evaporation of ether in vacuo (the product is

highly volatile) afforded (*R*)-N_2,N_2-dimethyl-1,2-propanediamine [(*R*)-Dmp] as a colorless oil (218 mg, 2.13 mmol, 59%). Since this compound co-evaporated with diethyl ether, a small amount of ether was always present in the final sample.

$[\alpha]_D^{22}$ = –13.1° (c = 0.35 mg/mL, Et_2O)

^{1}H NMR (500 MHz, DMSO-d_6) δ 3.32 (br, 2H), 2.50 (m, 1H), 2.40 – 2.32 (m, 2H), 2.16 (d, *J* = 2.0 Hz, 1H), 2.12 (s, 6H), 0.82 (d, *J* = 6.3 Hz, 3H).

^{13}C NMR (126 MHz, DMSO-d_6) δ 64.9, 44.9, 40.3, 10.4.

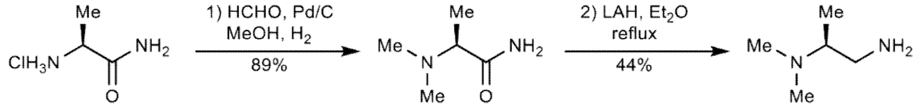

1) (*S*)-2-(dimethylamino)propionamide was prepared according to a known procedure[2]. A mixture of (*S*)-alaninamide hydrochloride (500 mg, 4.01 mmol, 1 equiv), HCHO (37% aqueous, 0.97 mL) and Pd/C (10% wt, 50 mg) in MeOH (15 mL) was stirred in a flask equipped with a H_2 balloon for 16 h. Upon completion, the mixture was filtered through celite. The filtrate was evaporated, diluted with a mixture of MeOH/DCM (10 mL/10 mL), basified with NH_4OH (28% aqueous, 1 mL), and concentrated. Flash column chromatography (SiO_2) of the residue with DCM/MeOH/28% aqueous NH_4OH (60:10:1) afforded (*S*)-2-(dimethylamino)propionamide (416 mg, 3.58 mmol, 89%).

2) Reduction conditions were reported previously[3]. To a solution of (*S*)-2-(dimethylamino)propionamide (416 mg, 3.62 mmol) in dry ether (18 mL) was added LAH (408 mg, 10.7 mmol, 3 equiv) at 0 °C portionwise. Upon completed addition, the mixture was heated to reflux and left to stir overnight. The mixture was cooled down to 0 °C, followed by sequential addition of water (0.5 mL), 15% aqueous NaOH (0.5 mL) and water (1.5 mL). After 15 min of stirring, the obtained precipitate was removed by filtration, and the filtrate was dried with $MgSO_4$. Careful evaporation of ether in vacuo (the product is highly volatile) afforded (*S*)-N_2,N_2-dimethyl-1,2-propanediamine [(*S*)-Dmp] as a colorless oil (160 mg, 1.57 mmol, 44%). Since this compound co-evaporated with diethyl ether, a small amount of ether was always present in the final sample.

$[\alpha]_D^{22}$ = 16.4° (c = 0.40 mg/mL, Et_2O)

^{1}H NMR (500 MHz, DMSO-d_6) δ 3.32 (br, 2H), 2.50 (m, 1H), 2.40 – 2.32 (m, 2H), 2.16 (d, J = 2.0 Hz, 1H), 2.12 (s, 6H), 0.82 (d, J = 6.3 Hz, 3H).

^{13}C NMR (126 MHz, DMSO-d_6) δ 64.9, 44.9, 40.3, 10.4.

Characterization of stereochemistry by Marfey's assays. Purified **1** (400 μg) was dried in vacuo in a Schlenk flask. To the dried peptide, 300 μL of a 1:1 mixture 12 M DCl (35% *w/v*, Aldrich) and D_2O (Cambridge Isotope Laboratory) containing 3% phenol (*w/v*) was added. The solution was equilibrated under nitrogen gas and subjected to one freeze-pump-thaw cycle. The sample was then frozen, subjected to vacuum, and sealed. Once equilibrated back to room temperature, the sample was heated to 95 °C for 2 h under reduced pressure. For the ScaA1/A2 peptide mixture, the same hydrolysis procedure was conducted in 1.6 mL of the 1:1 mixture 12 M DCl and D_2O and heated at 95 °C for 16 hr. After cooling back to room temperature, insoluble debris was removed by centrifugation for 5 min, and supernatant was dried using a Speedvac concentrator (Savant ISS110).

The hydrolysate of **1** was dissolved in 150 μL 1M aq. $NaHCO_3$ and 150 μL acetone containing 4 mg/mL 1-fluoro-2-4-dinitrophenyl-5-L-alanine amide (L-FDAA, Thermo Scientific) was added. For the ScaA1/A2 sample, the hydrolysate was dissolved in 150 μL 1M aq. NaHCO3 and 150 μL acetone containing 12 mg/mL L-FDAA. Contents were mixed, and the reaction vessel was heated to 60 °C for 2 h without shaking using an Eppendorf ThermoMixer C. The reaction mixture was then quenched using 40 μL 6 M HCl dropwise. Sample was dried using a Speedvac concentrator (Savant ISS110). Amino acid standards were prepared by drying down 150 μL L-amino acid solution (0.5 μmol/mL), followed by dissolution in 1 M aq. $NaHCO_3$ and mixing with acetone containing 4 mg/mL L-FDAA as above. D-amino standards were generated by reacting the L-amino acid solution with 1-fluoro-2-4-dinitrophenyl-5-D-alanine amide (D-FDAA, Toronto Research Chemicals). These reaction products are enantiomers to the hypothetical D-amino acid and L-FDAA coupled products, where both are chromatographically identical using an achiral stationary phase. Standards for (*R*)-N_2,N_2-dimethyl-1,2-propanediamine and (*S*)-N_2,N_2-dimethyl-1,2-propanediamine were prepared by adding 1 μL of

synthesized standard to 149 µL 1M aq. $NaHCO_3$ and mixed with acetone containing 4 mg/mL L-FDAA as above.

Derivatized amino acid standards, Dmp standards, and MpaA1 hydrolysate were reconstituted in 300 µL 15% aq. MeCN + 0.1% formic acid (LC-MS grade). Samples were sonicated for 5 minutes in a sonication bath and insoluble material was removed by filtration with a 0.2 µm centrifugal filter (Pall Corporation) or by centrifugation for 5 min. Amino acid stereochemistry was determined using a Shimadzu LC-MS 2020 with a quadrupole detector and a Macherey-Nagel Nucleodur C18 column (250×4.6 mm, 5 µm particle size, 100 Å pore size). Samples were analyzed in batch using the following conditions: solvent A 0.1% aq. formic acid, solvent B MeCN + 0.1% formic acid, 1 mL/min flow rate, and the following method: 15% B for 7 min, 15-50% B over 45 min, hold at 50% B for 5 min followed by wash and re-equilibration in starting conditions. Analyte (20 µL) was injected and the $[M+H]^+$ ions of the amino acid-Marfey's reagent adducts were selective monitored as follows (*m/z*): Ser, 358; Trp, 457; Asp, 386; Phe, 418; Tyr, 434; Glu (amide of Gln hydrolyzed), 400; Val, 370; Ile/Leu, 384; Ala, 342; FDAA(bis)-Tyr, 686. For the ScaA1/A2 sample, the following ions were selectively monitored (*m/z*): Ala, 342; Phe, 418; Ile/Leu, 384; Thr, 372; Val, 370; FDAA(bis)-Tyr, 686. To clarify Asp and Ser stereochemistry for **1**, amino acid-Marfey's reagent adduct standards were mixed 3:1 with MpaA1 hydrolysate, and 20 µL of the resulting mixtures were injected on the LC-MS as above. Dmp stereochemistry was determined by co-injection of derivatized MpaA1 hydrolysate with derivatized Dmp standards on a Thermo Vanquish Duo binary pump UHPLC system and a Thermo Scientific Accucore C18 column (150×4.6 mm, 2.6 µm particle size, 80 Å pore size). Samples were analyzed using the following conditions: solvent A 0.1% aq. formic acid, solvent B MeCN + 0.1% formic acid, 2 mL/min flow rate and the following method: 15% B for 0.5 min and 15-22% B over 7 min, followed by wash and re-equilibration in starting conditions. To obtain optimal peak resolution, derivatized Dmp standards and MpaA1 hydrolysate were diluted >100 fold before injection of 20 µL of sample in each run.

Circular dichroism spectrometry. CD spectra were acquired using a JASCO spectropolarimeter J-715 with Peltier temperature control. Stock solutions of **1**, **2**, and **3** (10 mM in HFIP) were diluted by

water to 50 μM [final concentration of HFIP at 0.5% (*v/v*)] immediately prior to data acquisition. Spectra were recorded from 260 to 200 nm at a scan rate of 200 nm/min at 0.1 nm resolution at 10 °C. The data shown are the average of three acquisitions.

Supplementary Table 1: Daptide protein co-occurrence table. RODEO[4] annotation used the Pfam[5], TIGRFAM[6], and custom RiPP recognition element (RRE) profile hidden Markov models (pHMMs)[7]. The top three hits for each annotated CDS were compiled and counted for frequency. The top 32 highest occurring pHMM hits in daptide BGCs are listed with custom pHMM models for daptide detection shown in red.

Count	% Co-occurrence	pHMM ID	pHMM description
**486**	101%	PF00202	Aminotransferase class-III
**486**	101%	TIGR00707	argD: transaminase, acetylornithine/succinylornithine family
**484**	100%	TIGR00508	bioA: adenosylmethionine-8-amino-7-oxononanoate transaminase
**456**	94%	PF13649	Methyltransferase domain
**452**	94%	TIGR01188	drrA: daunorubicin resistance ABC transporter, ATP-binding protein
**381**	79%	PF08241	Methyltransferase domain
**377**	78%	TIGR03740	galliderm_ABC: lantibiotic protection ABC transporter, ATP-binding subunit
**375**	78%	TIGR03522	GldA_ABC_ATP: gliding motility-associated ABC transporter ATP-binding subunit GldA
**319**	66%	**Actino_DapB_RRE**	C-terminal RRE domain from Actinomycetota DapB proteins
**307**	64%	PF00465	Iron-containing alcohol dehydrogenase
**302**	63%	TIGR03405	Phn_Fe-ADH: phosphonate metabolism-associated iron-containing alcohol dehydrogenase
**289**	60%	**Actino_DapP_RRE**	N-terminal RRE domain from Actinomycetota DapP proteins
**192**	40%	PF13561	Enoyl-(Acyl carrier protein) reductase
**185**	38%	TIGR02638	lactal_redase: lactaldehyde reductase
**183**	38%	PF12698	ABC-2 family transporter protein
**178**	37%	PF13489	Methyltransferase domain
**176**	36%	**Bacill_DapB_RRE**	C-terminal RRE domain from Bacillota DapB proteins
**163**	34%	TIGR01830	3oxo_ACP_reduc: 3-oxoacyl-[acyl-carrier-protein] reductase
**154**	32%	PF00528	Binding-protein-dependent transport system inner membrane component
**145**	30%	PF02163	Peptidase family M50
**142**	29%	PF00583	Acetyltransferase (GNAT) family
**142**	29%	**Bacill_DapP_RRE**	N-terminal RRE domain from Bacillota DapP proteins
**139**	29%	PF02624	YcaO cyclodehydratase, ATP-ad Mg2+-binding
**134**	28%	TIGR03604	TOMM_cyclo_SagD: thiazole/oxazole-forming peptide maturase, SagD family component
**124**	26%	PF00069	Protein kinase domain
**123**	26%	PF00296	Luciferase-like monooxygenase
**120**	25%	TIGR03564	F420_MSMEG_4879: F420-dependent oxidoreductase, MSMEG_4879 family
**120**	25%	TIGR03841	F420_Rv3093c: probable F420-dependent oxidoreductase, Rv3093c family
**120**	25%	PF08242	Methyltransferase domain
**105**	22%	TIGR01575	rimI: ribosomal-protein-alanine acetyltransferase
**104**	22%	PF12730	ABC-2 family transporter protein
**96**	20%	PF05147	Lanthionine synthetase C-like protein

Supplementary Table 2: Comparison of the Dmp chemical shifts observed in 1 (daptide) and hominicin[8]. Data was obtained in 1,1,1,3,3,3-hexafluoroisopropanol-d_2 (HFIP-d_2) for **1** and H_2O + D_2O for hominicin, respectively. [§]Data not available. *Signals assigned to *N*-methyl groups are not distinguished in the original paper. Dmp: (*S*)-N_2,N_2-dimethyl-1,2-propanediamine.

No.	**1 (daptide)**		**Hominicin**	
	δ_C type	δ_H multi	δ_C type	δ_H multi
1	39.1 CH_2	3.91 overlapped	39.9 CH_2	3.46 d (6.3)
		3.38 d (15.2)		3.41 m
2	61.7 CH	3.75 m	60.9 CH	3.48 m
3	6.9 CH_3	1.34 brs	10.9 CH_3	1.23 d (6.7)
4	34.1 CH_3	2.80 s	38.2 CH_3	2.75* s
5	41.6 CH_3	2.97 s	41.0 CH_3	2.80* s
NH		N/A[§]		8.24 t (5.9)

Supplementary Table 3: Comparison of the Ala-23 chemical shifts observed in 1 (daptide) and Ala-20 in hominicin[8]. Data was obtained in 1,1,1,3,3,3-hexafluoroisopropanol-d_2 (HFIP-d_2) for **1** and H_2O + D_2O for hominicin, respectively. [§]Data not available. [*]Carbonyl carbon of Ala-22 in **1** and Ile-19 in hominicin.

No.	**1 (daptide)**		**Hominicin**	
	δ_C type	δ_H multi	δ_C type	δ_H multi
1’	51.0 CH	4.22 overlapped	50.0 CH	4.18 m
2’	13.7 CH_3	1.57 d (6.9)	16.4 CH_3	1.29 d (7.4)
NH		N/A[§]		8.32 d (5.8)
CO	175.8 C		175.5 C	
CO[*]	176.4 C		173.3 C	

Supplementary Table 4: Indicator strains and corresponding culturing conditions used in the antimicrobial activity screening. Recipes for mediums used in the bioassay are as the following: lysogeny broth (LB): 10 g/L tryptone, 10 g/L NaCl, and 5 g/L yeast extract; brain heart infusion broth (BHI): 5 g/L beef heart (infusion from 250 g), 12.5 g/L calf brains (infusion from 200 g), 2.5 g/L Na_2HPO_4, 2 g/L glucose, 10 g/L peptone, and 5 g/L NaCl; international Streptomyces project-2 medium (ISP2): 10 g/L malt extract, 4 g/L yeast extract, and 4 g/L glucose; ATCC medium 172 (ATCC 172): 10 g/L glucose, 20 g/L soluble starch, 5 g/L yeast extract, 5 g/L N-Z Amine Type A (Sigma C0626), and 1 g/L $CaCO_3$; yeast extract–peptone–dextrose medium (YPD): 10 g/L yeast extract, 20 g/L peptone, and 20 g/L glucose.

Phylum	Strain	Medium	Temperature (°C)
Bacillota	*Bacillus cereus* TZ417	LB	30
	Bacillus subtilis ATCC 6633	LB	30
	Lactococcus lactis CNRZ 481	LB	30
	Staphylococcus epidermidis 15X154	LB	37
	Staphylococcus aureus USA300	BHI	37
	Streptococcus mutans ATCC 25175	BHI	37
Actinomycetota	*Micrococcus luteus* ATCC 4698	LB	30
	Streptomyces albus J1074	ISP2	30
	Streptomyces lividans TK24	ISP2	30
	Streptomyces coelicolor M145	ISP2	30
	Microbacterium aurum B-24210	ATCC 172	30
	Microbacterium esteraromaticum B-24213	ATCC 172	30
	Microbacterium hominis B-24220	ATCC 172	30
	Microbacterium ketosireducens B-24221	ATCC 172	30
	Microbacterium kitamiense B-24226	ATCC 172	30
Pseudomonadota	*Escherichia coli* DH5a	LB	37
	Enterobacter cloacae	LB	30
	Pseudomonas fluorescens Pf-5	LB	30
	Pseudomonas putida mt-2	LB	30
Ascomycota	*Saccharomyces cerevisiae* YSG50	YPD	30
	Aspergillus terreus	YPD	30

Supplementary Table 5: Oligonucleotides used for cloning and site-directed mutagenesis. The primers are named for the annealing region and whether the primer was for the forward (F) or reverse (R) direction.

Name	Nucleotide Sequence (5' to 3' direction)
pBE44 F	ttaccaatgcttaatcagtgaggcacc
pBE45 R	atctttatagtcctgtcgggtttcg
pBE44-DSM15019-ami R	accgtgttcgtcttcccggatctcaacaccggcaacaacaacttatatcgtatggggctg
pBE45-DSM15019-ami F	cactcccccgccttcccaccgttctccacacccttctctgacgctcagtggaacgaaaac
DSM15019-g1 F	aattaatacgactcactatagggaatttctactgttgtagatccggatctcaacaccggc
DSM15019-g1 R	gccggtgttgagatccggatctacaacagtagaaattccctatagtgagtcgtattaatt
DSM15019-g2 F	aattaatacgactcactatagggaatttctactgttgtagatccaccgttctccacaccc
DSM15019-g2 R	gggtgtggagaacggtggatctacaacagtagaaattccctatagtgagtcgtattaatt
pBE44-B3501-ami R	ggtcacctgcgcttcttcgaacatgtccgagccggtacaacaacttatatcgtatggggctgacttc
pBE45-B3501-ami F	cgcacagaagctttgaaggtcgaaagggatagggatatcgacgctcagtggaacgaaaac
B3501-g1 F	aattaatacgactcactatagggaatttctactgttgtagatttcgaacatgtccgagcc
B3501-g1 R	ggctcggacatgttcgaaatctacaacagtagaaattccctatagtgagtcgtattaatt
B3501-g2 F	aattaatacgactcactatagggaatttctactgttgtagataaggtcgaaagggatagg
B3501-g2 R	cctatccctttcgaccttatctacaacagtagaaattccctatagtgagtcgtattaatt
pSET (XbaI)-mpaB F	gacggccagtgccaagcttgggctgcaggtcgactctagacgccgcccgaaacgagagaa
mpaP R	gcgacgccgatcgggtcgag
mpaM F	tcctcacgacctcccgcgcc
Yeast-mpaT3 R	ttatagcacgtgatgaaaaggacccaggtggcacttttcgccaggaatcctacgtcgcct
mpaT3-Yeast F	acgtgtcgtagcgcttcaggaggcgacgtaggattcctggcgaaaagtgccacctgggtc
pSET (EcoRI)-Yeast R	cacacaggaaacagctatgacatgattacgaattcgcatcacagtatcgtgatgacagtg
Yeast-mpaP R	tagcacgtgatgaaaaggacccaggtggcacttttcgtcatcctctctcctcctcgctcg
mpaP-Yeast F	cccgctggcagctctcacgagcgaggaggagagaggatgacgaaaagtgccacctgggtc
Yeast-mpaM R	ttatagcacgtgatgaaaaggacccaggtggcacttttcgtcatcgccggccccttccat
mpaM-Yeast F	cacgacctcccgcgccggggatggaaggggccggcgatgacgaaaagtgccacctgggtc
Yeast-mpaA1 R	tatagcacgtgatgaaaaggacccaggtggcacttttcggcgtccctccttcctttctcc
mpaA1-Yeast F	cgtccggcagcacccgatcggagaaaggaaggagggacgccgaaaagtgccacctgggtc
BbsI-mpaA1 F	acgtgaagacaaaatgatgaatgaatccacagaaatgtcgatccg
BbsI-mpaA1 R	acgtgaagacaaaccgtcaggtcgctgccgctgc
mpaA1 E13A R	ttccatcgggtccagcgcttgaaagcggatcga
mpaA1 E13A F	tcgatccgctttcaagcgctggacccgatggaa
mpaA1 L14A R	ggcttccatcgggtccgcctcttgaaagcggat
mpaA1 L14A F	atccgctttcaagaggcggacccgatggaagcc
mpaA1 D15A R	gggggcttccatcggcgccagctcttgaaagcg
mpaA1 D15A F	cgctttcaagagctggcgccgatggaagccccc
mpaA1 P16A R	actgggggcttccatcgcgtccagctcttgaaa
mpaA1 P16A F	tttcaagagctggacgcgatggaagcccccagt
mpaA1 M17A R	ccaactgggggcttccgccgggtccagctcttg
mpaA1 M17A F	caagagctggacccggcggaagcccccagttgg
mpaA1 E18A R	atcccaactgggggccgccatcgggtccagctc
mpaA1 E18A F	gagctggacccgatggcggcccccagttgggat

mpaA1 P20A R	aaaggaatcccaactcgcggcttccatcgggtc
mpaA1 P20A F	gacccgatggaagccgcgagttgggattccttt
BbsI-mpaA1 Ser R	acgtgaagacaaaccgtcagctcgctgccgctgcgccaatac
BbsI-mpaA1 Cys R	acgtgaagacaaaccgtcagcacgctgccgctgcgccaatac
BbsI-mpaA1 Asp R	acgtgaagacaaaccgtcaatccgctgccgctgcgccaatac
BbsI-mpaA1 Asn R	acgtgaagacaaaccgtcagttcgctgccgctgcgccaatac
BbsI-mpaA1 Glu R	acgtgaagacaaaccgtcattccgctgccgctgcgccaatac
BbsI-mpaA1 Gln R	acgtgaagacaaaccgtcactgcgctgccgctgcgccaatac
BbsI-mpaA1 Gly R	acgtgaagacaaaccgtcagcccgctgccgctgcgccaatac
BbsI-mpaA1 Ala R	acgtgaagacaaaccgtcacgccgctgccgctgcgccaatac
BbsI-mpaA1 Phe R	acgtgaagacaaaccgtcaaaacgctgccgctgcgccaatac
BbsI-mpaA1 Lys R	acgtgaagacaaaccgtcatttcgctgccgctgcgccaatac
BbsI-mpaA1 Arg R	acgtgaagacaaaccgtcaacgcgctgccgctgcgccaatac
BbsI-mpaA1 Val R	acgtgaagacaaaccgtcacaccgctgccgctgcgccaatac
BbsI-mpaA1 +44A R	acgtgaagacaaaccgtcaggtcgccgctgccgctgcgccaatac
BbsI-mpaA1 -43A R	acgtgaagacaaaccgtcaggttgccgctgcgccaataccga
BbsI-mpaA1 +45A R	acgtgaagacaaaccgtcacgcggtcgctgccgctgcgccaa
BbsI-mpaA1 G39P R	acgtgaagacaaaccgtcaggtcgctgccgctgccggaataccgatc
BbsI-mpaA1 G37P R	acgtgaagacaaaccgtcaggtcgctgccgctgcgccaatcgggatcaggaca
BbsI-mpaA1 V34P R	acgtgaagacaaaccgtcaggtcgctgccgctgcgccaataccgatcagcggaagcgctc
BbsI-mpaA1 G31P R	acgtgaagacaaaccgtcaggtcgctgccgctgcgccaataccgatcaggacaagcgccgggatgac acct
BbsI-mpaA1 G28P R	acgtgaagacaaaccgtcaggtcgctgccgctgcgccaataccgatcaggacaagcgctccgatgac cggttgataaaag

Supplementary Table 6: DNA sequences of the *E. coli* codon-optimized *mpa* genes.

Gene	Nucleotide Sequence (5’ to 3’ direction)
mpaA1	atgaatgaatccacagaaatgtcgatccgctttcaagagctggacccgatggaagcccccagttgggattccttttatc<br>aaggtgtcatcggagcgcttgtcctgatcggtattggcgcagcggcagcgacctga
mpaA2	atgatgaatgccgcgctgcctgccttagagtttacggagctggaagctatggatgcgccgggcgatgcagatgattata<br>tccgtggcttcgctgtgggcgttatcatcgggattctcgccctgacctaa
mpaA3	atgcaatcaacatcccttgaatttctggaattagaacagatcgacactccattggaatggtgggaacatgcgagttaca<br>ttattgcaattattggtggtgcagctgccattgcgacgtga
mpaB	atgggaacctcagctacgaccgcacgccaagacgacggctcaaccggcgaagatccgttattaggccctcatgtccttc<br>gcgcatgggtaacgggcggccacgcggcccgtggtcgcgttttcctgcgtgaggcagaaacgggtgcggaagcgttagc<br>tgcagcggctgatccggaagatgtcattctggagccagccggttctgcgcctcgtccaggagcagcccgtgtcgttggg<br>tatggcggacgcctggccgatttgggtgatgaactctttctcggcgaacgcggagtggagctgcaagactatatcgccg<br>ccagtttcgttcagatcgttggtcccacggccatgcgtttctttgatgaggcttcctggcgtgccttcctcgatgatgc<br>cgatctcgcacgtggcaccggcgtgtttgccgcggcaatgctggacccacgtgtcttattggccgaccgctcagcactg<br>gcgcgcccgcaggaagtggagacgccacgtgccgtacgcatcgacggggacggtggtgtgcacttaggcctgcagggcg<br>aggcaattggtcatgttgacgatcttccagacgccctgggttgggcgttacctcgtgcttcaatgcttggtggtgttcc<br>tggctcggccgatctcgttgccgaacttgatacgcgtccatggttacgccgctacctcgatgctgcggaccttcgtaaa<br>atgttacgcctgccgaatggcaccgcacgtattgcaggctacggttgggcattactggatgacgatctggcggaagcgg<br>aagcccttgcggatgaccctttcttttagataccgccgaaggttttctgttggcggatgtgcgtacccttcgccgtca<br>cttactttcgcctttaacagccgcggtggtggatgccatccagactagtagcacgcgtggtcgtgctgcggcacgcgtg<br>gctcgcgcctgtggcgtgccggatgcgcacgcccgccatttgtgtttggaagcgttagcagctctgggcgttcacttag<br>gggcacgcaccgaggacgcatctggtgatggggcacgttga
mpaC	atgactgtacccttcacagcccgcgttccgaccttatggcacggcagtcgcgcacgcgagcgcatggtaggtctgactg<br>cgggccgtgataccctggtgctggctgacgcggcggtagcgcatcccttgcctactcctcaatcgggccgcactatgac<br>cgtcgacgccgccgcggtcgacgagttgggaattcttctgatcgcggaagaactggtccgtcgtccgcctgaagtgatc<br>gtggcggtcggtggcggctcagttctggatgcctctaagattgccgcgctggctctctctcctgggcgcttgcttgatt<br>tcgcacttcgtcacgcagccacgtctgctcttaccatcttaccggacgctcccccgccagtggacattgtggcgatgcc<br>aacgacccttggaacctcttcggaaaccaacagcgtggccatccttcgtaatcgttgtggcgctcgtctgcttgtggga<br>cgtcccttacgtccgcgccacgcagtcttggacccggaccagctggccactttgacccctgcagcggttcgtgagggcg<br>ctcttgaggcgtttctccgcgtcgccggagcgagtacctcaccacgtacagcccgcgccgaccgtgatgcgcgtatctt<br>aggccgcgcgttgctggaagcggctgcacgtgatgtaatgtctacgaccggtcgtctccgtcttgctcgccttagcgcg<br>gccactcagcgcactgccgctctccgtggccctgacccgtattctgcacgccactggtacttggccaatgaggtagcct<br>tccacctgggagtgcgcaaaatgacggcaactgcggcagtgattgcagccgtatggcaacgcatcggtgcgggcgaccc<br>gcgctggggtgaccgtgcgagcttggacgcattttgggctggagttgcagcaggaacggcgttacctcgtgatcctgca<br>gctggaatcgcggtagtggtagatcagggcgggttacccagcccaccacgccccagcgcgcgtgagattgatcgtattg<br>cggacgcagtcgaacgtgcgtggggagatcatcgccctatgttgcgcggcattcgcgccgctgatgtacgtgcagtttt<br>gcacgactcccgctggagcccttccccggtaggcgatcgtcgtcctcgtgtggcagagcgtcgttga
mpaD	atgagcgttccgcatgccctttggacctctatgcttccagcagacaccgcatttccgcccgaccgcgtcgccatcgggg<br>ccgcaggccaccgtttgttctacgcagacgggtctgatcgtttatgcgctacaagtgggttgtggaacgtaccgttggg<br>atttgggaatccagcggtcaccgaggctgtctcacgcgcgactcgtgacgcgtcctacctgagcttattccgtgctccc<br>caccgttacgcagaggatgcggcggatgcgttaattgcgctcgcaggaagcgaccgttaccgccgcgtcatctttagca<br>ctagtggcggcgccgcaaatgatgccgctatgaaactcgcccgtcaatattgggcacaacaaggtgctggtagccgttc<br>tttagtagttggtttacgcggtagctatcatggtacgatgtatggctcccacgttctttcgggcgacgatctgctgcag<br>tcggtgtatggcgtcgaccgtcgtacggtacgccacgtaagtcacacggatgaaggggctgagctggaagcgttgctgg<br>agcgcgaaggttcccgtgtcgccagtgtcgttgtcgagccggtactgggttcaggtgcccatgcgctttctgctgcatt<br>cgtgcgtcgcttgctcgagttgcgcgaacgccatgggtttctgttagttgcggacgaggtagccaccgggttcggtcgt<br>acgggcgcgatgttcgccactgaaggttgggcagccgctccagatgtccttcttgtatcaaaggcgctcacgaatggag<br>ctatgggcgcagcagcgctcttggtgggtgatcgcattgcaaccgcctttgcgcgtggcggctggacatttgttcacgg<br>tgagacacaagccgggacgcctgcctgtgcagcggctgtccttgcggttatcgacgagcttcgtcgtattgacgtcgag<br>gcgaccactcaagcgctggctagcacgttacatcgtttggcccgcacgtggcaagaggacggcatcgtgaccgatgtta<br>gcggaagtggttgcttcgtgggtgtaggattacgccgtcctgacggttcagctctgagcggaaccgacgtgctggaagt<br>ggttagtgccattgcggattccggcgttgttgtacatcctggcccgtcatcatttcagttaatccctggctacggtttt<br>acacctgcagaggtagcggaaactgaccgcgcggtgcgtggcggcttggcacgtgcccgtgaggcggtggcatga
mpaM	atgacctcgttgattaccgagactgtcgccgcccgtctggaccttgtgggtgctaccgcccgtgcgcaagacttgtatg<br>caggcgcgggtacagacttttatgatcgcctcgtaggtcccgatcgtgccgaaattcgtgaggtactgggtttagccca<br>caccgcacccggtccggtactggacctcgcagcgggttctggccgtttgacaattcctcttgcgcgtagtggccaccgc<br>gtgacagccgtggaccttagtgcggatatgttagcccgtttgcgtggcgcagtcccgcatggcgccaccgtagaatgcg<br>ttgtggctgatatgcgtgacttagcacttggcggacacttcggcctggtagttctcggagcaacgtcgatcaccctgct<br>cgaccgcgaagatcgcgcacgcctctacgccggtgtgcgtcgtcatcttgcatcagcaggtgtctttgcattgaccatt<br>gcggacggtgtcagtgcagacacgcttgcgatcccaacggaccgcgaaattacggttcctggttccgcaggtgatgagc<br>cttacttattcgcacaacaaatcgaggatgatggggctgcgcgtcttgtaaactgggtccgcctggctgacatcgcccc<br>acgtgcagaagttccggtgttaacatcacgcttgcacgtcttaaccccagaggtcctggctggcgagttagtagcagcc<br>gggtttacagcacccgagcgctcgccggttcgcactccggcaggtgttgagattttgttgttaacgacctcgcgcgccg<br>gggacggacgcggtcgtcgctaa

Supplementary Table 7: Naming scheme for daptide biosynthetic genes. Custom pHMM models for daptide detection are shown in red.

Gene Name	Function	pHMM annotations for identification
**DapA**	Precursor peptide	N/A
**DapB**	DUF-RRE fusion protein	Actino_DapB_RRE, Bacill_DapB_RRE
**DapC**	Oxidative decarboxylase (alcohol dehydrogenase)	PF00465, PF13561
**DapD**	Aminotransferase	PF00202
**DapM**	Methyltransferase	PF13649, PF08241, PF13489, PF08242, *etc*.
**DapP**	Intramembrane protease	PF02163, Actino_DapP_RRE, Bacill_DapP_RRE
**DapT**	ABC transporter	TIGR01188, TIGR03740, PF00528, *etc*.

Supplementary Note: Algorithm for pairing of RRE families with precursor peptide families.

```
import argparse
import sys
import os
import re

def __main__():

    input_file = open(RRE_ALL_BY_ALL_BLAST_FILE, 'r')
    input_precursor_file = open(PRECURSOR_ALL_BY_ALL_BLAST_FILE, 'r')
    enzyme_edge_data = input_file.read().split("\n")
    precursor_edge_data = input_precursor_file.read().split("\n")
    input_file.close()
    input_precursor_file.close()
    cutoff = 50
    print("Starting enzyme edge filtering...")


    enzyme_edges_withAS = []
    for line in enzyme_edge_data:
        values = line.split()
        try:
            enzyme_edges_withAS.append([values[0], values[1], float(values[11])])
        except:
            pass

    print("Starting ID mapping...")

    precursor_edges_withids = []
    for line in precursor_edge_data:
        try:
            node1, node2, BS = re.split("\t", line)
            if float(BS) > 10:
                precursor_edges_withids.append([node1, node2])
        except:
            pass

    open("temp.tsv", 'w').close()

    enzyme_cluster_generate(output_file_name=”temp.tsv", enzyme_edges_withAS=enzyme_edges_withAS,
cutoff=cutoff, precursor_edges_withids=precursor_edges_withids)


def enzyme_cluster_generate(output_file_name, enzyme_edges_withAS=[], cutoff=1000,
precursor_edges_withids=[]):

    print("Starting enzyme graph generation... cutoff = ", cutoff)

    enzyme_edges = []
    for edge in enzyme_edges_withAS:
        if edge[2] > cutoff and edge[0] < edge[1]:
            enzyme_edges.append([edge[0], edge[1]])
    enzyme_clusters = sorted(connected_components(enzyme_edges), key=len, reverse=True)

    print("Starting precursor graph generation...")


    for se in enzyme_clusters:
        print("Cutoff: ", cutoff, "Enzyme cluster size: ", len(se))
        if len(se) < 30:
            print("Too small:", len(se))
            break
        precursor_edges = []
        new_precursor_edges_withids = []
        for edge in precursor_edges_withids:
            if edge[0].split("_")[0] in se and edge[1].split("_")[0] in se:
                precursor_edges.append([edge[0], edge[1]])
            new_precursor_edges_withids.append(edge)
        edge_count = 0
        for enzyme_edge in enzyme_edges:
            if enzyme_edge[0] in se and enzyme_edge[1] in se:
                edge_count += 1
        enzyme_connectivity = (((2 * edge_count) / float(len(se))) / float((len(se)-1)))
        print("Enzyme connectivity: " + str(enzyme_connectivity))

        precursor_clusters = sorted(connected_components(precursor_edges), key=len, reverse=True)
        try:
            x = precursor_clusters[0]
        except:
            return
        if len(x) < 5:
            return
        print("Precursor cluster size: ", len(x))
        cluster_found=False

        if len(x) > 0.1 * len(se) and enzyme_connectivity>0.2:
```

```
            print("Precursor cluster size: " + str(len(x)) + "    Enzyme cluster size: " + str(len(se)) + "
BS cutoff: " + str(cutoff))
            cluster_found=True
            precursor_edge_count = 0
            for z in precursor_edges:
                if z[0] in x and z[1] in x and z[0] != z[1]:
                    precursor_edge_count+=1
            precursor_connectivity = precursor_edge_count / len(x) / (len(x)-1)
            print("Precursor connectivity: " + str(precursor_connectivity))

        new_enzyme_edges=[]
        real_node_list = []
        for edge in enzyme_edges_withAS:
            if edge[0] in se and edge[1] in se:
                new_enzyme_edges.append(edge)
                if edge[0] not in real_node_list:
                    real_node_list.append(edge[0])
                if edge[1] not in real_node_list:
                    real_node_list.append(edge[1])
        if cluster_found==False:
            enzyme_cluster_generate(output_file_name=output_file_name,
enzyme_edges_withAS=new_enzyme_edges, cutoff=cutoff+10,
precursor_edges_withids=new_precursor_edges_withids)
        else:
            precursor_sequences = open(PRECURSOR_FASTA_FILE, 'r').read().split(">")
            output_file = open(output_file_name, 'a')
            output_file.write(str(cutoff) + "\t" + ",".join(se) + "\t" + str(len(se)) + "\t" +
str(enzyme_connectivity)+"\t")
            for precursor in precursor_sequences:
                header = precursor.split("\n")[0]
                if (header) in x:
                    output_file.write(">" + header + " " + "".join(precursor.split("\n")[1:]) + ",")
                continue
            output_file.write("\t" + str(len(x)) +"\t"+str(precursor_connectivity) + "\t" +
",".join(real_node_list) + "\t" + str(len(real_node_list)) + "\n")
            output_file.close()

def connected_components(pairs):
    components = []
    for a, b in pairs:
        a_num = -1
        b_num = -2
        for i, component in enumerate(components):
            if a in component:
                a_num = i
            if b in component:
                b_num = i
            if a_num != -1 and b_num != -2:
                break
        if a_num == -1:
            if b_num == -2:
                components.append([a,b])
                #print(a,b)
            else:
                components[b_num].append(a)
                #print(a,b,components[b_num])
        elif b_num == -2:
            components[a_num].append(b)
            #print(a,b,components[a_num])
        elif a_num != b_num:
            components[a_num].extend(components[b_num])
            components[b_num] = []
    return components

if __name__=="__main__":
    __main__()
```

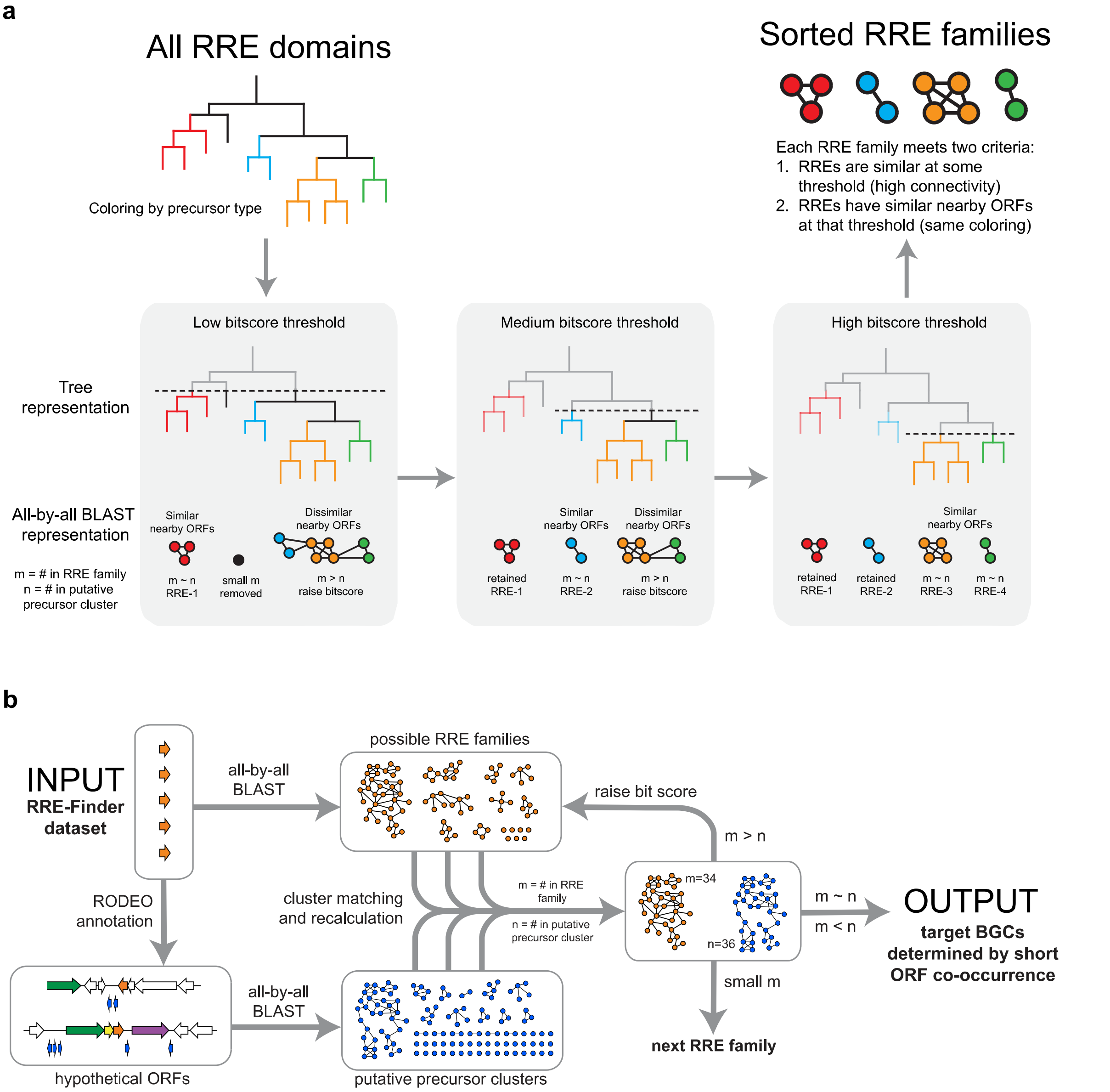

Supplementary Figure 1: Generalized workflow for bioinformatic analysis of the RRE-Finder exploratory mode dataset. The comprehensive description for this workflow is in the Methods section, and code used for implementation is on Github (https://github.com/the-mitchell-lab/rodeo2) and in the **Supplementary Note**. Phylogenetic trees, sequence similarity networks, and biosynthetic gene clusters depicted in this figure are stylized. (**a**) Logical basis for finding new RRE-dependent RiPP pathways. Analysis of RRE domain clusters is performed iteratively with increasing bitscore thresholds. Examination of each cluster for high similarity nearby open-reading frames (ORFs) allows categorization of RRE families based on sequence similarity of the RRE domains and co-occurrence with high-likelihood precursor peptides. (**b**) Workflow of bioinformatics and algorithmic logic. RRE domains predicted by RRE-Finder were annotated by RODEO to identify hypothetical precursors in intergenic ORFs. The RRE domains were sorted into families using an all-by-all BLAST search and the predicted ORFs were also sorted into clusters. For each RRE family, putative precursor peptide clusters were filtered for ORFs in the same genomic neighborhood, and the sizes of the families were compared. Small RRE families were discarded from the algorithm. Cases where the size of the largest precursor peptide cluster matched the RRE family were output from the algorithm for further analysis. All other cases were re-entered into the algorithm with a higher bitscore threshold for RRE family generation.

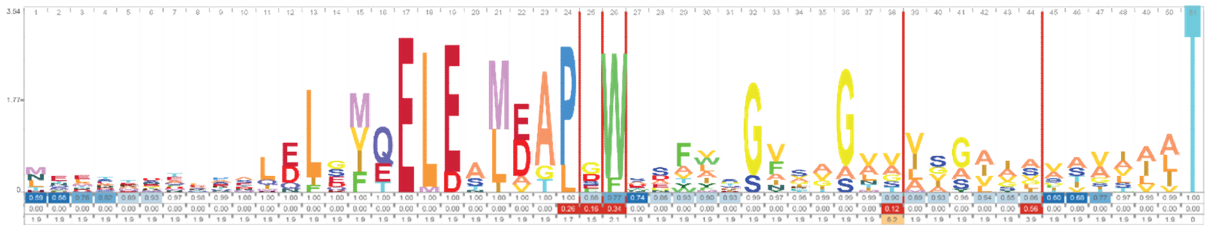

Supplementary Figure 2: Daptide precursor peptide sequence logo. The daptide precursor peptide pHMM (n = 184) was generated from precursor peptides identified by the workflow in **Supplementary Figure 1**. The pHMM was converted to a sequence logo using Skylign[9] with the information content above background setting, and the direct output is provided. The invariant Thr residue at the C-terminus is evident (cyan color), along with highly conserved features in the N-terminal leader region.

a

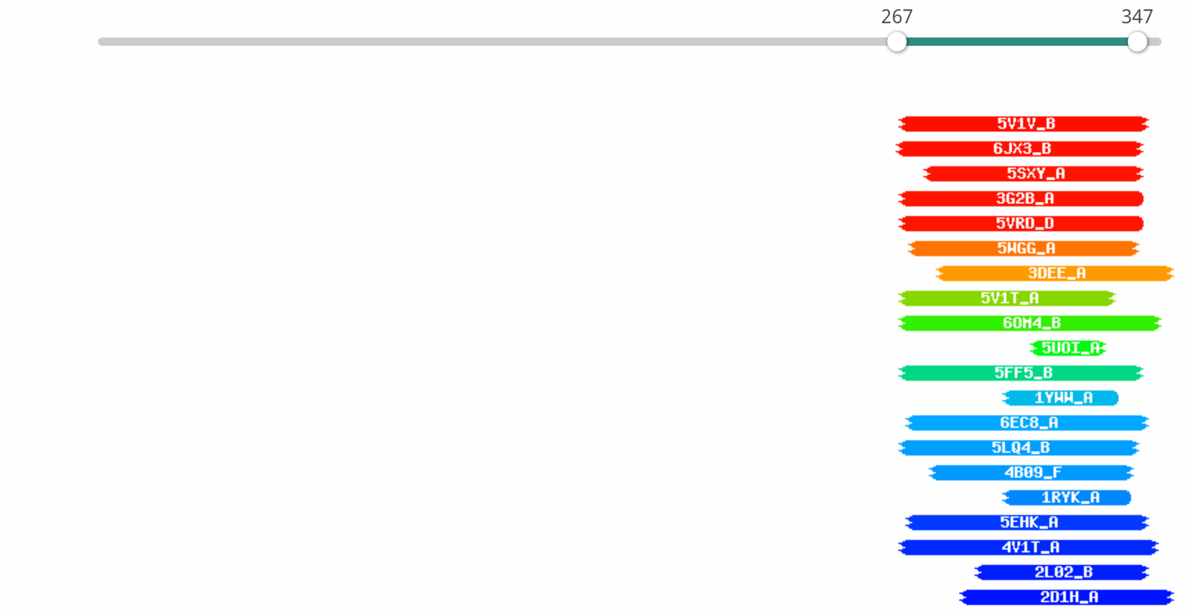

b

Hitlist

Show 25 Entries Search:

Nr	Hit	Name	Probability	E-value	Score	SS	Aligned cols	Target Length
1	5V1V_B	TbiB1; Lasso Peptide, RiPP, Peptide Binding, PROTEIN BINDING; 1.35A {Thermobaculum terrenum (strain ATCC BAA-798 / YNP1)	98.85	1e-7	72.32	12.4	81	91
2	6JX3_B	TfuB1; lasso peptide, RRE, PEPTIDE BINDING PROTEIN; 1.7A {Thermobifida fusca}	98.64	8e-7	67.55	11.7	80	99
3	5SXY_A	Bifunctional coenzyme PQQ synthesis protein C/D; RiPP RRE peptide scaffolding, CHAPERONE; NMR {Methylobacterium extorque	98.35	0.0000077	61.76	10.3	69	94
4	3G2B_A	Coenzyme PQQ synthesis protein D; helix-turn-helix, PQQ biosynthesis, BIOSYNTHETIC PROTEIN; 1.66A {Xanthomonas campestri	98.34	0.0000082	62.04	10.3	78	95
5	5VRD_D	Bifunctional coenzyme PQQ synthesis protein C/D; PQQ, oxidase, alpha-helical bundle, OXIDOREDUCTASE; 2.85A {Methylobacte	98.33	0.0000043	82.98	11.1	78	392

Supplementary Figure 3: HHpred results for MpaB. (**a**) Depicted annotation of MpaB (RefSeq ID: WP_060922562.1) analyzed using HHpred[10] with the PDB_mmCIF70_12_Oct database. The *N*-terminal domain of the protein is not annotated by HHpred, while the C-terminal domain is annotated with RREs. (**b**) Hitlist table of HHpred analysis.

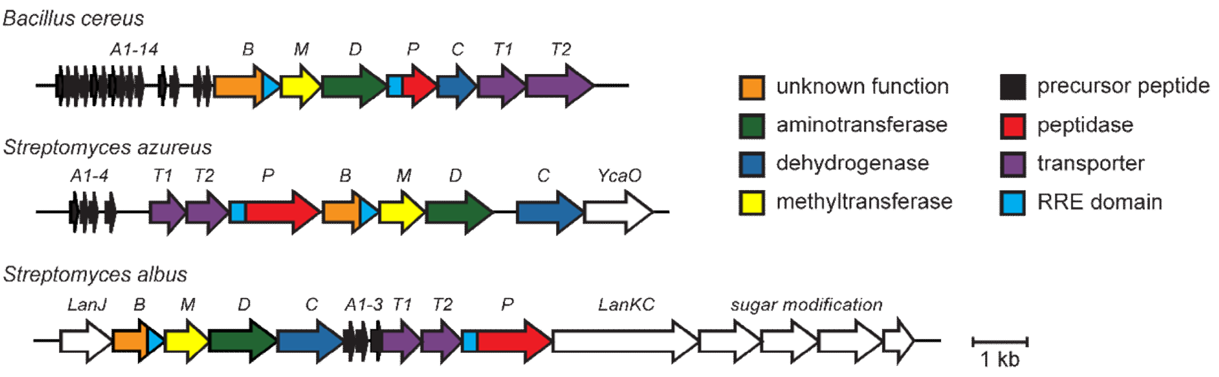

Supplementary Figure 4: Alternative identified daptide BGCs. Depicted BGCs are from two bacterial phyla (Bacillota and Actinomycetota) with differing genomic architectures, tailoring genes, and precursor peptide count.

a

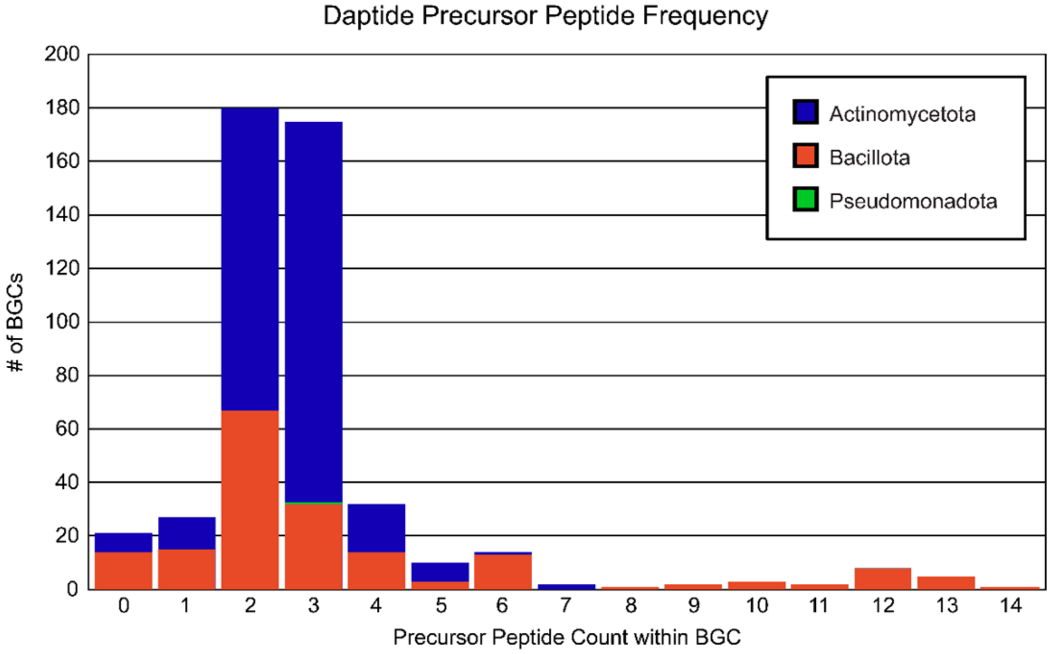

b

Precursor Peptide Count within BGC

	**0**	**1**	**2**	**3**	**4**	**5**	**6**	**7**	**8**	**9**	**10**	**11**	**12**	**13**	**14**	**Total**
**Actinomycetota**	7	12	113	142	18	7	1	2								302
**Bacillota**	14	15	67	32	14	3	13		1	2	3	2	8	5	1	180
**Pseudomonadota**				1												1
**Total**	21	27	180	175	32	10	14	2	1	2	3	2	8	5	1	483

Supplementary Figure 5: Daptide precursor peptide frequency within a BGC. (**a**) Histogram of daptide precursor peptides per BGC. (**b**) Numerical values used to generate the histogram are given in table format. Each BGC that lacked an identified precursor peptide originated from a short contig, and the BGC was presumed to be incomplete.

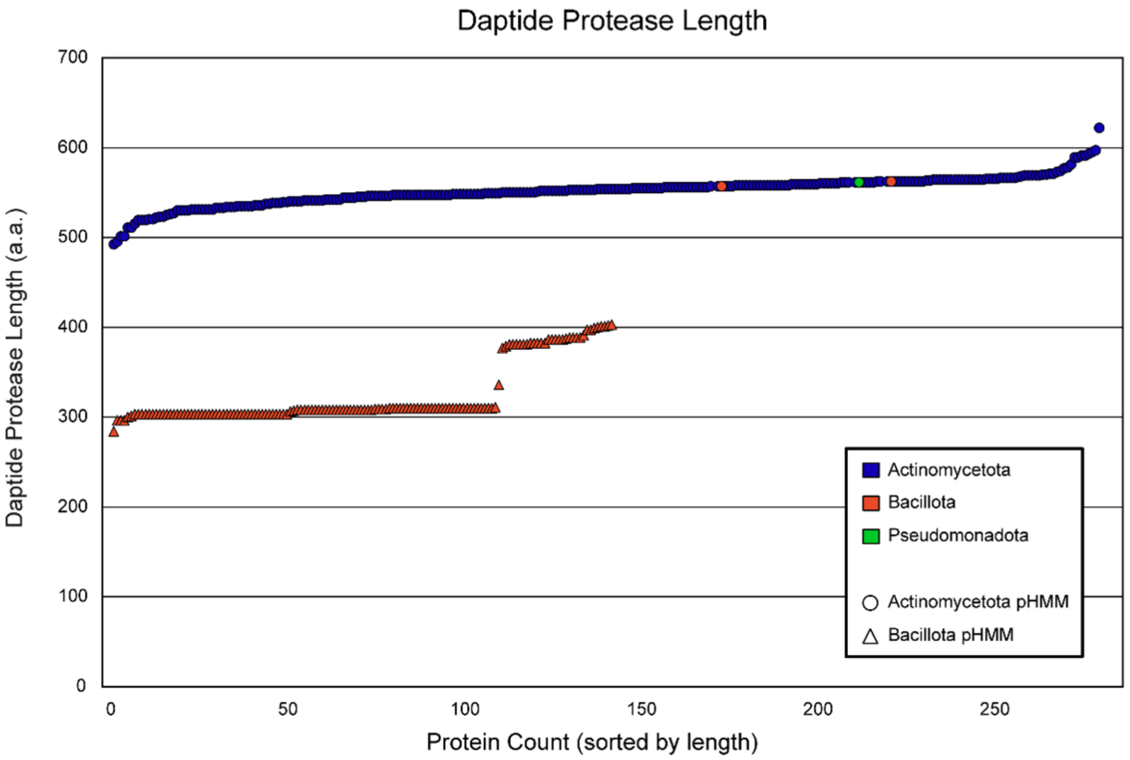

Supplementary Figure 6: Taxonomic comparison of daptide protease lengths. All full-length hits for the Actino_DapP_RRE and Bacill_DapP_RRE found using RODEO and within daptide BGCs were compiled. Protein lengths in amino acids (a.a.) were sorted into two series based on which pHMM best retrieved each protein (circle and triangle) prior to sorting by length. The color coding is based on GenBank taxonomic classification.

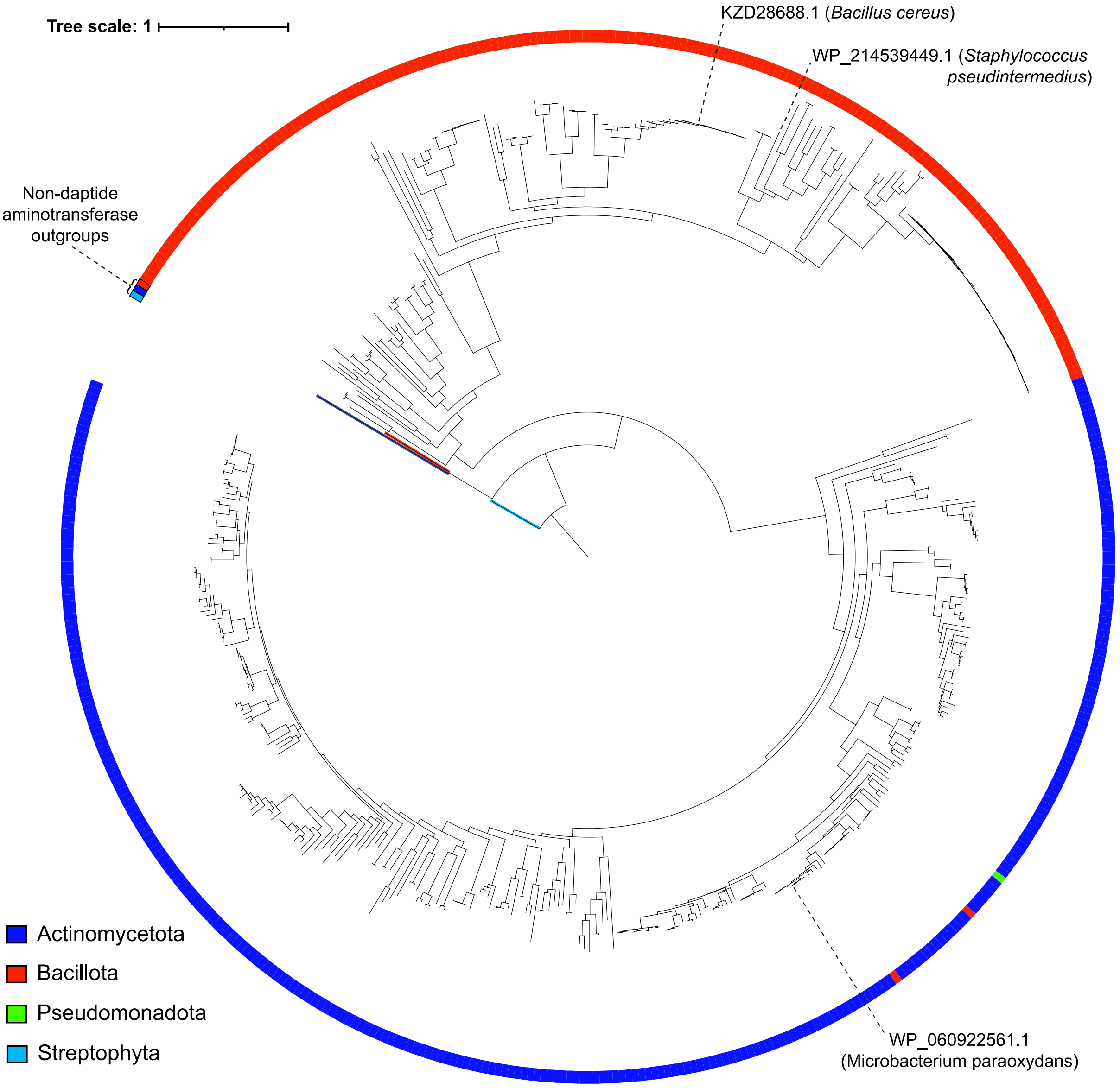

Supplementary Figure 7: Phylogenetic tree of aminotransferases. Maximum-likelihood phylogenetic tree of all 483 identified daptide aminotransferases and three outgroup aminotransferases (from aminotransferase class-III[11]). The tree is rooted using gamma-aminobutyrate transaminase 3 (UniProt/GenBank CDS identifiers: Q84P52/BAG16484.1 - Streptophyta). Two additional outgroups are included (P9WQ81/CCP44332.1 - Actinomycetota and P53555/AAB17458.1 - Bacillota) that perform related reactions in biotin biosynthesis.

a

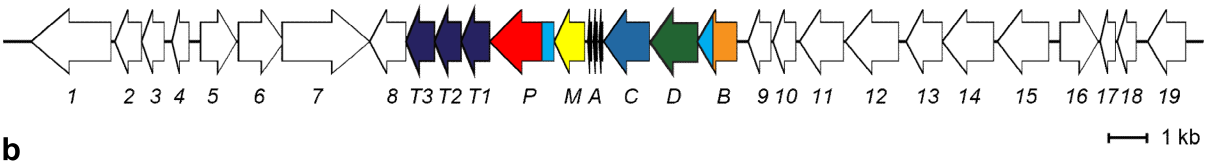

b

Gene	NCBI Accession	Description (per NCBI listing)
orf1	WP_060923289.1	Phosphate acetyltransferase
orf2	WP_157547021.1	Hypothetical protein
orf3	WP_060922551.1	Rhomboid family intramembrane serine protease
orf4	WP_157547022.1	Hypothetical protein
orf5	WP_060922553.1	MoxR family ATPase
orf6	WP_060922554.1	DUF58 domain-containing protein
orf7	WP_060922555.1	DUF3488 and transglutaminase-like domain-containing protein
orf8	WP_060922556.1	Rhodanese-related sulfurtransferase
mpaT3	WP_083370907.1	Hypothetical protein
mpaT2	WP_060922557.1	Hypothetical protein
mpaT1	WP_157547023.1	ATP-binding cassette domain-containing protein
mpaP	WP_060922558.1	Hypothetical protein
mpaM	WP_060922559.1	Class I SAM-dependent methyltransferase
mpaA3	N/A[a]	Precursor peptide
mpaA2	N/A[a]	Precursor peptide
mpaA1	N/A[a]	Precursor peptide
mpaC	WP_060922560.1	Iron-containing alcohol dehydrogenase
mpaD	WP_060922561.1	Aminotransferase class III-fold pyridoxal phosphate-dependent enzyme
mpaB	WP_060922562.1	Hypothetical protein
orf9	WP_174521438.1	GNAT family N-acetyltransferase
orf10	WP_060922563.1	Phosphatase PAP2 family protein
orf11	WP_060922564.1	Cystathionine gamma-synthase
orf12	WP_025104232.1	Cystathionine beta-synthase
orf13	WP_025104231.1	Carbohydrate ABC transporter permease
orf14	WP_025104230.1	Sugar ABC transporter permease
orf15	WP_060923551.1	Bacterial extracellular solute-binding protein
orf16	WP_083371072.1	LacI family transcriptional regulator
orf17	WP_025104227.1	Ribosomal protein
orf18	WP_025104226.1	Ribosomal protein
orf19	WP_157547024.1	Hypothetical protein

Supplementary Figure 8: Annotation of the cloned region from *M. paraoxydans* DSM 15019. (**a**) Gene arrangement of the cloned region. (**b**) Accession numbers and annotation of corresponding genes in NCBI. [a]Not annotated as gene.

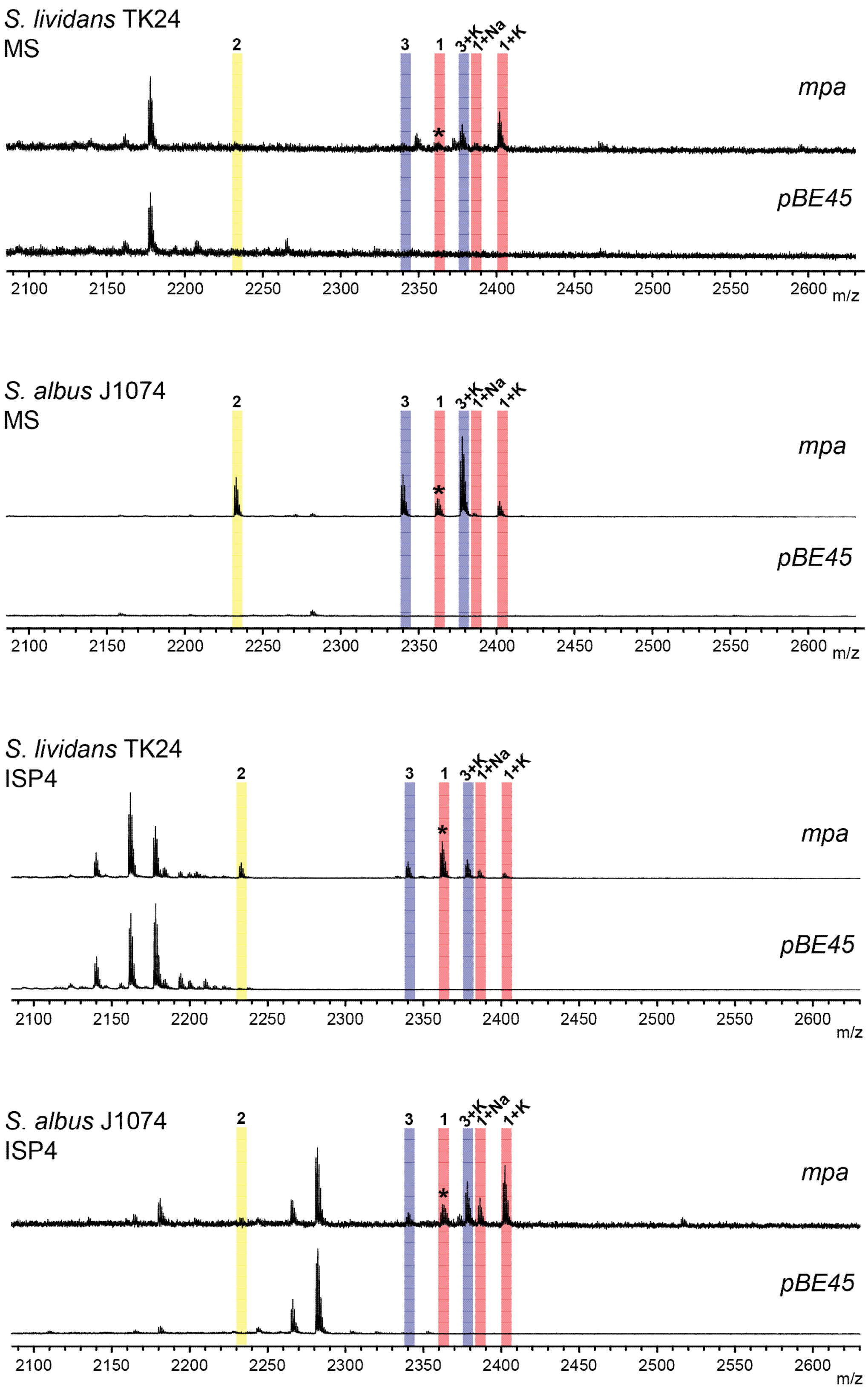

Supplementary Figure 9: Media and host screen for *mpa* BGC expression. MALDI-TOF mass spectra of methanolic extracts of *S. lividans* TK24 and *S. albus* J1074 colonies that were transformed with *mpa* or the pBE45 empty vector and cultivated on MS and ISP4 agar media. Possible overlapped peaks of [**1**+H]$^+$ and [**3**+Na]$^+$ are indicated with an asterisk.

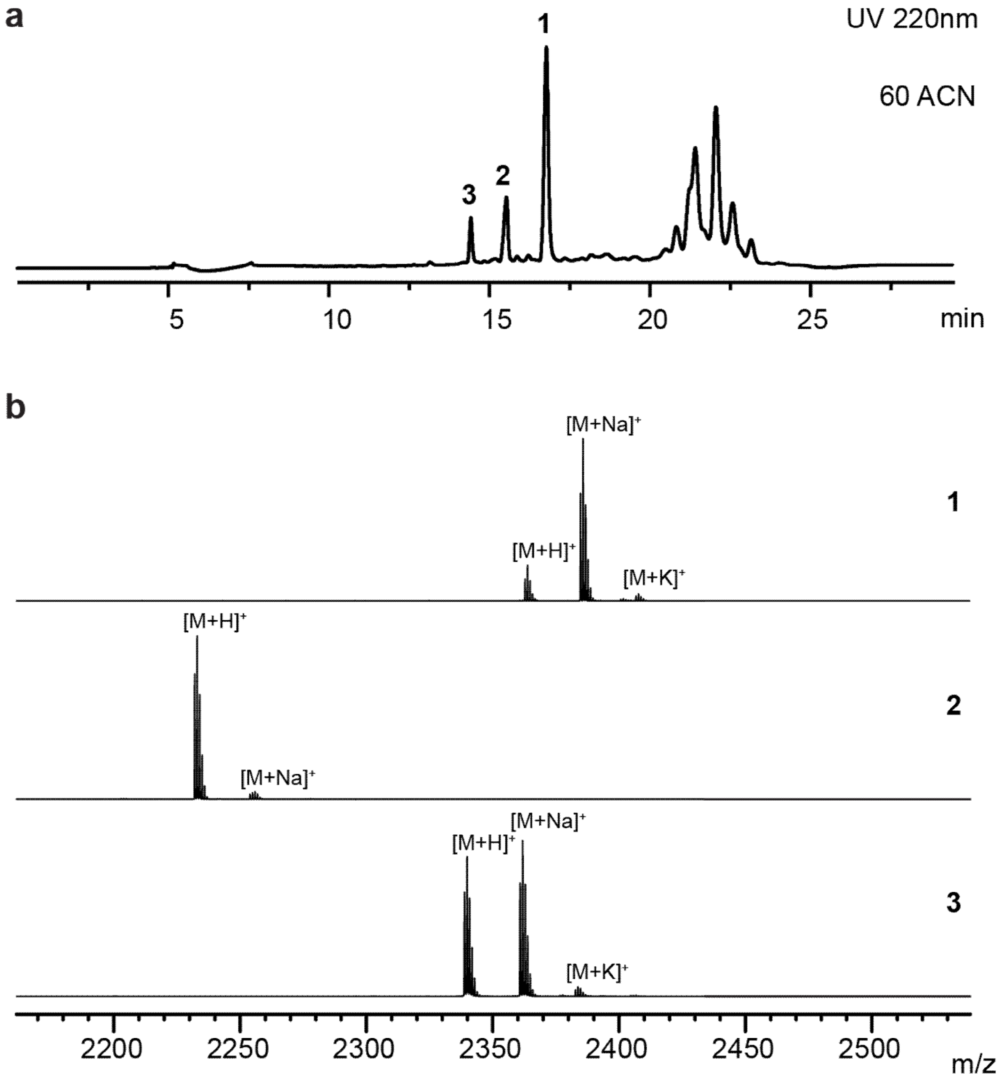

Supplementary Figure 10: Purification of daptides 1-3. (**a**) Reverse phase (C18) HPLC chromatograms with UV absorbance (220 nm) monitoring of solid phase extraction eluents using 60% acetonitrile. (**b**) MALDI-TOF-MS analysis of the collected fractions.

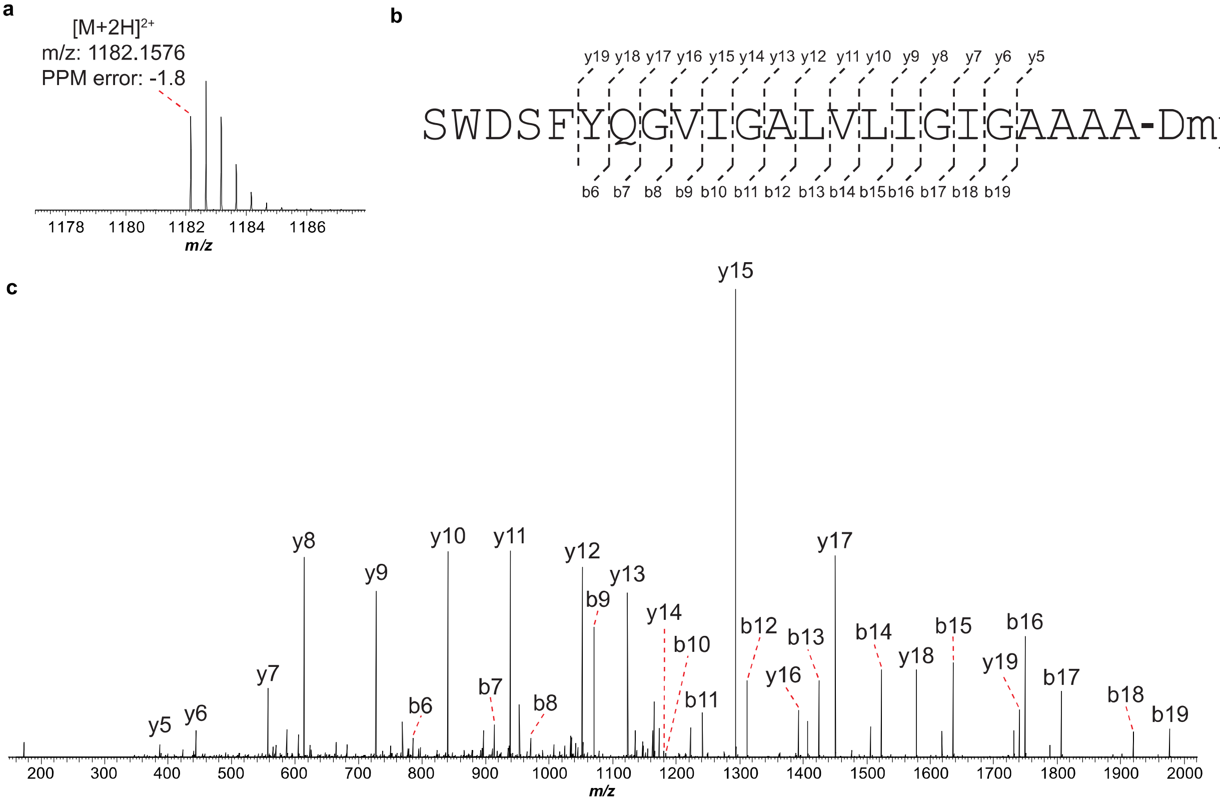

d

Ion	Calc. Mass	Obs. Mass	PPM Error
**[M+2H]$^{2+}$**	1182.1597	1182.1576	-1.8
**b6**	786.3093	786.3082	-1.4
**b7**	914.3679	914.3664	-1.6
**b8**	971.3894	971.3877	-1.8
**b9**	1070.4578	1070.4558	-1.9
**b10**	1183.5419	1183.5393	-2.2
**b11**	1240.5633	1240.5610	-1.9
**b12**	1311.6004	1311.5981	-1.8
**b13**	1424.6845	1424.6827	-1.3
**b14**	1523.7529	1523.7514	-1.0
**b15**	1636.8370	1636.8348	-1.3
**b16**	1749.9210	1749.9180	-1.7
**b17**	1806.9425	1806.9382	-2.4
**b18**	1920.0266	1920.0216	-2.6
**b19**	1977.0480	1977.0431	-2.5

Ion	Calc. Mass	Obs. Mass	PPM Error
**y5**	387.2714	387.2710	-1.0
**y6**	444.2929	444.2922	-1.6
**y7**	557.3770	557.3761	-1.6
**y8**	614.3984	614.3975	-1.5
**y9**	727.4825	727.4813	-1.6
**y10**	840.5665	840.5650	-1.8
**y11**	939.6350	939.6333	-1.8
**y12**	1052.7190	1052.7171	-1.8
**y13**	1123.7561	1123.7537	-2.1
**y14**	1180.7776	1180.7751	-2.1
**y15**	1293.8617	1293.8594	-1.8
**y16**	1392.9301	1392.9279	-1.6
**y17**	1449.9515	1449.9497	-1.2
**y18**	1578.0101	1578.0079	-1.4
**y19**	1741.0735	1741.0704	-1.8

Supplementary Figure 11: HR-MS/MS analysis of 1. (**a**) Broad band high-resolution mass spectrum of compound **1**. (**b**) Summary of collision-induced dissociation (CID) daughter ions for **1**. Observed b- and y-ions are annotated. Ions are in the +1 charge state except where indicated. Dmp: *N*2,*N*2-dimethyl-1,2-propanediamine. (**c**) CID spectrum of **1**. (**d**) Table of daughter ion assignments for **1**.

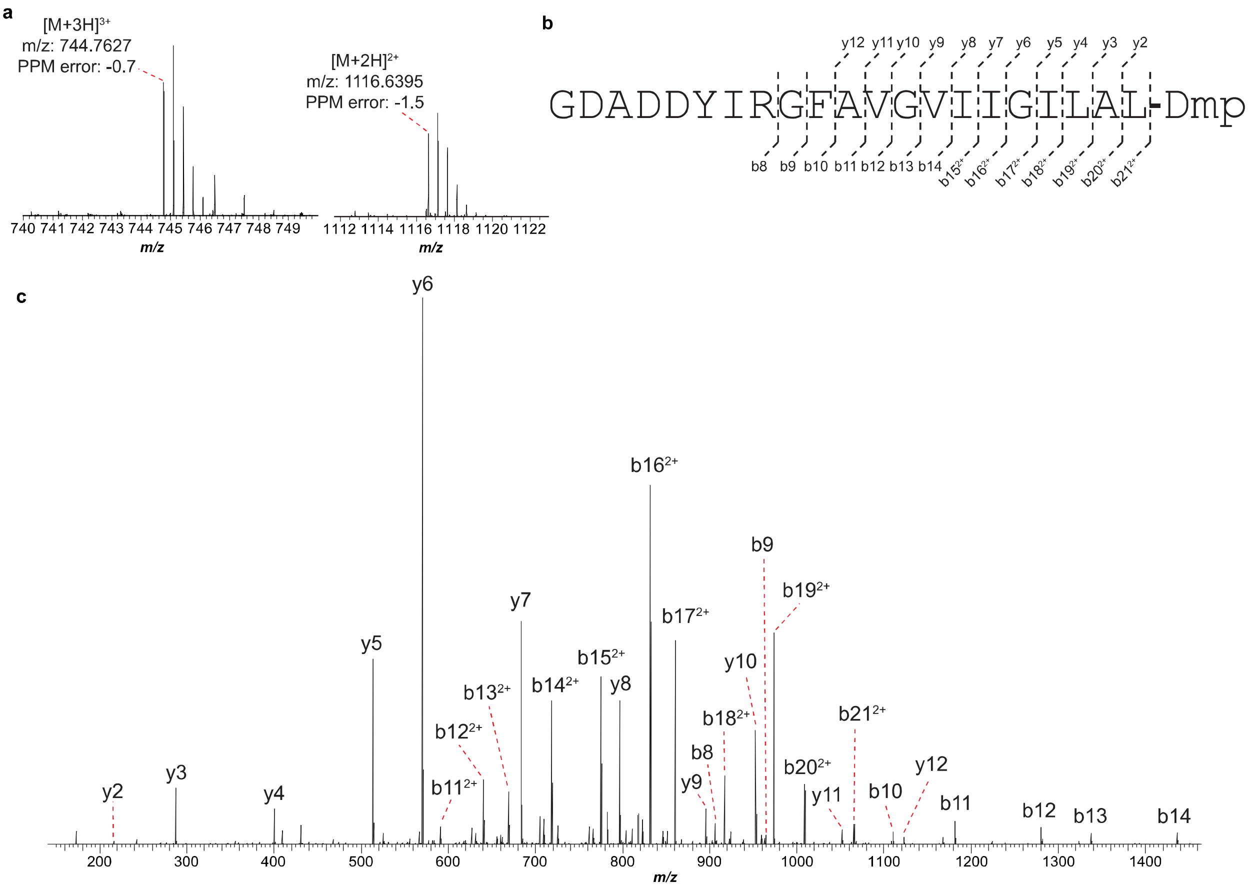

d

Ion	Calc. Mass	Obs. Mass	PPM Error
$[M+3H]^{3+}$	744.7632	744.7627	-0.7
$[M+2H]^{2+}$	1116.6412	1116.6395	-1.5
b8	906.3952	906.3939	-1.4
b9	963.4167	963.4150	-1.8
b10	1110.4851	1110.4829	-2.0
b11	1181.5222	1181.5196	-2.2
$b11^{2+}$	591.2647	591.2639	-1.4
b12	1280.5906	1280.5882	-1.9
$b12^{2+}$	640.7989	640.7981	-1.3
b13	1337.6121	1337.6095	-1.9
$b13^{2+}$	669.3097	669.3088	-1.3
b14	1436.6805	1436.6791	-1.0
$b14^{2+}$	718.8439	718.8428	-1.5
$b15^{2+}$	775.3859	775.3849	-1.3
$b16^{2+}$	831.9279	831.9269	-1.2
$b17^{2+}$	860.4387	860.4372	-1.7

Ion	Calc. Mass	Obs. Mass	PPM Error
$b18^{2+}$	916.9807	916.9793	-1.5
$b19^{2+}$	973.5227	973.5211	-1.7
$b20^{2+}$	1009.0413	1009.0397	-1.6
$b21^{2+}$	1065.5833	1065.5812	-2.0
y2	216.2067	216.2067	-1.4
y3	287.2438	287.2438	-1.4
y4	400.3277	400.3277	-1.2
y5	513.4116	513.4116	-1.4
y6	570.4330	570.4330	-1.2
y7	683.5168	683.5168	-1.5
y8	796.6007	796.6007	-1.5
y9	895.6689	895.6689	-1.6
y10	952.6902	952.6902	-1.6
y11	1051.7580	1051.7580	-2.1
y12	1122.7951	1122.7951	-2.0

Supplementary Figure 12: HR-MS/MS analysis of 2. (**a**) Broad band high-resolution mass spectrum of compound **2**. The $[M+3H]^{3+}$ ion was selected for fragmentation. (**b**) Summary of collision-induced dissociation (CID) daughter ions for **2**. Observed b- and y-ions are annotated. Ions are in the +1 charge state except where indicated. Dmp: N_2,N_2-dimethyl-1,2-propanediamine. (**c**) CID spectrum of **2**. (**d**) Table of daughter ion assignments for **2**.

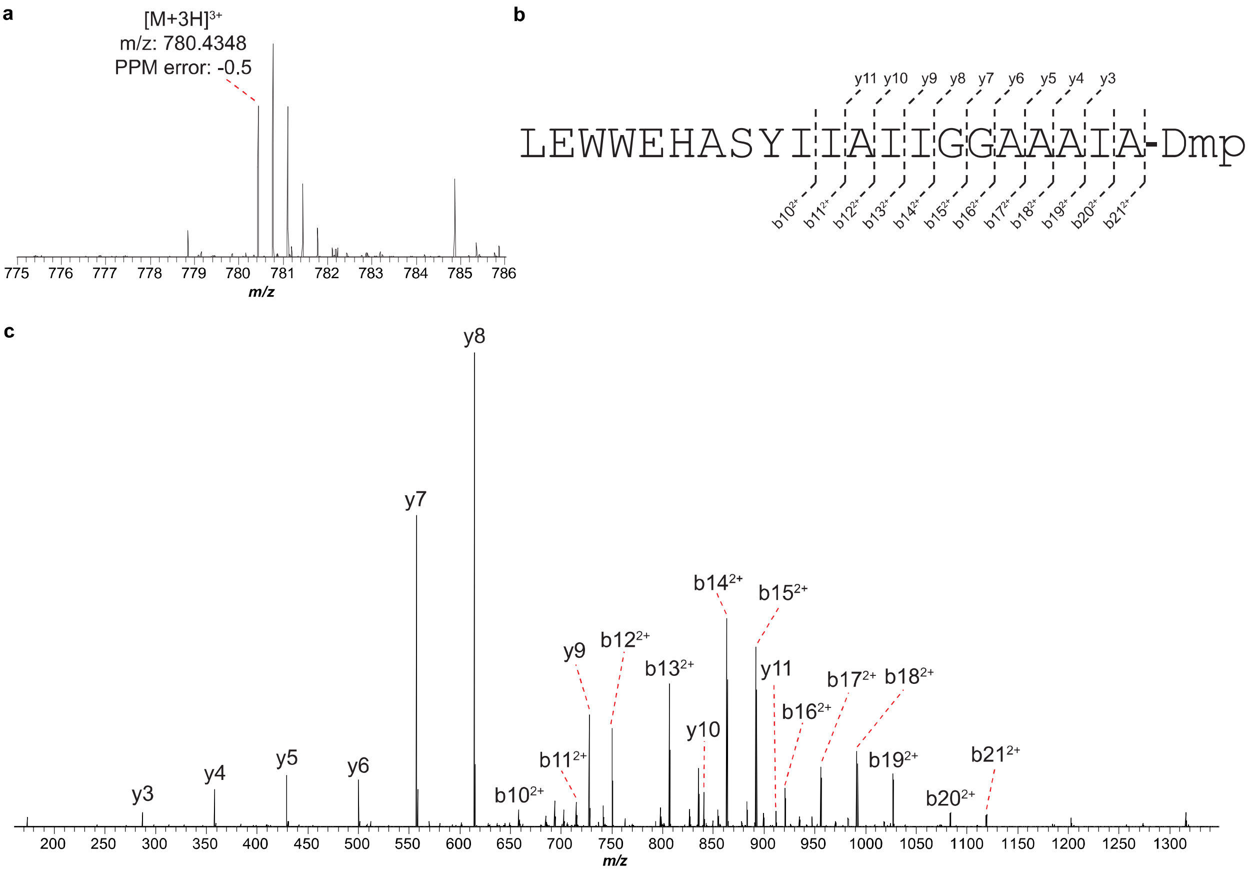

d

Ion	Calc. Mass	Obs. Mass	PPM Error
**$[M+3H]^{3+}$**	780.4352	780.4348	-0.5
**$b10^{2+}$**	658.3089	658.3079	-1.6
**$b11^{2+}$**	714.8510	714.8497	-1.8
**$b12^{2+}$**	750.3695	750.3681	-1.9
**$b13^{2+}$**	806.9115	806.9101	-1.8
**$b14^{2+}$**	863.4536	863.4519	-2.0
**$b15^{2+}$**	891.9643	891.9625	-2.1
**$b16^{2+}$**	920.4750	920.4732	-2.0
**$b17^{2+}$**	955.9936	955.9916	-2.1
**$b18^{2+}$**	991.5122	991.5101	-2.1
**$b19^{2+}$**	1027.0307	1027.0284	-2.3

Ion	Calc. Mass	Obs. Mass	PPM Error
**$b20^{2+}$**	1083.5727	1083.5702	-2.3
**$b21^{2+}$**	1119.0913	1119.0885	-2.5
**y3**	287.2442	287.2436	-2.1
**y4**	358.2813	358.2807	-1.7
**y5**	429.3184	429.3176	-1.9
**y6**	500.3555	500.3546	-1.8
**y7**	557.3770	557.3759	-2.0
**y8**	614.3984	614.3972	-2.0
**y9**	727.4825	727.4812	-1.8
**y10**	840.5665	840.5648	-2.0
**y11**	911.6037	911.6019	-2.0

Supplementary Figure 13: HR-MS/MS analysis of 3. (**a**) Broad band high-resolution mass spectrum of compound **3**. (**b**) Summary of collision-induced dissociation (CID) daughter ions for **3**. Observed b- and y-ions are annotated. Ions are in the +1 charge state except where indicated. Dmp: N_2,N_2-dimethyl-1,2-propanediamine. (**c**) CID spectrum of **3**. (**d**) Table of daughter ion assignments for **3**.

a

^{1}H NMR, 1,1,1,3,3,3-Hexafluoroisopropanol-d_2 (HFIP-d_2), 750 MHz. The solvent peak appears at 4.41 ppm.

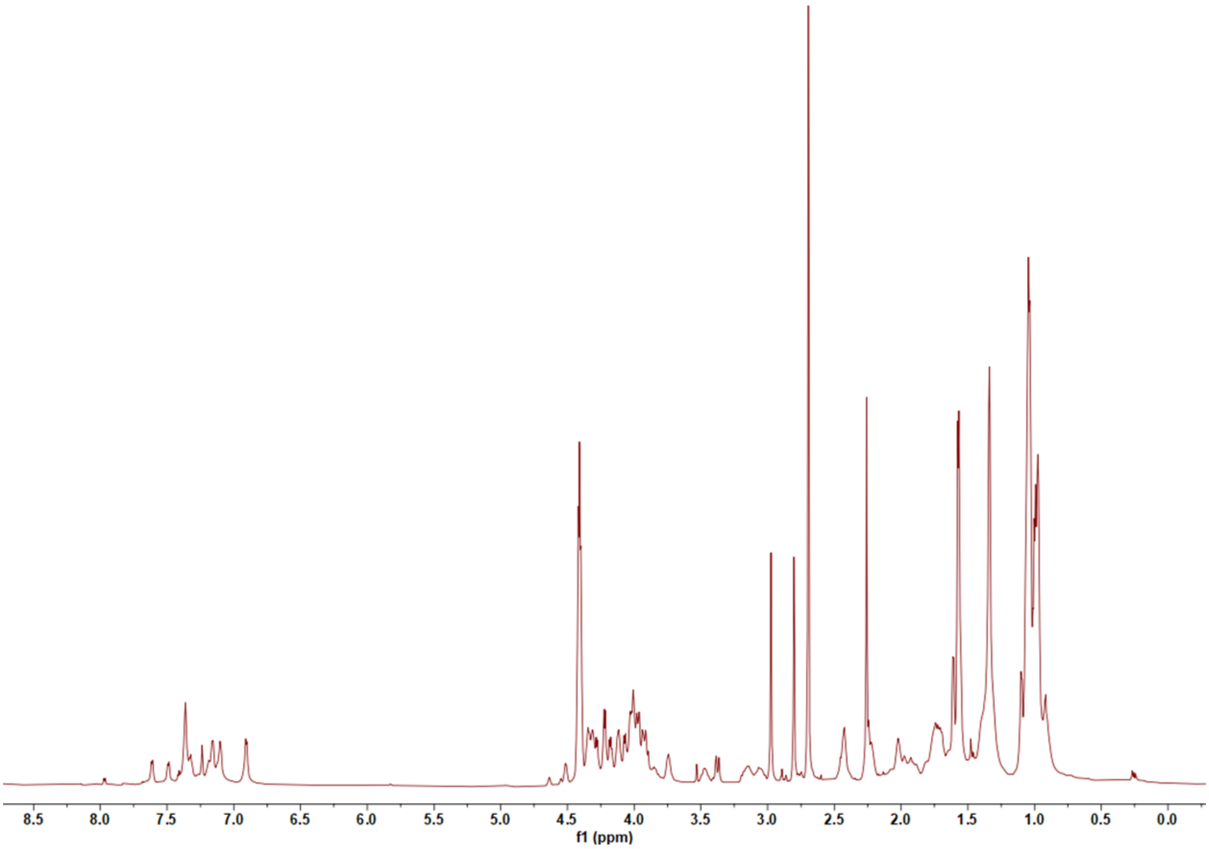

b

^{13}C NMR, 1,1,1,3,3,3-Hexafluoroisopropanol-d_2 (HFIP-d_2), 750 MHz. The solvent peaks appear at 68.1 and 120.7 ppm.

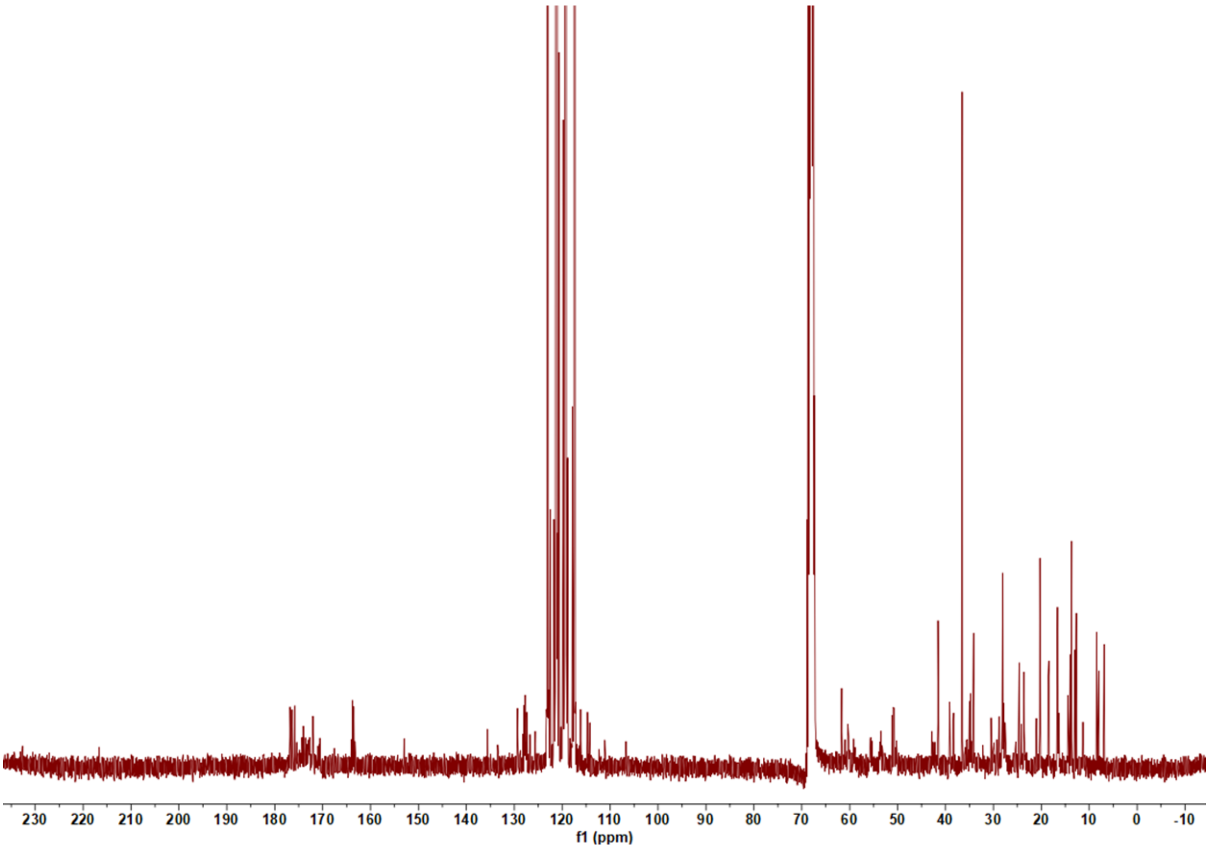

c

^{1}H-^{1}H COSY NMR, 1,1,1,3,3,3-Hexafluoroisopropanol-d_2 (HFIP-d_2), 750 MHz.

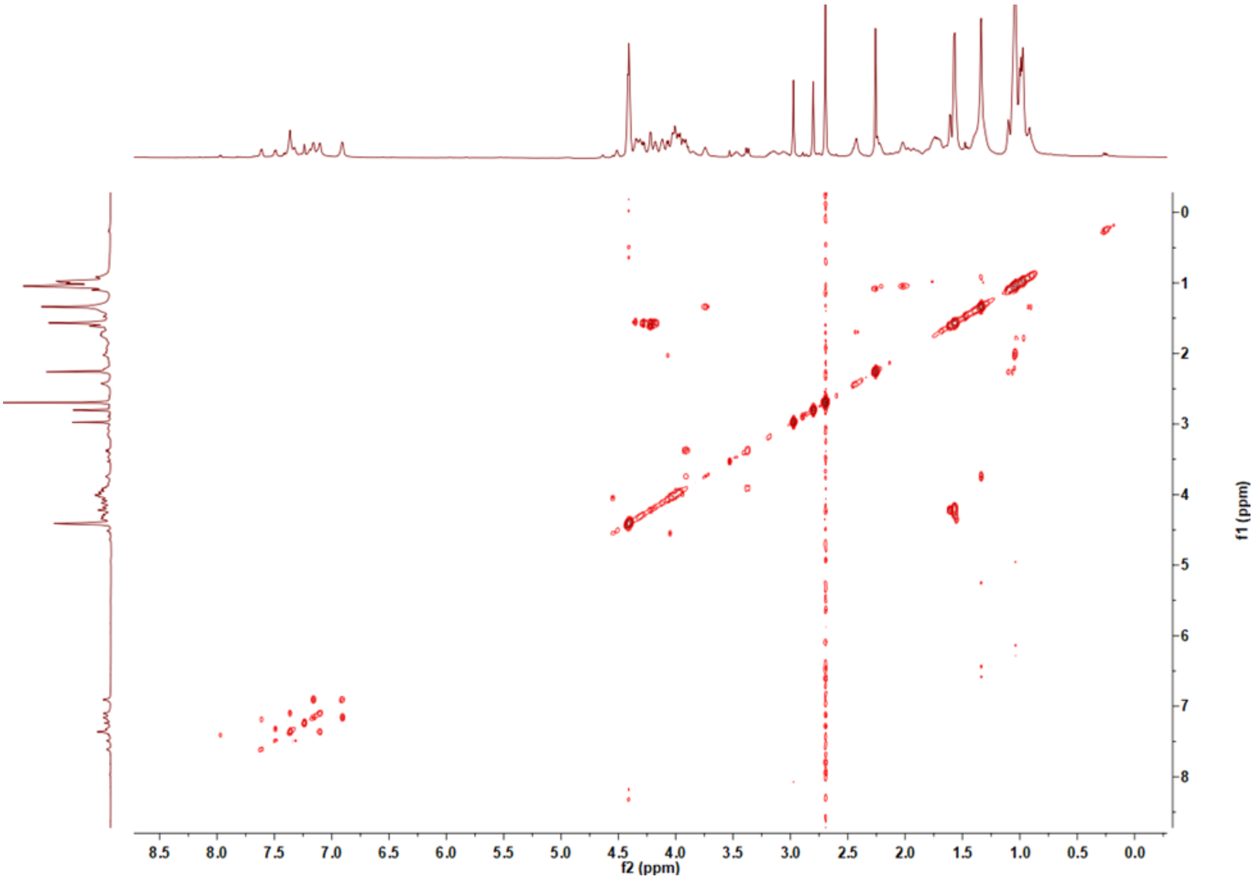

d

^{1}H-^{13}C HSQC NMR, 1,1,1,3,3,3-Hexafluoroisopropanol-d_2 (HFIP-d_2), 750 MHz. Multiplicity-edited HSQC was performed. Signals of CH_2 and CH/CH_3 are shown in blue and red, respectively.

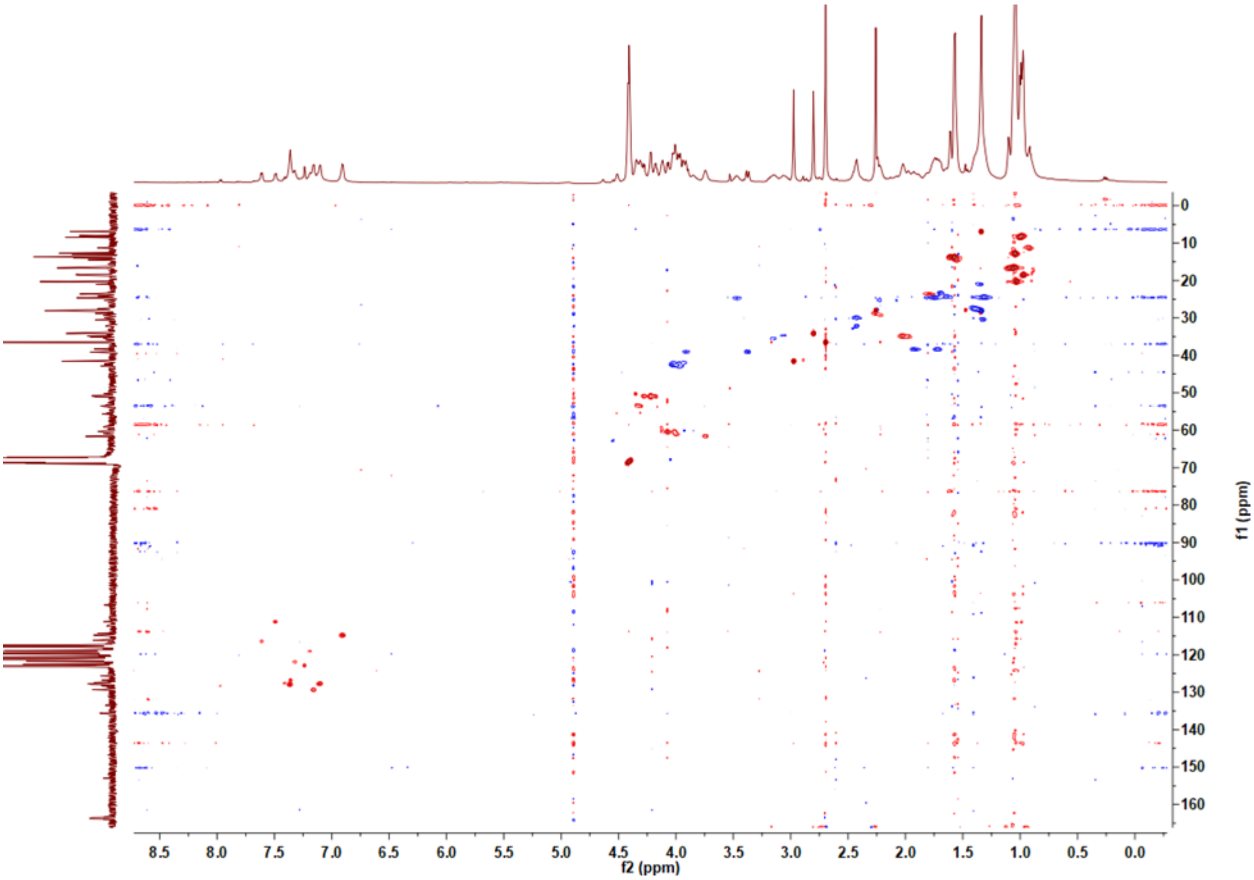

e

^{1}H-^{13}C HMBC NMR, 1,1,1,3,3,3-Hexafluoroisopropanol-d_2 (HFIP-d_2), 750 MHz.

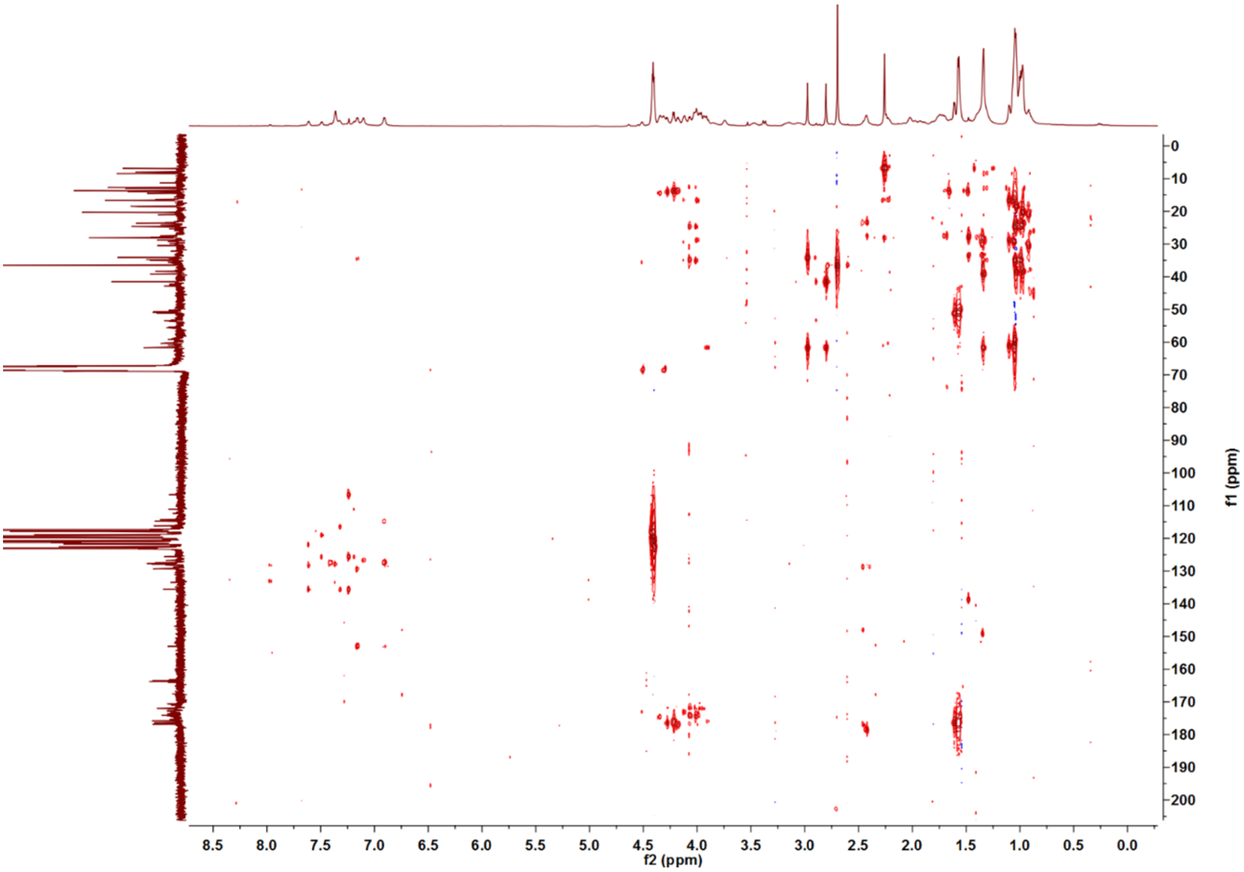

f

COSY focusing on the Dmp region. Dmp: N_2,N_2-dimethyl-1,2-propanediamine.

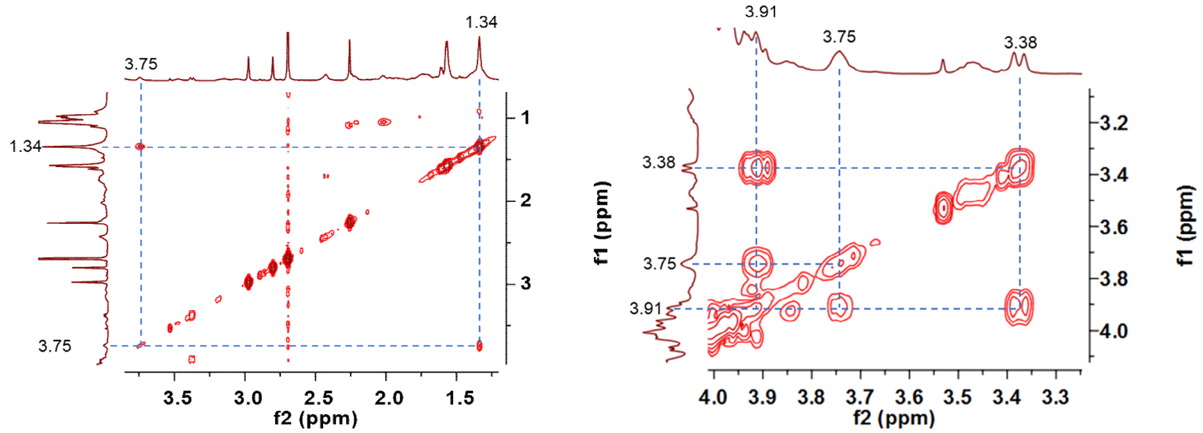

g

HSQC focusing on the Dmp region. Multiplicity-edited HSQC was performed. Signals of CH_2 and CH/CH_3 are shown in blue and red, respectively. Dmp: *N*2,*N*2-dimethyl-1,2-propanediamine.

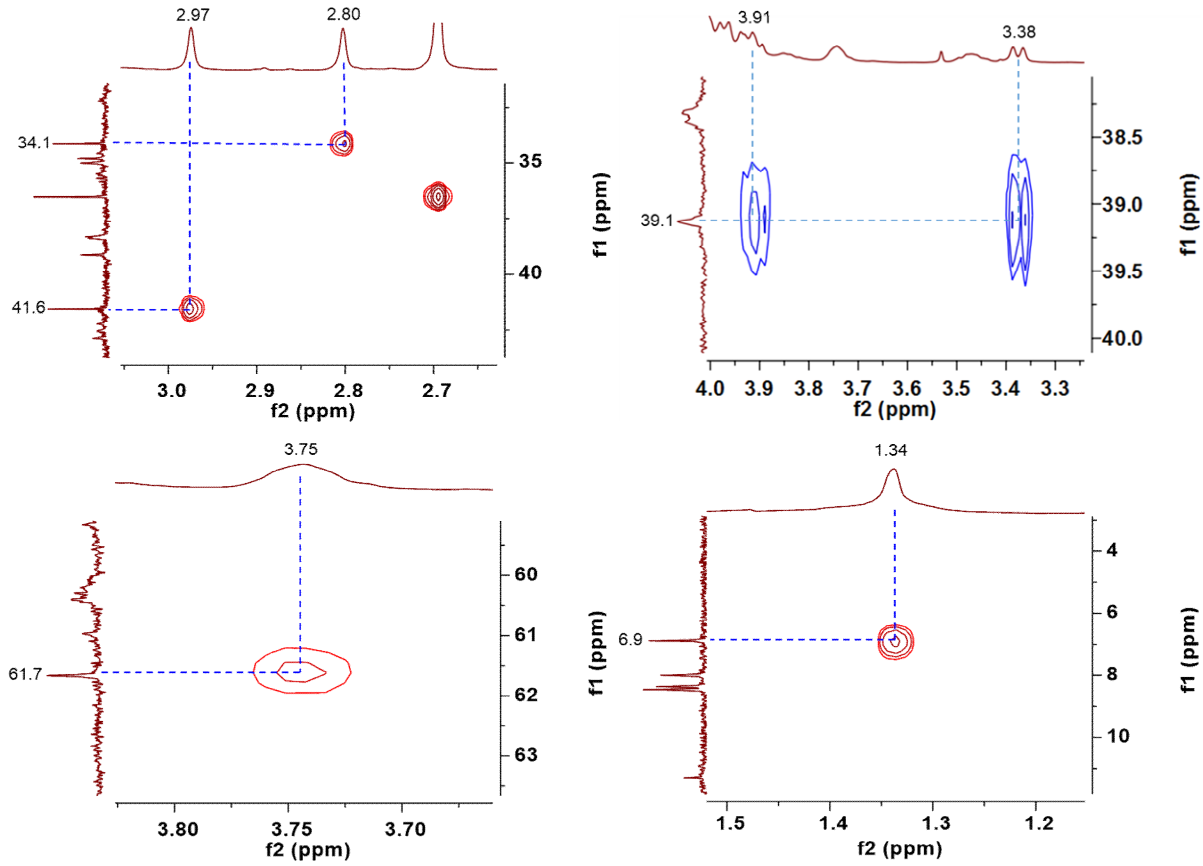

h

HMBC focusing on the Dmp region. Dmp: N_2,N_2-dimethyl-1,2-propanediamine.

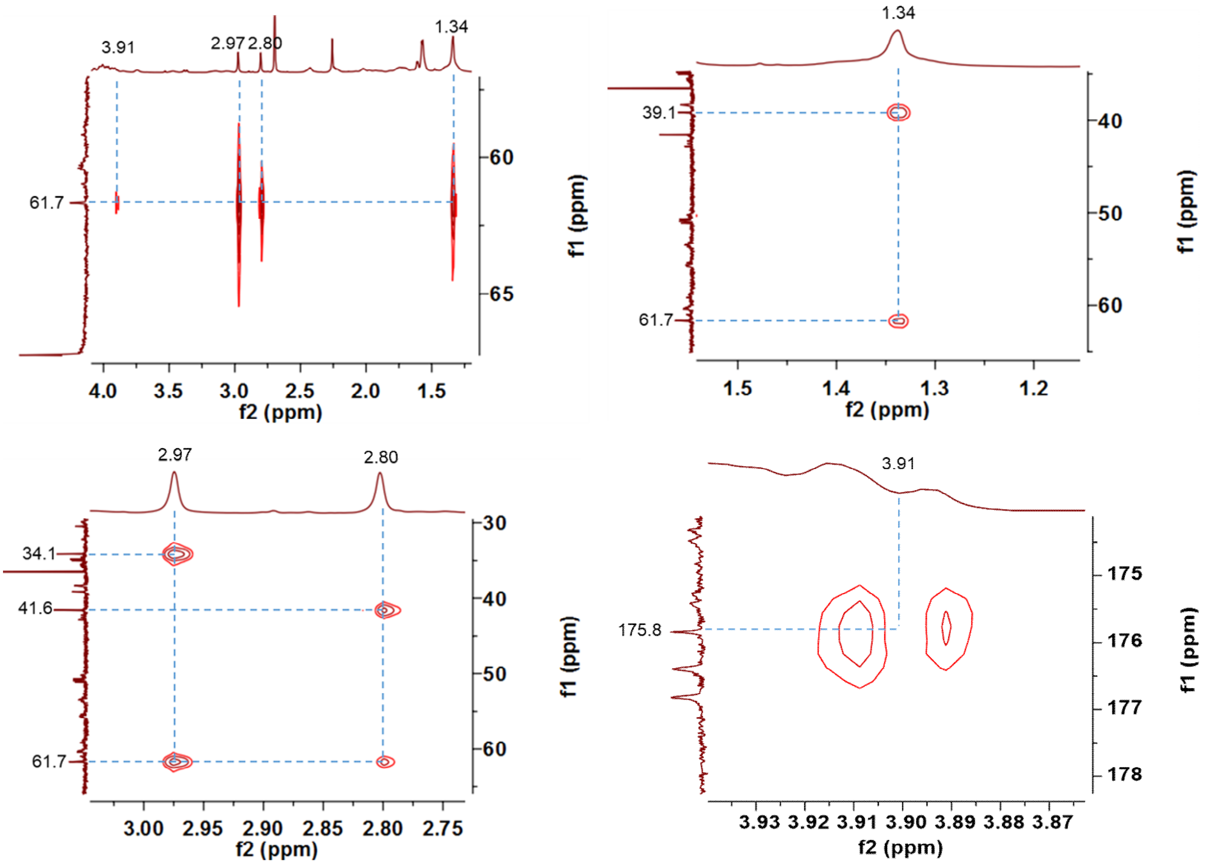

i

HSQC focusing on the Ala-23 region. Multiplicity-edited HSQC was performed. Signals of CH_2 and CH/CH_3 are shown in blue and red, respectively.

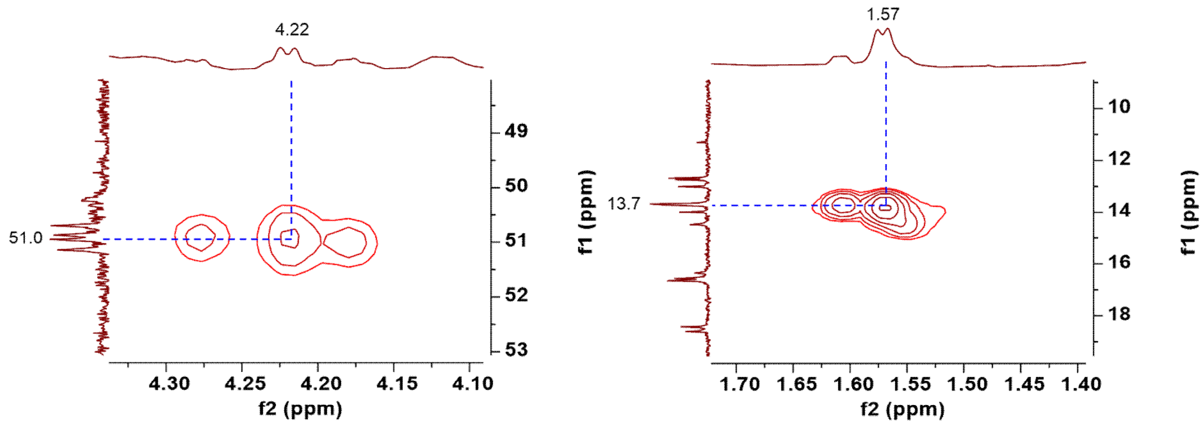

j

HMBC focusing on the Ala-23 region.

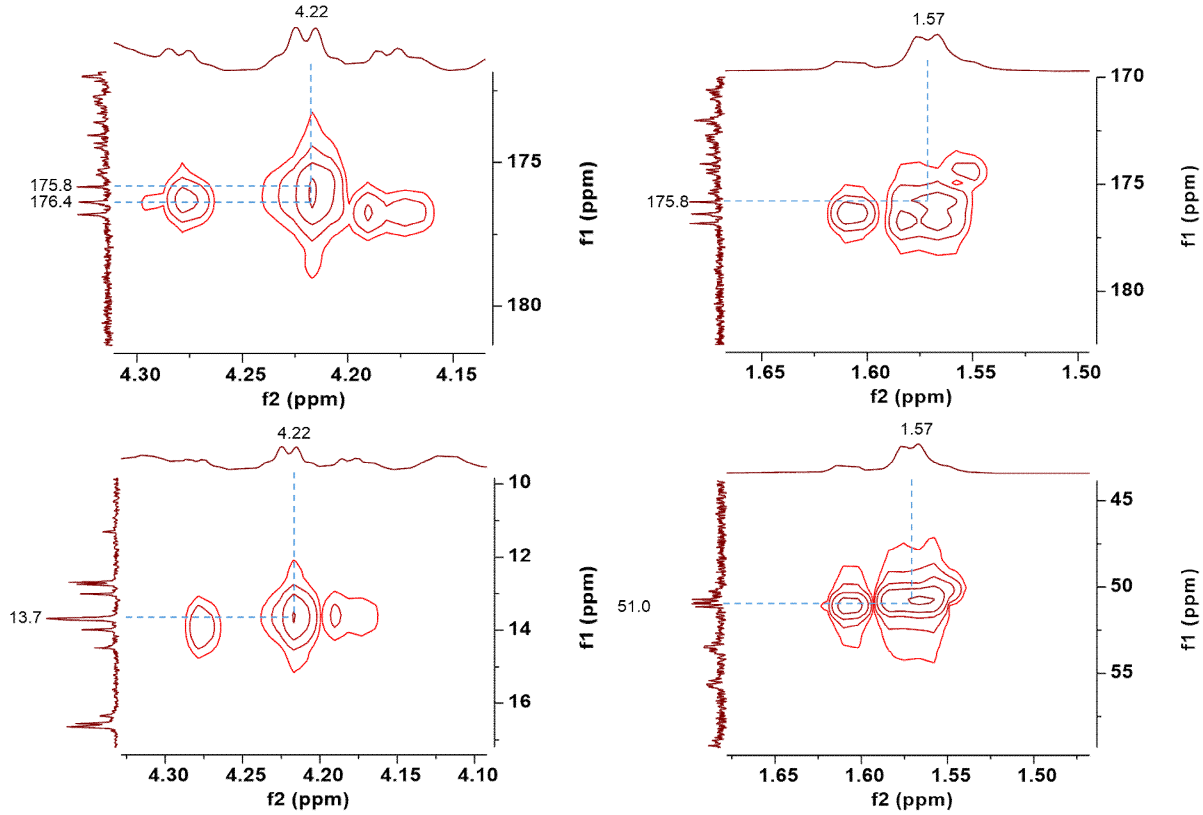

k

Summary of HMBC correlations in the Dmp and Ala-23 region. Dmp: N_2,N_2-dimethyl-1,2-propanediamine.

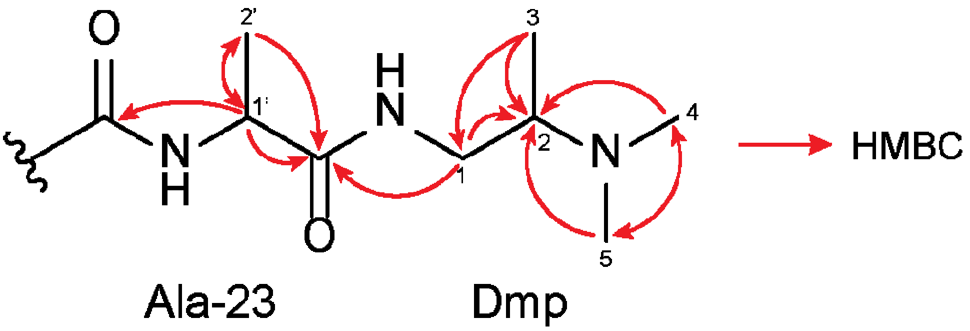

I

COSY focusing on the Trp-2 region

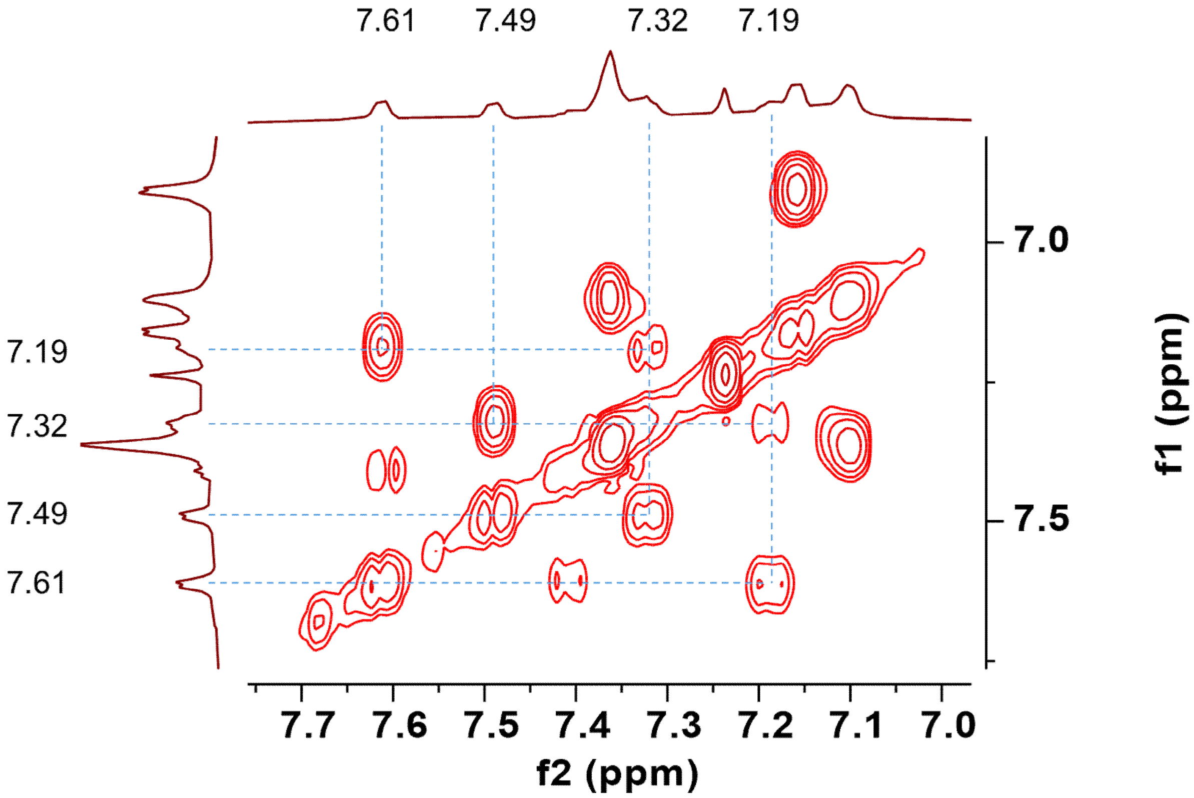

m

HSQC focusing on the Trp-2 region. Multiplicity-edited HSQC was performed. Signals of CH2 and CH/CH3 are shown in blue and red, respectively.

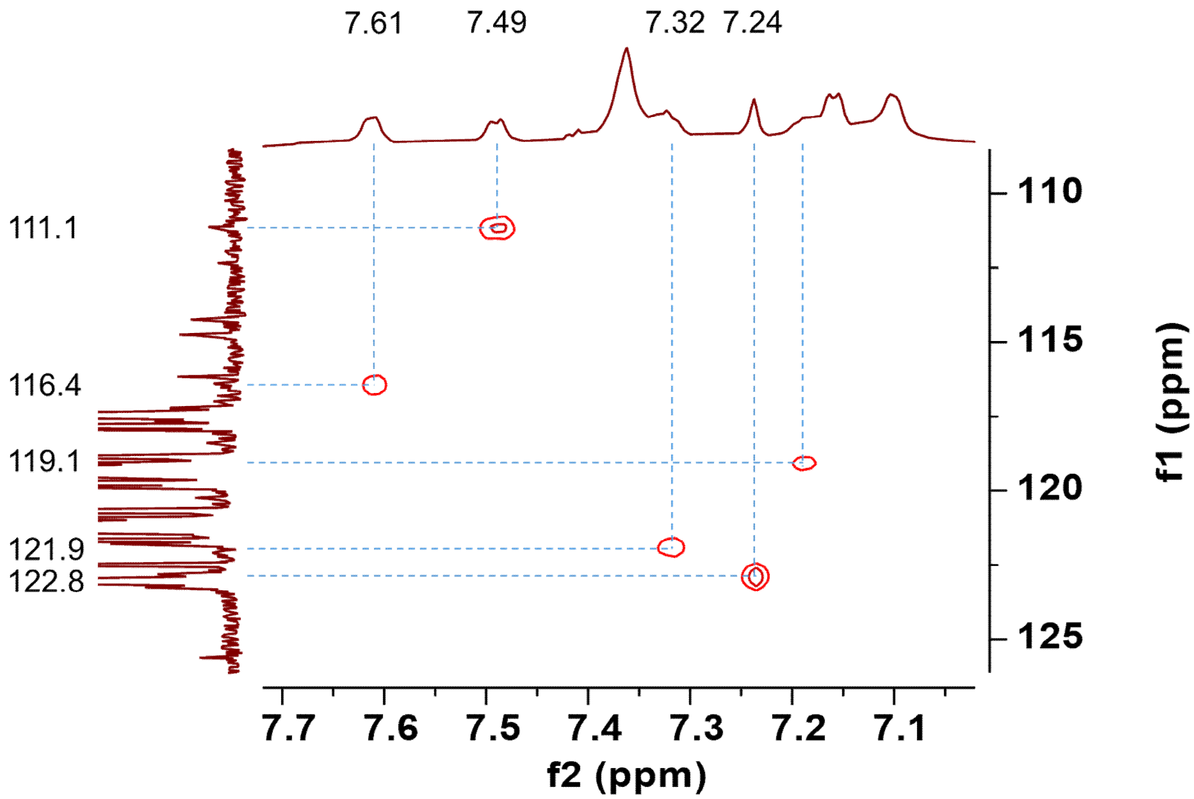

n

HMBC focusing on the Trp-2 region.

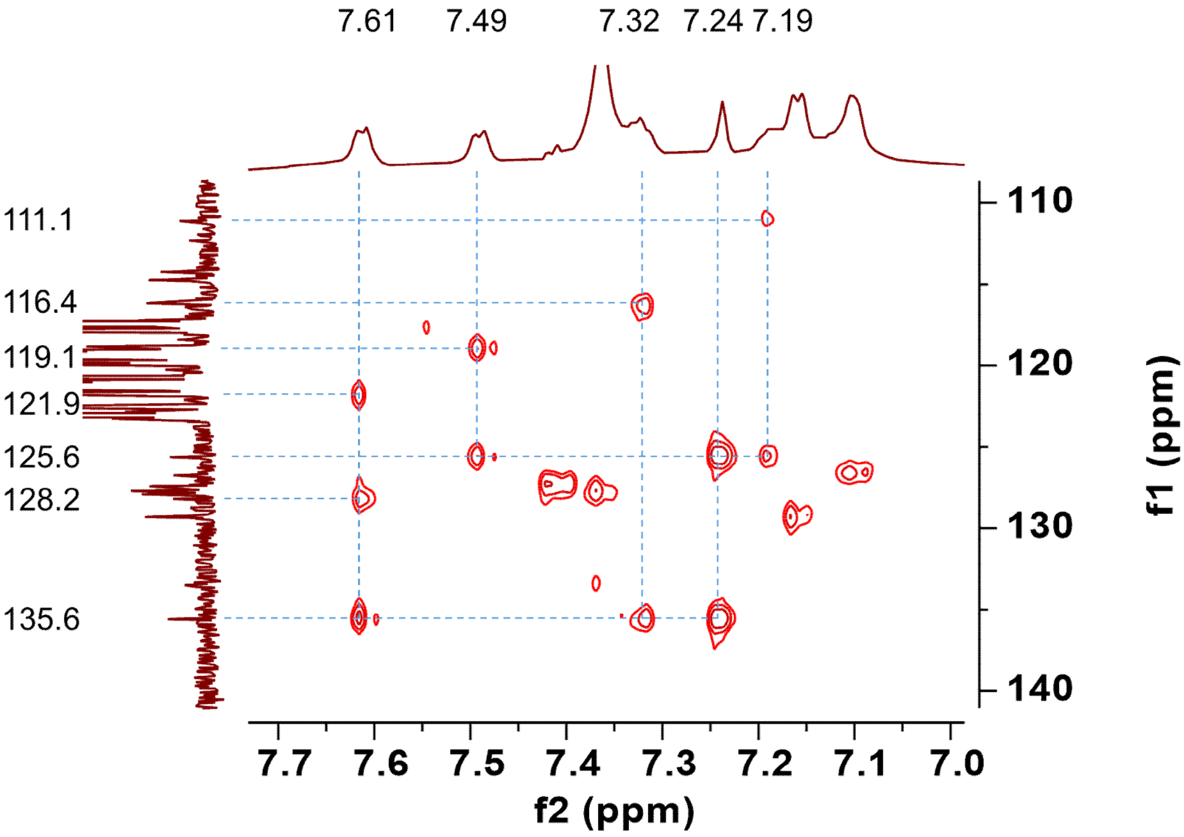

o

Summary of COSY and HMBC correlations in the Trp-2 region.

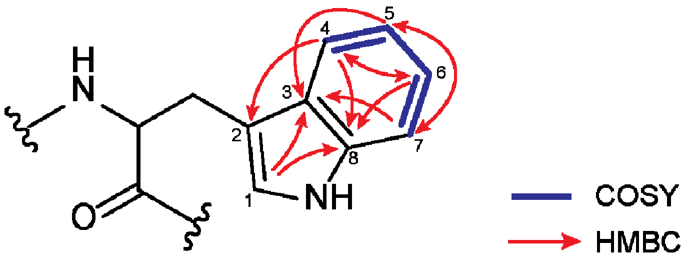

Trp-2

No.	1 (daptide)	
	δ_C type	δ_H multi
1	122.8 CH	7.24 s
2	128.2 C	
3	125.6 C	
4	116.4 CH	7.61 d (7.1)
5	119.1 CH	7.19 overlapped
6	121.9 CH	7.32 overlapped
7	111.1 CH	7.49 d (7.9)
8	135.6 C	

p

COSY focusing on the Tyr-6 region

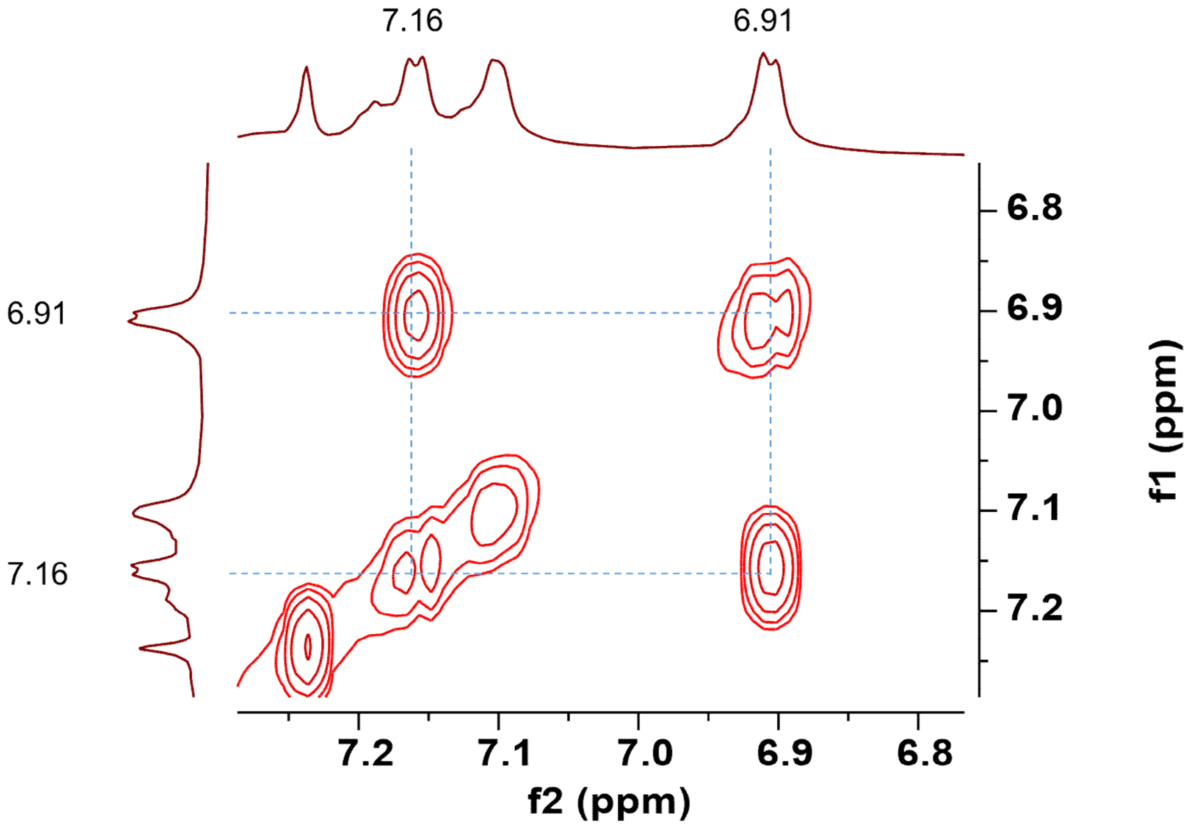

q

HSQC focusing on the Tyr-6 region. Multiplicity-edited HSQC was performed. Signals of CH2 and CH/CH3 are shown in blue and red, respectively.

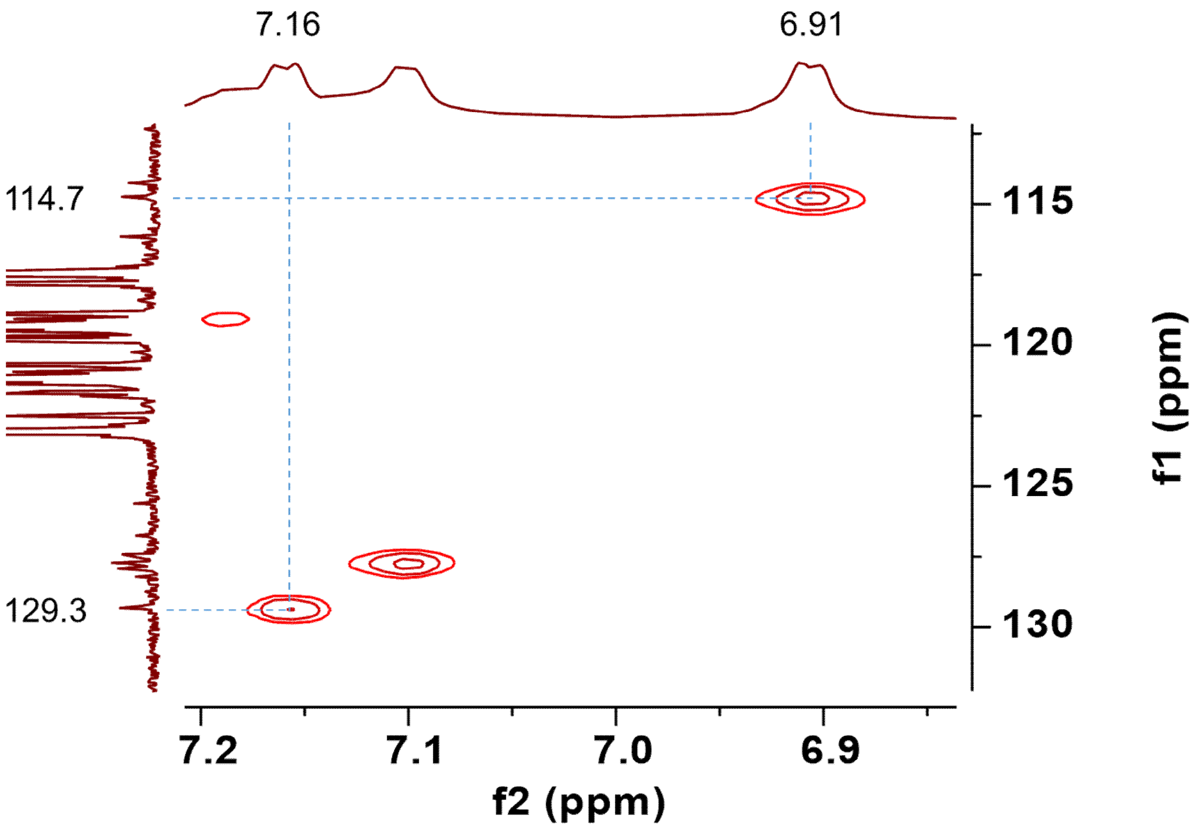

r

HMBC focusing on the Tyr-6 region.

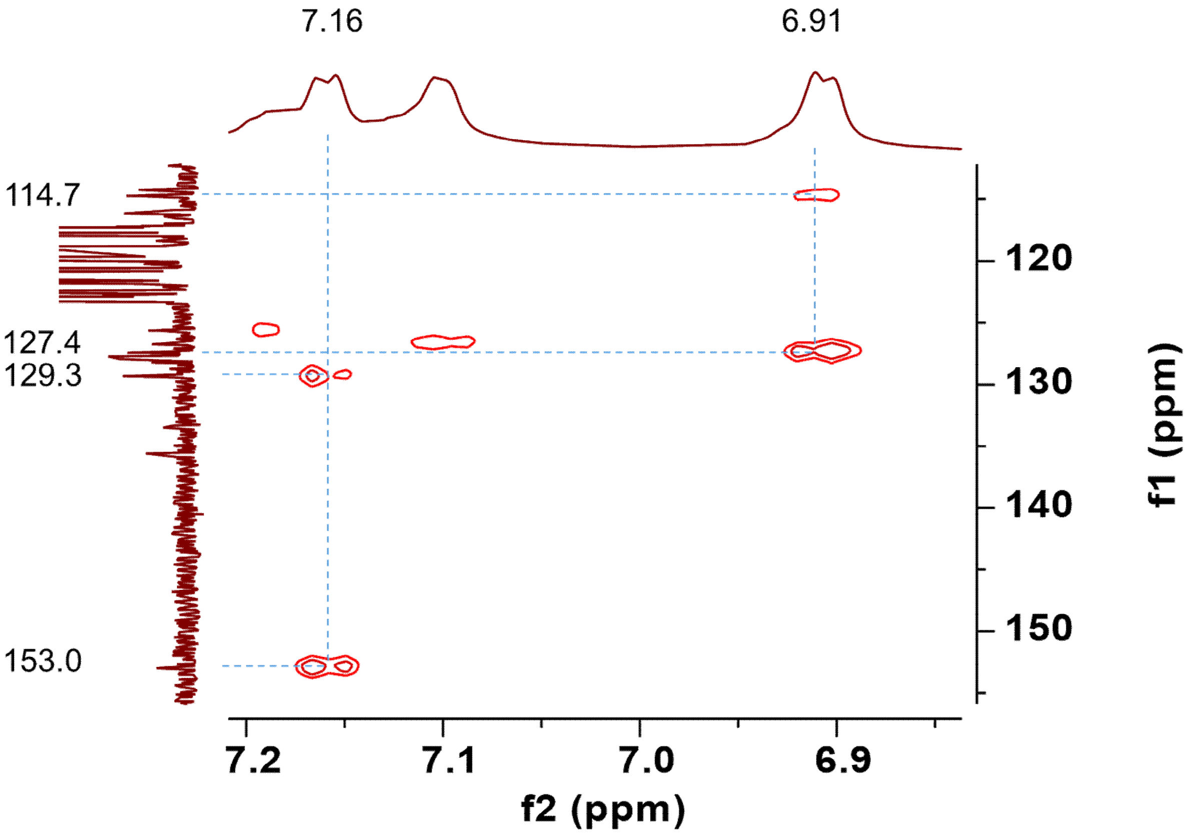

s

Summary of COSY and HMBC correlations in the Tyr-6 region.

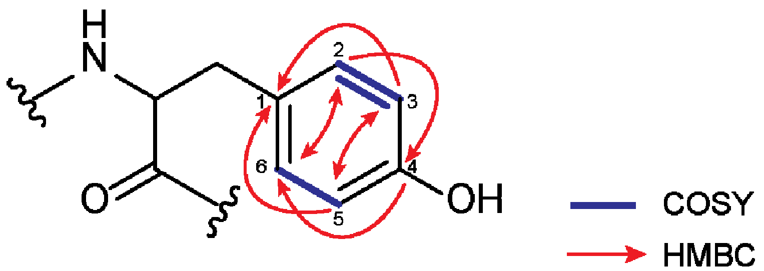

Tyr-6

No.	1 (daptide)	
	δC type	δH multi
1	127.4 C	
2	129.3 CH	7.16 overlapped
3	114.7 CH	6.91 d (7.1)
4	153.0 C	
5	114.7 CH	6.91 d (7.1)
6	129.3 CH	7.16 overlapped

Supplementary Figure 14: NMR spectra of compound 1 in 1,1,3,3,3-hexafluoroisopropanol-d2 (HFIP-*d2*). (**a**) ^{1}H NMR. The solvent peak appears at 4.41 ppm. (**b**) ^{13}C NMR. The solvent peaks appear at 68.1 and 120.7 ppm. (**c**) ^{1}H-^{1}H COSY NMR. (**d**) ^{1}H-^{13}C HSQC NMR. Multiplicity-edited HSQC was performed. Signals of CH_2 and CH/CH_3 are shown in blue and red, respectively. (**e**) ^{1}H-^{13}C HMBC NMR. (**f**) COSY focusing on the Dmp region. Dmp: N_2,N_2-dimethyl-1,2-propanediamine. (**g**) HSQC focusing on the Dmp region. Multiplicity-edited HSQC was performed. Signals of CH_2 and CH/CH_3 are shown in blue and red, respectively. (**h**) HMBC focusing on the Dmp region. (**i**) HSQC focusing on the Ala-20 region. Multiplicity-edited HSQC was performed. Signals of CH_2 and CH/CH_3 are shown in blue and red, respectively. (**j**) HMBC focusing on the Ala-20 region. (**k**) Summary of HMBC correlations in the Dmp and Ala-20 region. Dmp: N_2,N_2-dimethyl-1,2-propanediamine. (**l**) COSY focusing on the Trp-2 region. (**m**) HSQC focusing on the Trp-2 region. Multiplicity-edited HSQC was performed. Signals of CH2 and CH/CH3 are shown in blue and red, respectively. (**n**) HMBC focusing on the Trp-2 region. (**o**) Summary of COSY and HMBC correlations in the Trp-2 region. (**p**) COSY focusing on the Tyr-6 region (**q**) HSQC focusing on the Tyr-6 region. Multiplicity-edited HSQC was performed. Signals of CH2 and CH/CH3 are shown in blue and red, respectively. (**r**) HMBC focusing on the Tyr-6 region. (**s**) Summary of COSY and HMBC correlations in the Tyr-6 region.

a

(*R*)-Dmp ^{1}H NMR, DMSO-d_6, 500 MHz.

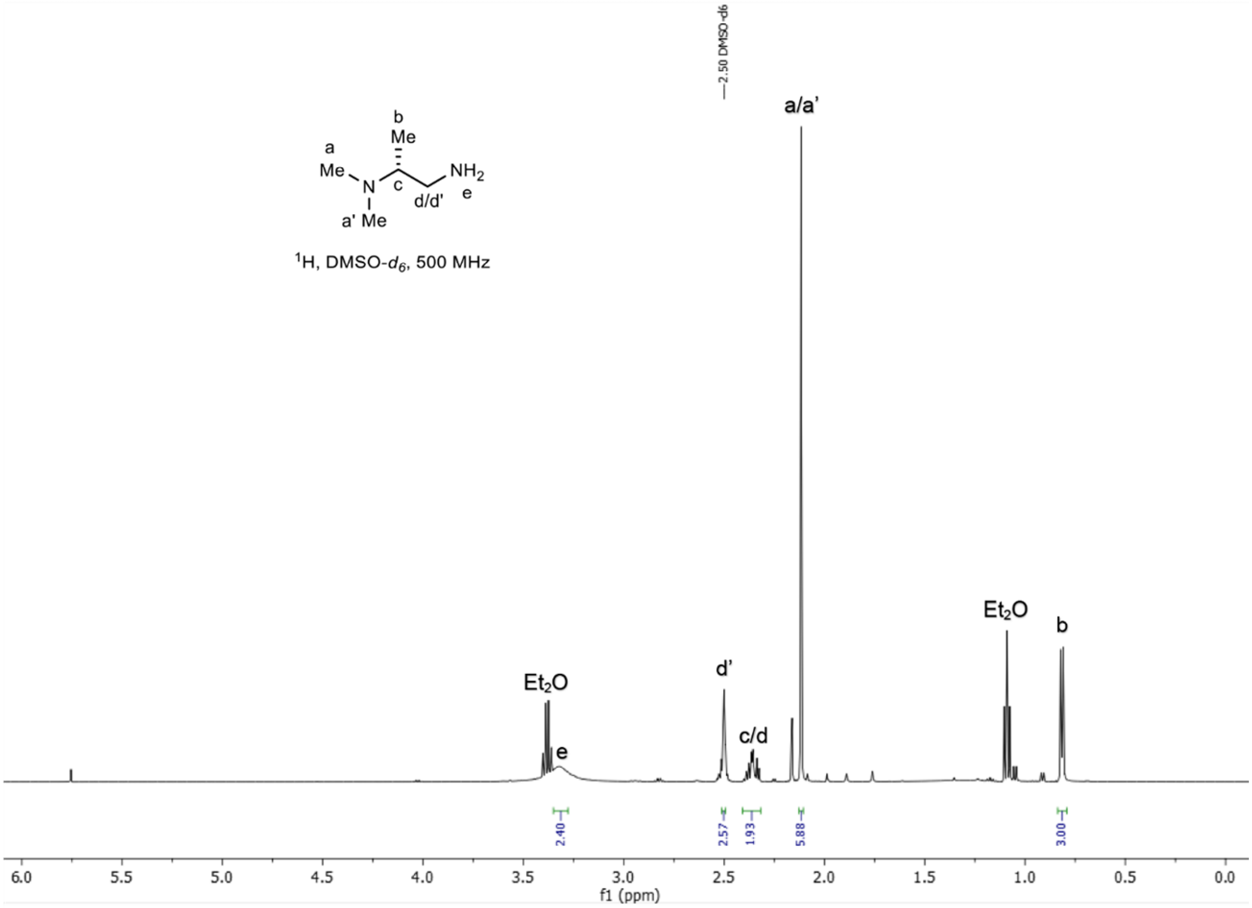

b

(*R*)-Dmp ^{13}C NMR, DMSO-d_6, 126 MHz.

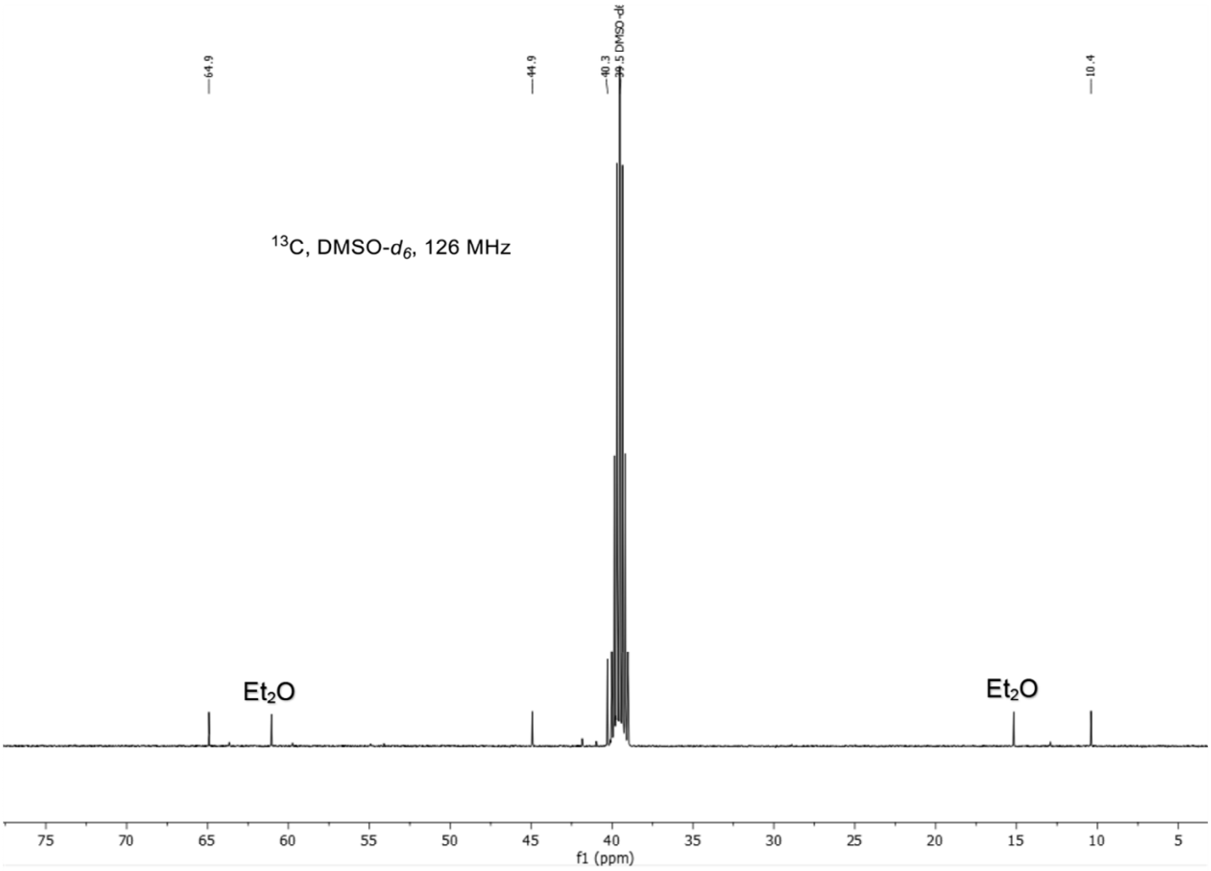

c

(*R*)-Dmp ^1H-^1H COSY NMR, DMSO-d_6, 500 MHz.

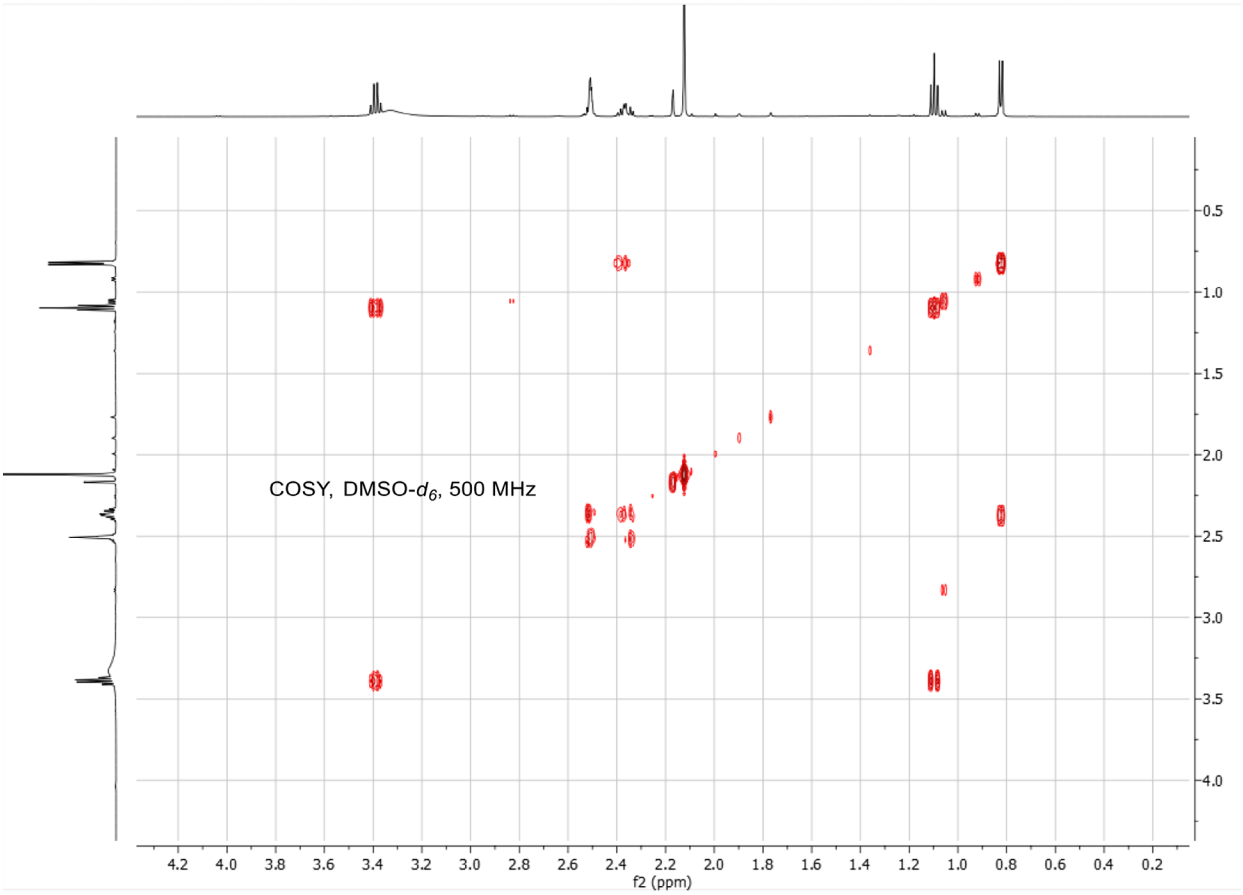

d

(*S*)-Dmp ^{1}H NMR, DMSO-d_6, 500 MHz.

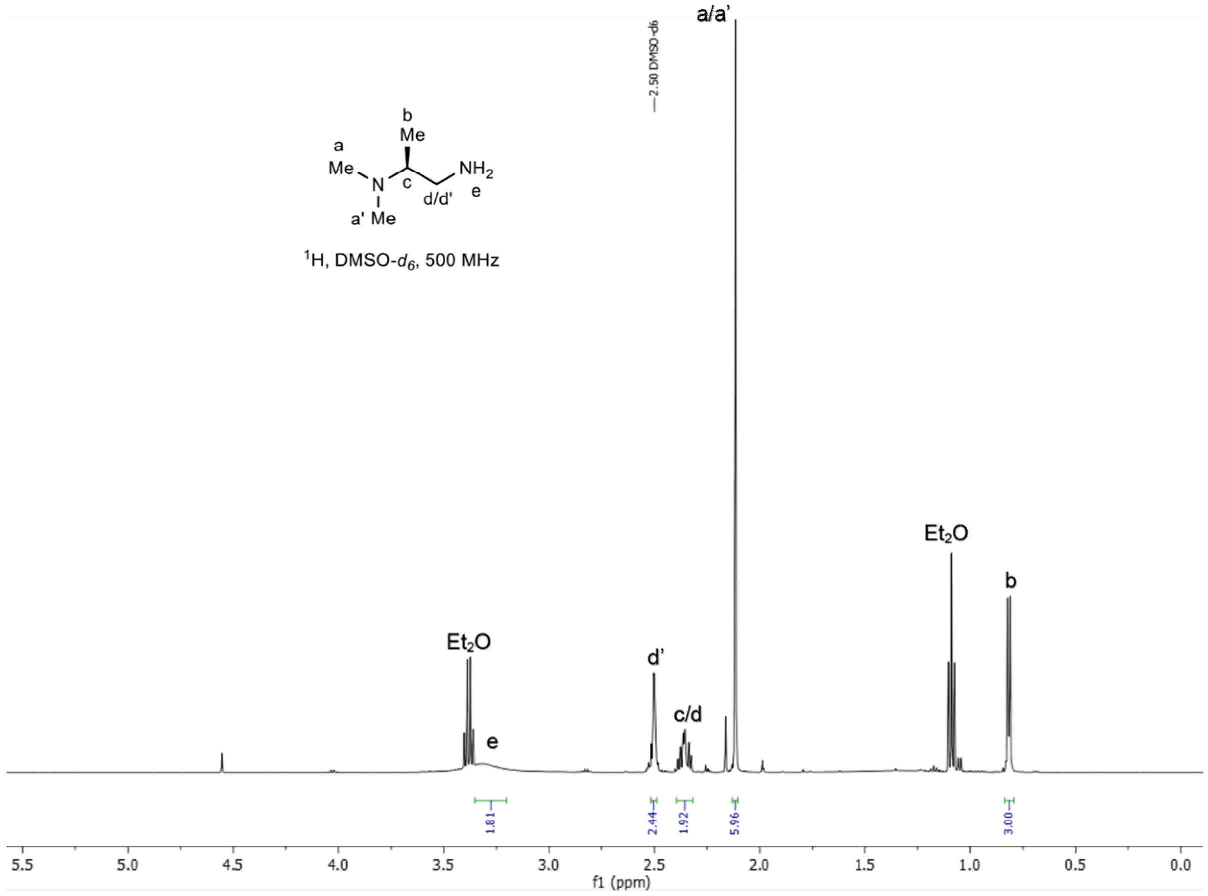

e

(*S*)-Dmp ^{13}C NMR, DMSO-d_6, 126 MHz.

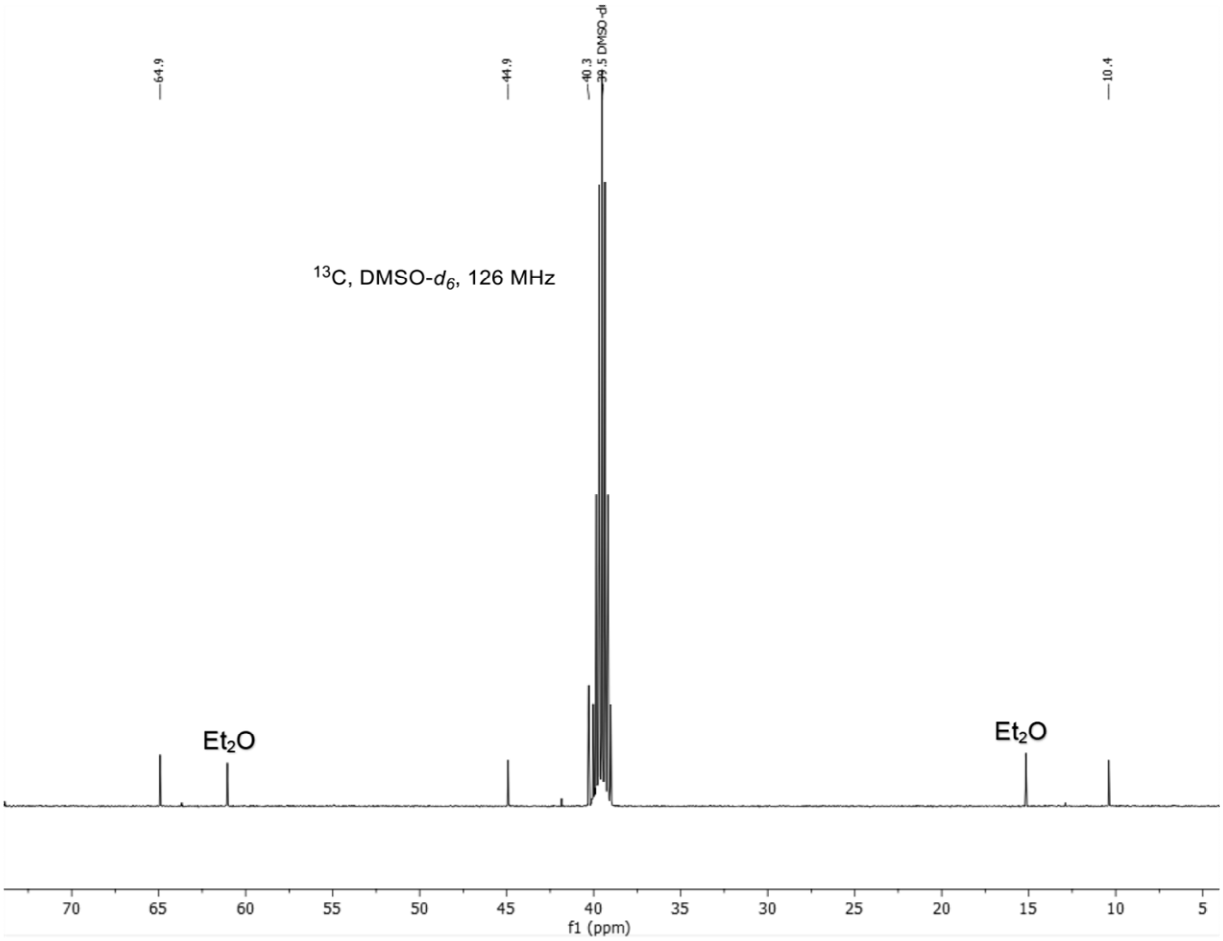

f

(*S*)-Dmp ^{1}H-^{1}H COSY NMR, DMSO-d_6, 500 MHz.

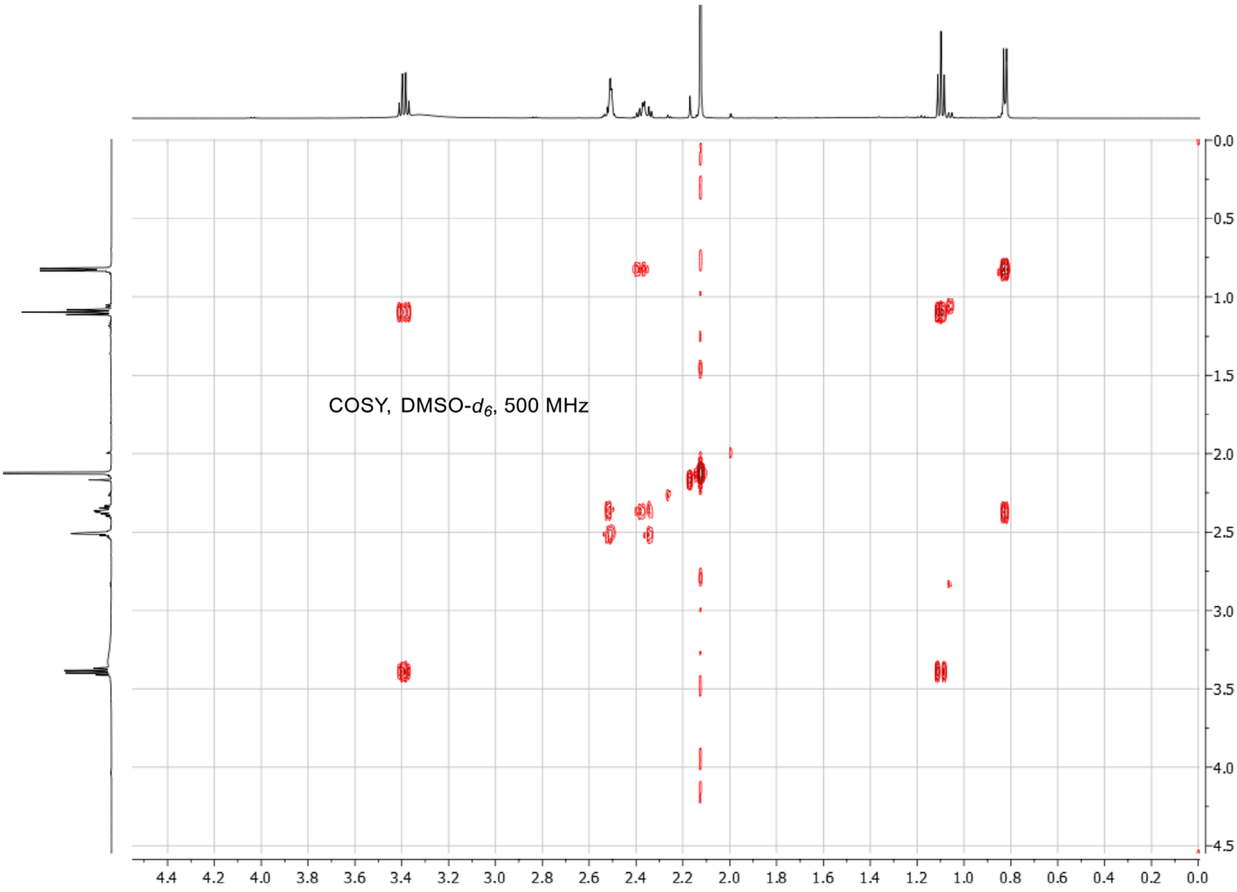

Supplementary Figure 15: NMR spectra of authentic (*R*)-Dmp and (*S*)-Dmp standards. Dmp: N_2,N_2-dimethyl-1,2-propanediamine. (**a**) (*R*)-Dmp ^{1}H NMR, DMSO-d_6, 500 MHz. (**b**) (*R*)-Dmp ^{13}C NMR, DMSO-d_6, 126 MHz. (**c**) (*R*)-Dmp ^{1}H-^{1}H COSY NMR, DMSO-d_6, 500 MHz. (**d**) (*S*)-Dmp ^{1}H NMR, DMSO-d_6, 500 MHz. (**e**) (*S*)-Dmp ^{13}C NMR, DMSO-d_6, 126 MHz. (**f**) (*S*)-Dmp ^{1}H-^{1}H COSY NMR, DMSO-d_6, 500 MHz.

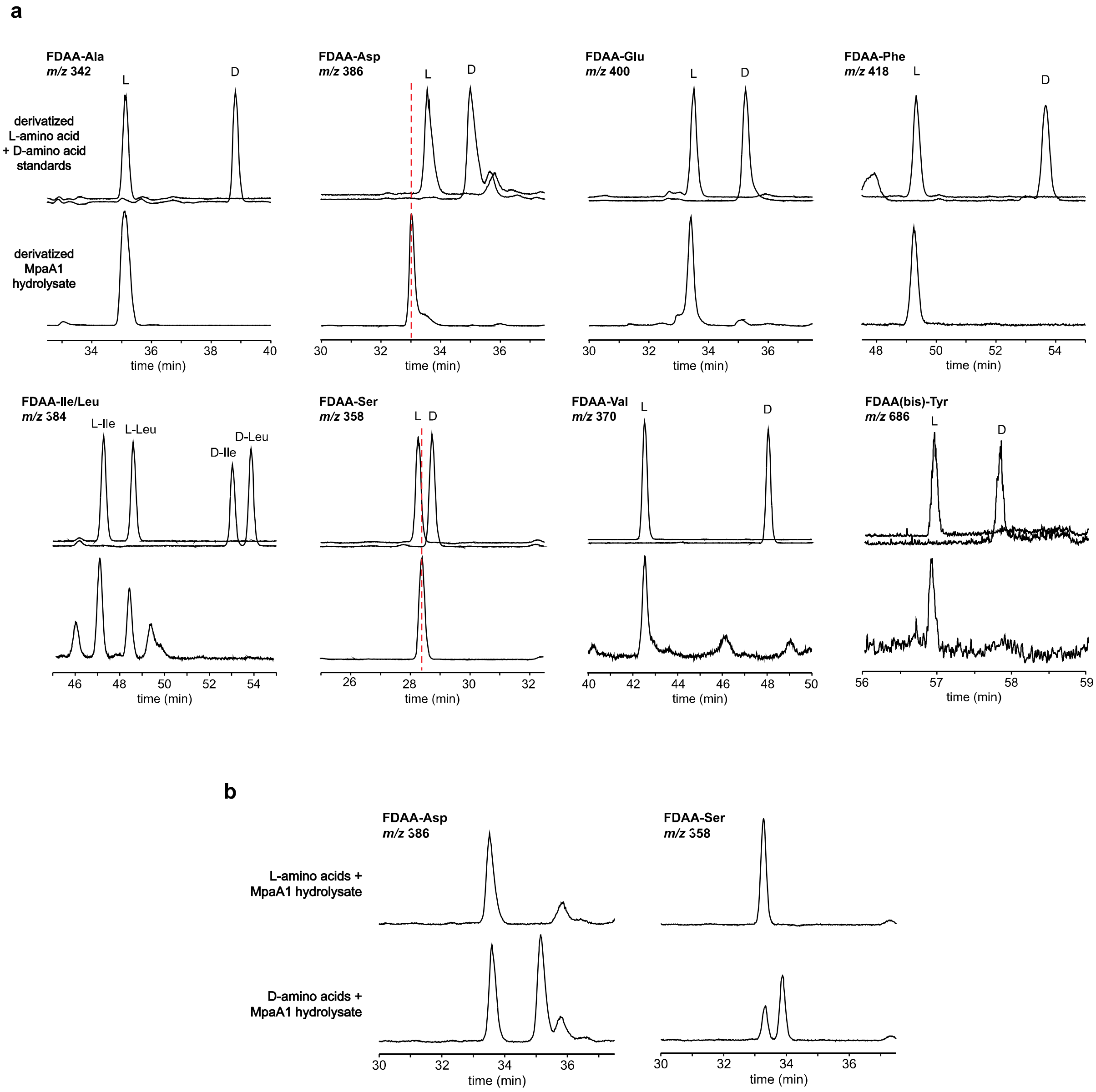

Supplementary Figure 16: Determination of MpaA1 amino acid stereochemistry using LC-MS. (**a**) Ion count-normalized LC-MS chromatograms are shown for derivatized amino acid standards and for 1-fluoro-2-4-dinitrophenyl-5-alanine amide (FDAA)-derivatized hydrolysate of **1**. L- and D-amino acid standards were run individually. FDAA-Trp adducts were not observed[12]. FDAA-Glu was monitored instead of FDAA-Gln due to the hydrolysis of MpaA1. Assignment of Asp and Ser was initially ambiguous owing to retention time drift. (**b**) LC-MS chromatograms for co-injections of derivatized amino acid standards with derivatized MpaA1 hydrolysate.

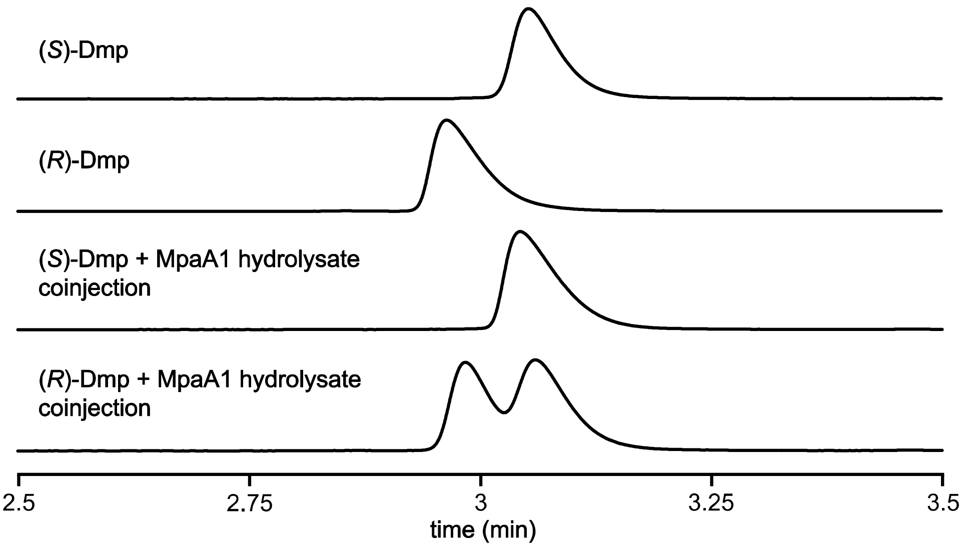

Supplementary Figure 17: Determination of MpaA1 Dmp stereochemistry. Absorbance-normalized chromatograms are shown for single injections of FDAA-derivatized (*R*)-Dmp and (*S*)-Dmp, along with co-injections of derivatized hydrolysate of **1**. Absorbance was monitored at 340 nm. Dmp: N_2,N_2-dimethyl-1,2-propanediamine.

a

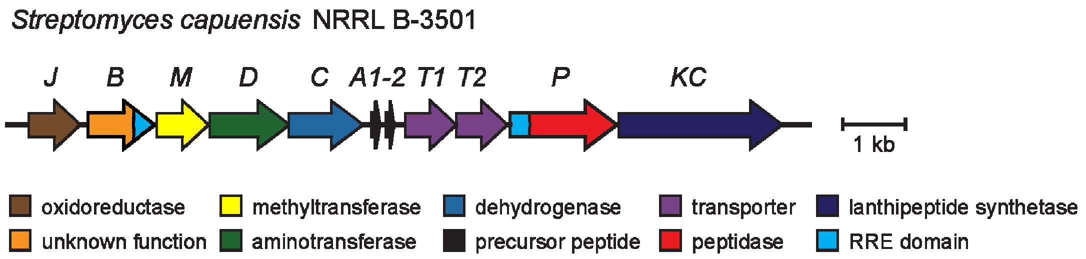

b

```
                Leader                         Core
       -20   -15   -10    -5    1    5    10   15   20   25   30
ScaA1  MQEIEKIQPVLELGMQELEAMDAPGWWTAAGVSAGVVVSASAAYGSAALSAAAIT
ScaA2  MQETEKIQPVLELGMQELEAMEAPGFWTGFSYGVAVSGTAAASAAVSVAASIAT
```

c

Gene	NCBI Accession	Description (per NCBI listing)
scaJ	WP_003980804.1	Luciferase-like monooxygenase (LLM) class flavin-dependent oxidoreductase
scaB	WP_003980803.1	Hypothetical protein
scaM	WP_003980802.1	Class I SAM-dependent methyltransferase
scaD	WP_003980801.1	Aminotransferase class III-fold pyridoxal phosphate-dependent enzyme
scaC	WP_003980800.1	Iron-containing alcohol dehydrogenase
scaA1	WP_165417107.1	Hypothetical protein
scaA2	WP_003980799.1	Hypothetical protein
scaT1	WP_003980797.1	ATP-binding cassette domain-containing protein
scaT2	WP_003980796.1	ABC transporter permease
scaP	WP_231512674.1	Hypothetical protein
scaKC	WP_003980794.1	Class III lanthionine synthetase LanKC

Supplementary Figure 18: The *sca* BGC identified from *Streptomyces capuensis* NRRL B-3501. (**a**) Organization of the *sca* BGC. (**b**) Precursor peptide sequences. (**c**) Functional annotation and accession numbers of genes in the *sca* BGC

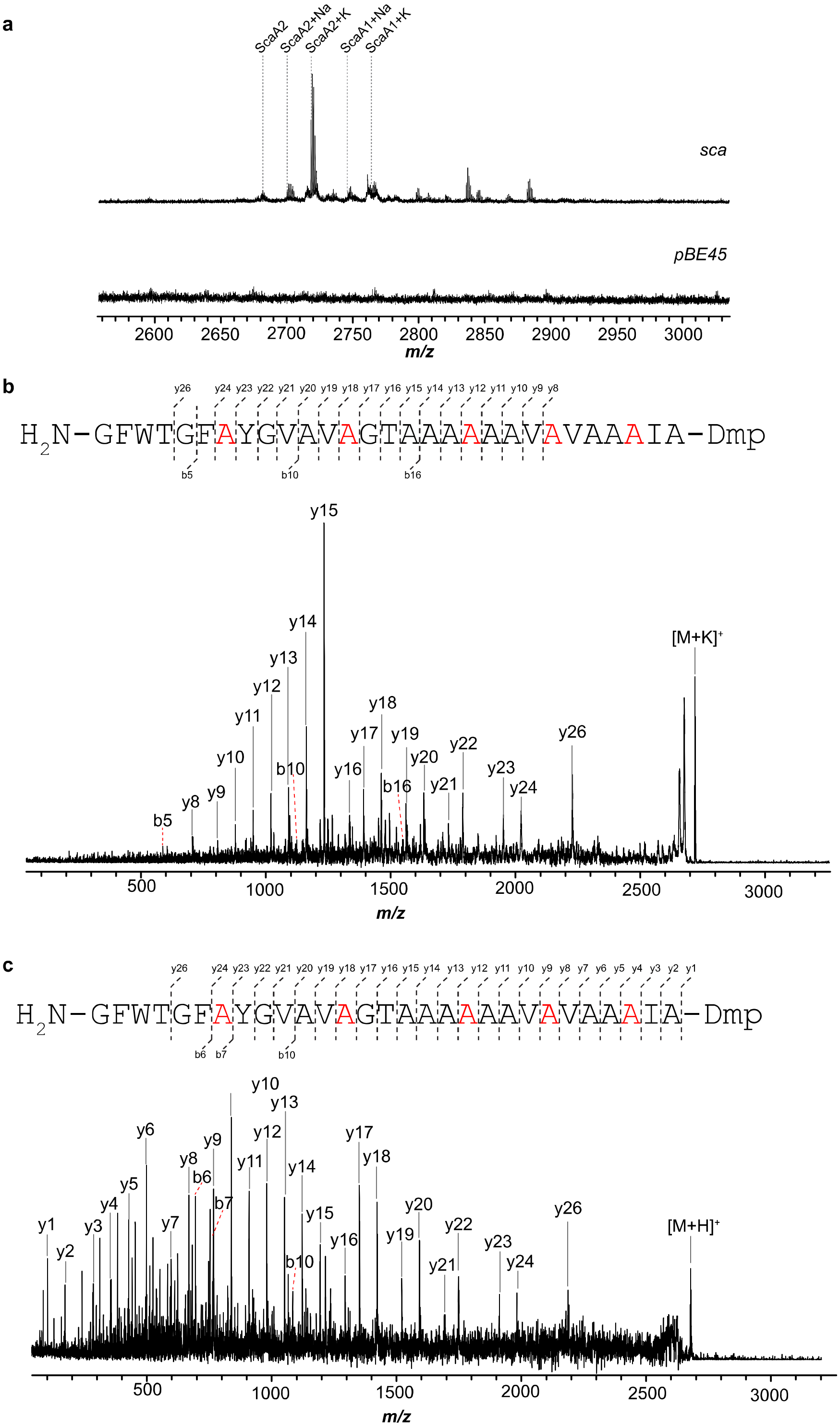

Supplementary Figure 19: Heterologous expression and product characterization of *sca*. (a) MALDI-TOF mass spectra of the methanol extracts of *S. albus* J1074 containing *sca* and the pBE45 empty vector. Sodium and potassium ions are denoted as '+Na' and '+K'. (**b**) MALDI LIFT-TOF/TOF mass spectrum of the modified ScaA2 core peptide acquired by the potassium adduct. Observed b- and y-ions are annotated. Ions are in the +1 charge state. Suspected sites of conversion to *D*-Ala are colored in red. Dmp: N_2,N_2-dimethyl-1,2-propanediamine. (**c**) MALDI LIFT-TOF/TOF mass spectrum of the modified ScaA2 core peptide acquired using the $[M+H]^+$ ion. Observed b- and y-ions are annotated. Ions are in the +1 charge state. Suspected sites of conversion to *D*-Ala are colored in red. Dmp: N_2,N_2-dimethyl-1,2-propanediamine.

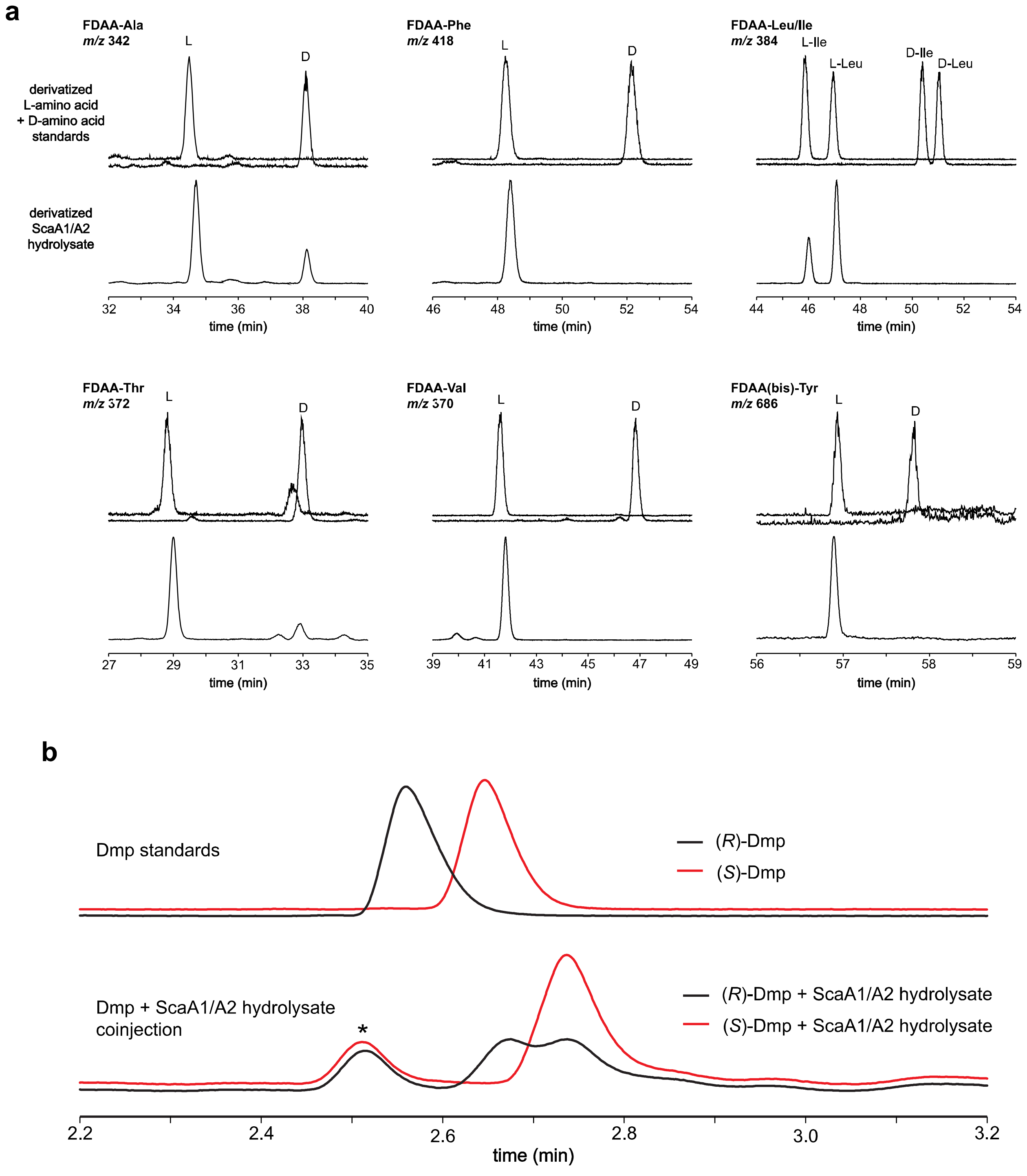

Supplementary Figure 20: Determination of ScaA1/A2 residue stereochemistry. (**a**) Ion count-normalized LC-MS chromatograms are shown for derivatized amino acid standards and for 1-fluoro-2-4-dinitrophenyl-5-alanine amide (FDAA)-derivatized hydrolysate of ScaA1/A2. L- and D-amino acid standards were run individually. FDAA-Trp adducts were not observed[12]. (**b**) Absorbance-normalized chromatograms are shown for single injections of FDAA-derivatized (*R*)-Dmp and (*S*)-Dmp, along with co-injections of derivatized hydrolysate of ScaA1/A2. Absorbance was monitored at 340 nm. Asterisk indicates possible FDAA adduct to unidentified hydrolysis product. Dmp: N_2,N_2-dimethyl-1,2-propanediamine.

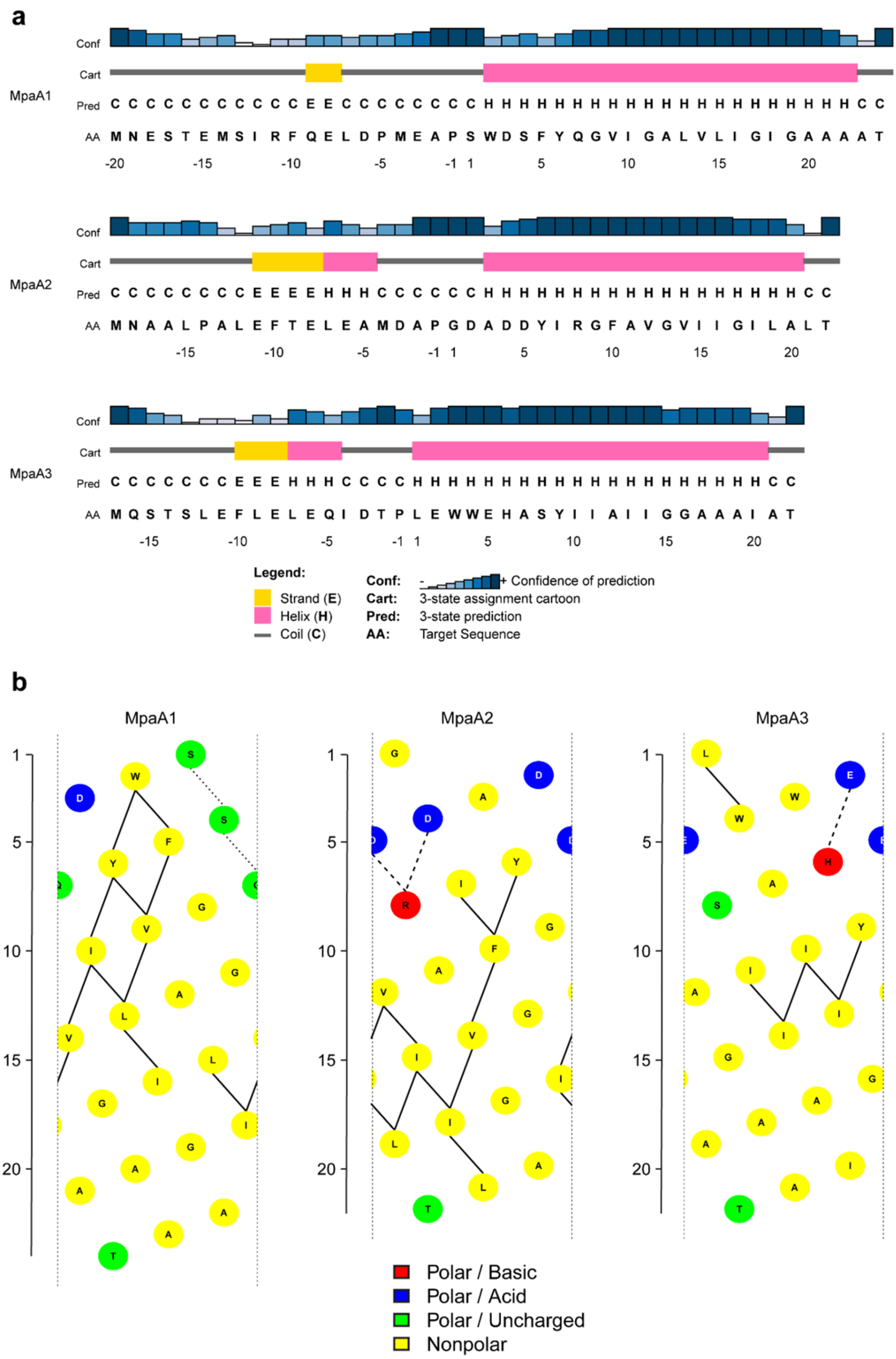
a
MpaA1
Conf
Cart
Pred C C C C C C C C C C C E E C C C C C C C C H H H H H H H H H H H H H H H H H H H H H C C
AA M N E S T E M S I R F Q E L D P M E A P S W D S F Y Q G V I G A L V L I G I G A A A A T
-20 -15 -10 -5 -1 1 5 10 15 20
MpaA2
Conf
Cart
Pred C C C C C C C C E E E E H H H C C C C C C H H H H H H H H H H H H H H H H H H H C C
AA M N A A L P A L E F T E L E A M D A P G D A D D Y I R G F A V G V I I G I L A L T
-15 -10 -5 -1 1 5 10 15 20
MpaA3
Conf
Cart
Pred C C C C C C C E E E H H H C C C C H H H H H H H H H H H H H H H H H H H H C C
AA M Q S T S L E F L E L E Q I D T P L E W W E H A S Y I I A I I G G A A A I A T
-15 -10 -5 -1 1 5 10 15 20
Legend:
Strand (E)
Helix (H)
Coil (C)
Conf: - + Confidence of prediction
Cart: 3-state assignment cartoon
Pred: 3-state prediction
AA: Target Sequence
b
MpaA1
MpaA2
MpaA3
1
5
10
15
20
Polar / Basic
Polar / Acid
Polar / Uncharged
Nonpolar

c

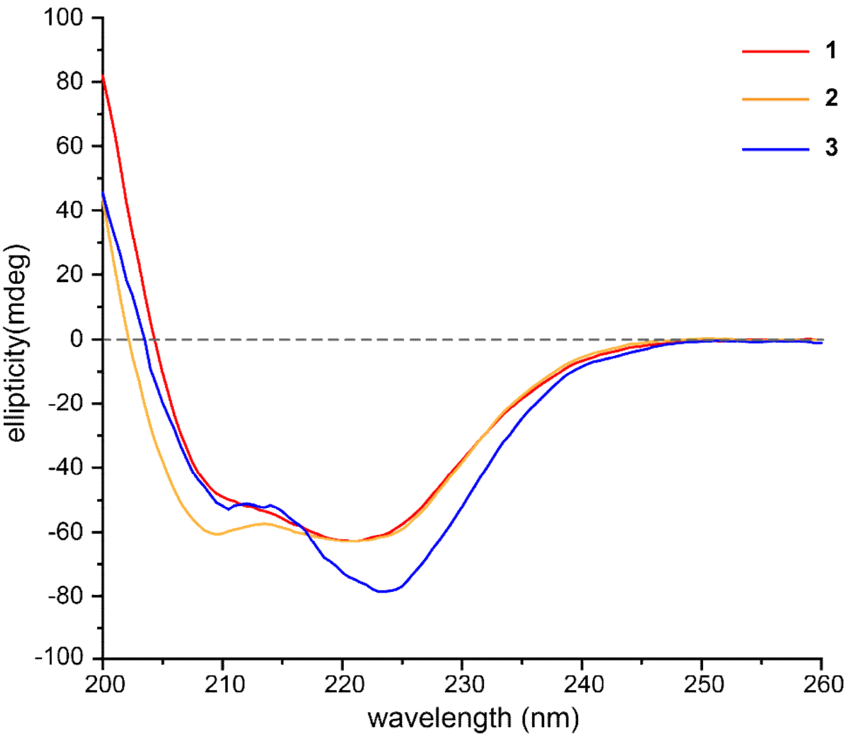

Supplementary Figure 21: Secondary structure of daptides 1-3. (**a**) α-helices of MpaA1-3 predicted by PSI-PRED4.0[13] (http://bioinf.cs.ucl.ac.uk/psipred/). (**b**) Helical net diagrams showing predicted intra-helix interactions for MpaA1-3 core peptides. Diagrams were generated using NetWheels (http://lbqp.unb.br/NetWheels/). (**c**, following page) Circular dichroism spectra of **1**-**3**.

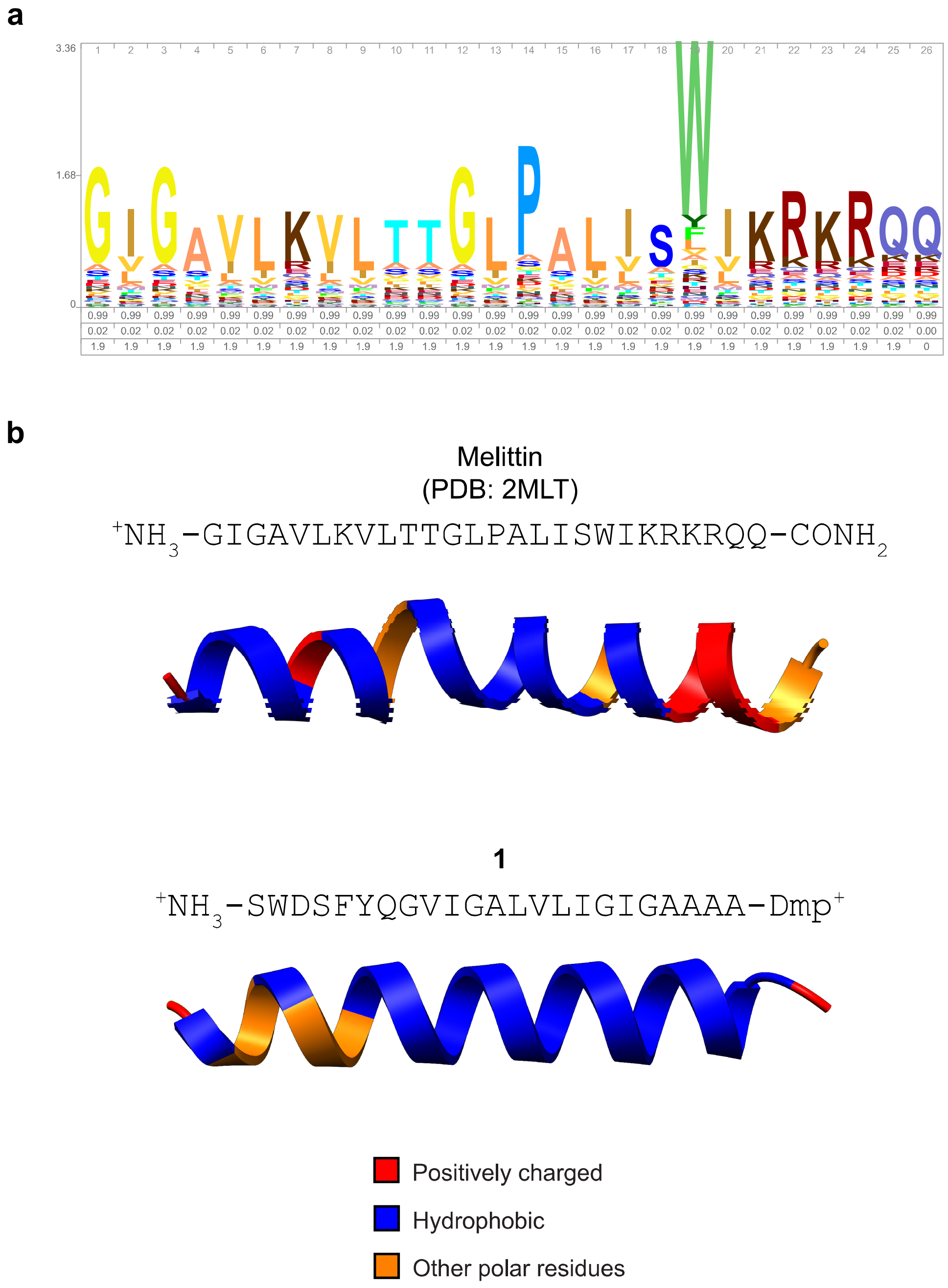

Supplementary Figure 22: Comparison of melittin to daptide 1. (**a**) Sequence logo for the melittin family is shown. The logo was generated using SkyLign[9] on the melittin pHMM (Pfam identifier: PF01372)[5]. (**b**) A crystal structure of melittin and predicted structure of **1** are shown. AlphaFold[14] was used to predict the MpaA1 complete sequence, and only the core region is shown. Melittin structure was re-created based on a review article[15]. Dmp: N_2,N_2-dimethyl-1,2-propanediamine.

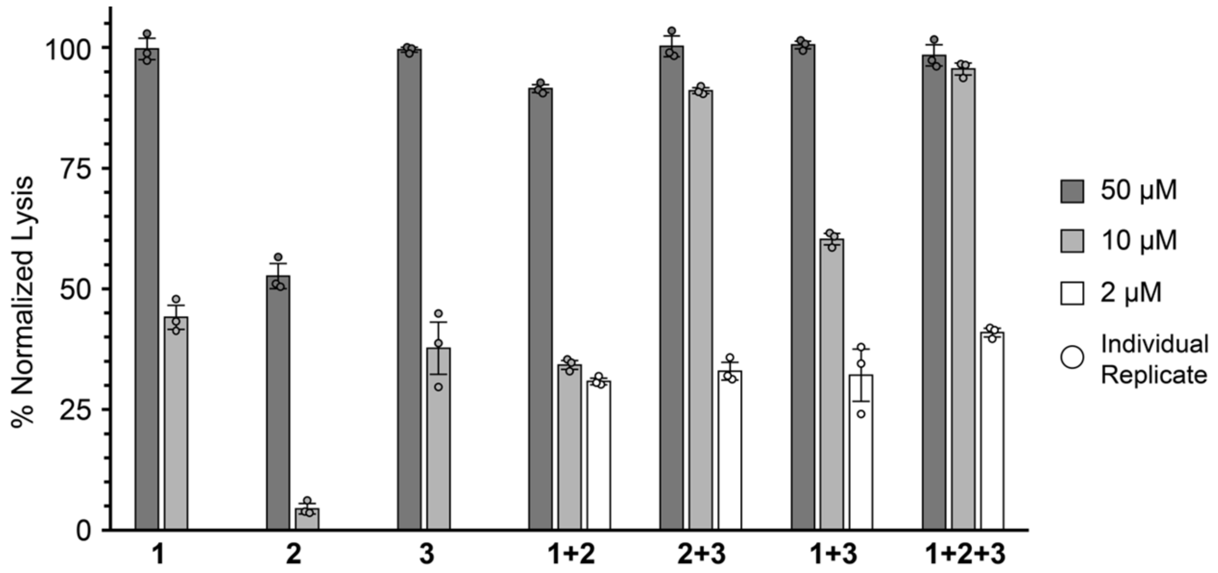

Supplementary Figure 23: Hemolytic activity of daptides 1-3. Hemolysis of bovine erythrocytes was normalized to the positive control [5% (*v/v*) Triton X-100]. All individual and combination daptide treatments were assessed. The percentage of hemolysis at total concentrations of 50 µM and 10 µM are shown as grey and white bars, respectively. In the combination treatments, the total daptide concentration is listed (i.e., each daptide contributes 1/2 or 1/3 of the total concentration). All data represent the mean of n = 3 biologically independent samples and error bars show standard deviation.

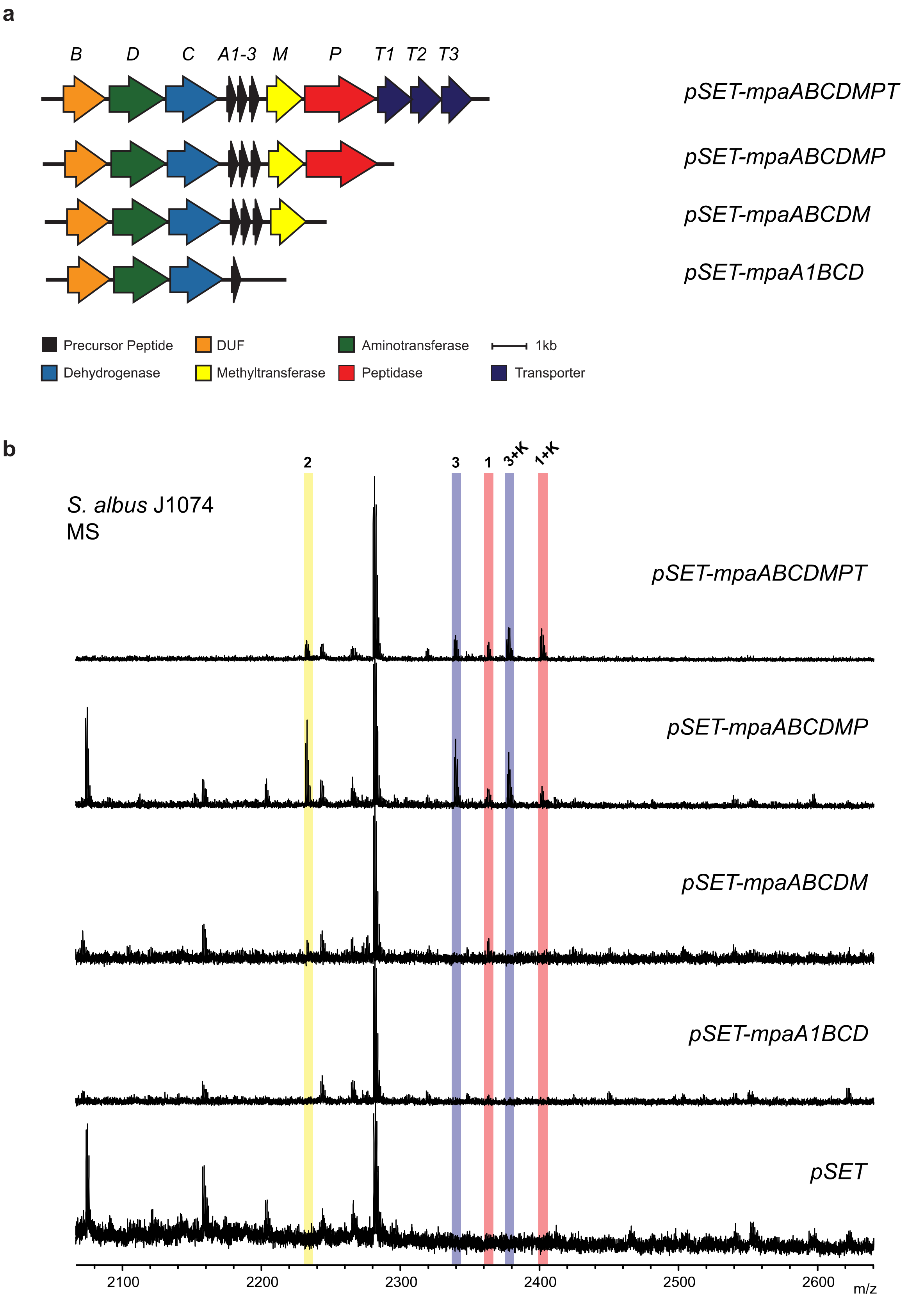

Supplementary Figure 24: Heterologous expression and *mpa* gene omissions in *S. albus* J1074. (**a**) Gene organization of the minimal *mpa* BGC (no flanking genes included) with examined gene omissions depicted. (**b**) MALDI-TOF mass spectra of the corresponding methanol extracts.

a

Condition	Medium	Induction Density OD_{600}	Expression Time (h)	Expression Temperature (°C)
1	TB	1.0	20	18
2	TB	1.0	3	18
3	TB	1.5	3	37
4	LB	0.6	20	18
5	M9	0.7	36	18

b

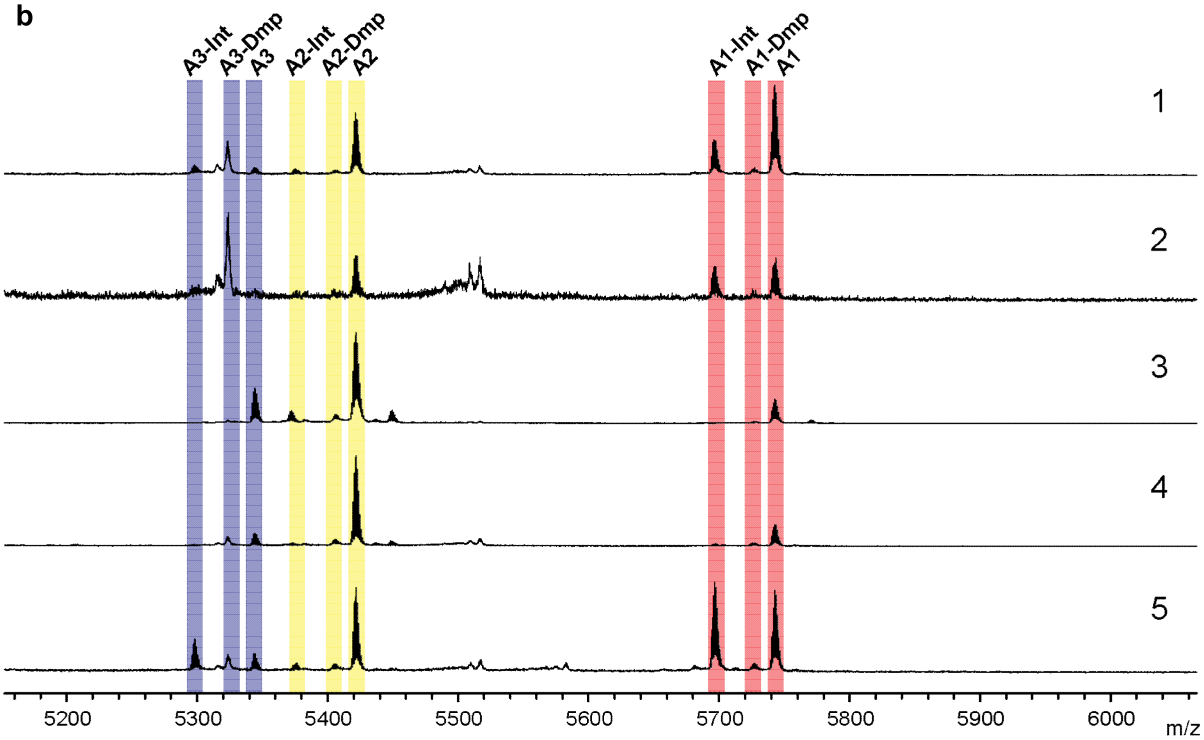

Supplementary Figure 25: Expression of the refactored *mpaABCDE* under various cultivation conditions. (**a**) List of variables for expression conditions 1-5, which used IPTG (0.5 mM final) for induction . (**b**) MALDI-TOF mass spectra of IMAC-purified products from expression conditions 1-5. The ketone/amine intermediates are denoted by "-Int" while the fully modified products are denoted by "-Dmp". Dmp: N_2,N_2-dimethyl-1,2-propanediamine

a

Plasmid	Chaperone Genes	Promoter	Inducer
pTf16	*tig*	araB	L-Arabinose: 0.5 mg/ml
pG-KJE8	*dnaK-dnaJ-grpE*<br>*gro ES-groEL*	araB<br>Pzt-1	L-Arabinose: 0.5 mg/ml<br>Tetracycline: 1 ng/ml
pKJE7	*dnaK-dnaJ-grpE*	araB	L-Arabinose: 0.5 mg/ml
pGro7	*groES-groEL*	araB	L-Arabinose: 0.5 mg/ml
pG-Tf2	*groES-groEL-tig*	Pzt-1	Tetracycline: 1 ng/ml

b

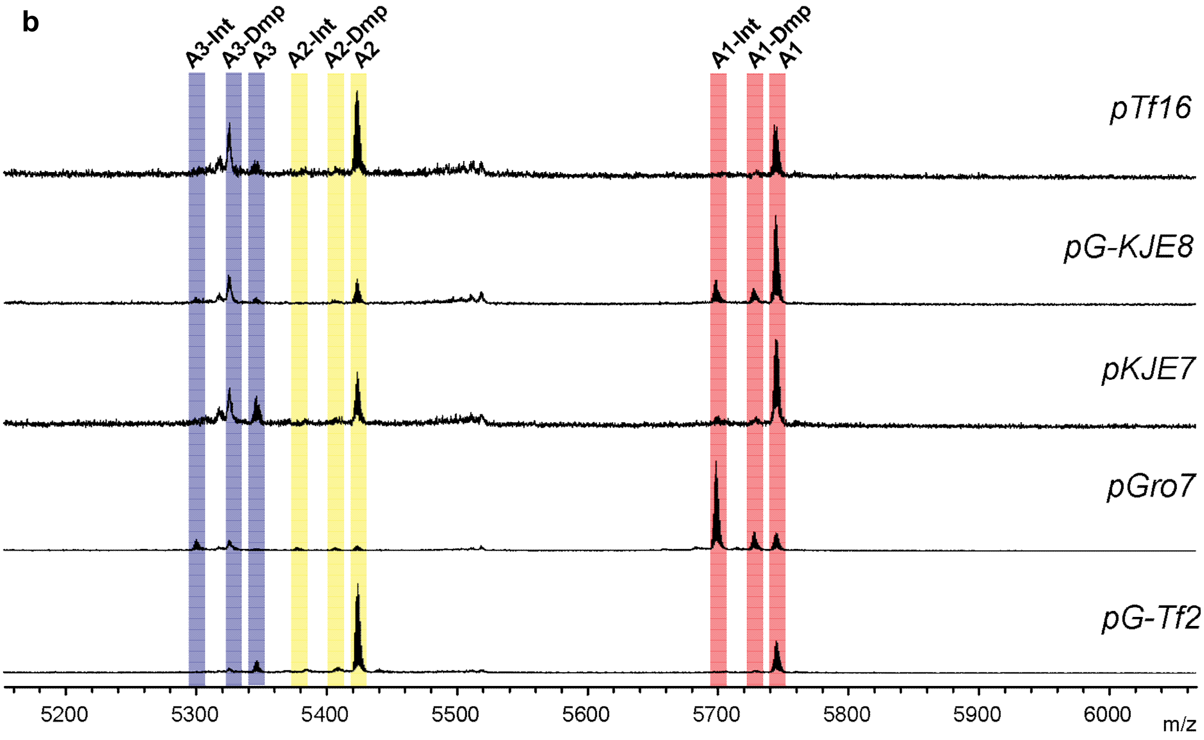

Supplementary Figure 26: Expression of the refactored *mpaABCDE* with chaperone plasmids. (**a**) Information for the genes involved in each plasmid. All chaperone plasmids were obtained from TaKaRa Bio Inc. Additional expression parameters were: medium, TB; induction OD_{600}, 0.8; [IPTG] 0.1 mM; expression temperature, 18 °C; expression time, 18 h. (**b**) MALDI-TOF mass spectra of IMAC purified products. The ketone/amine intermediates are denoted by "-Int" while the fully modified products are denoted by "-Dmp". Dmp: N_2,N_2-dimethyl-1,2-propanediamine.

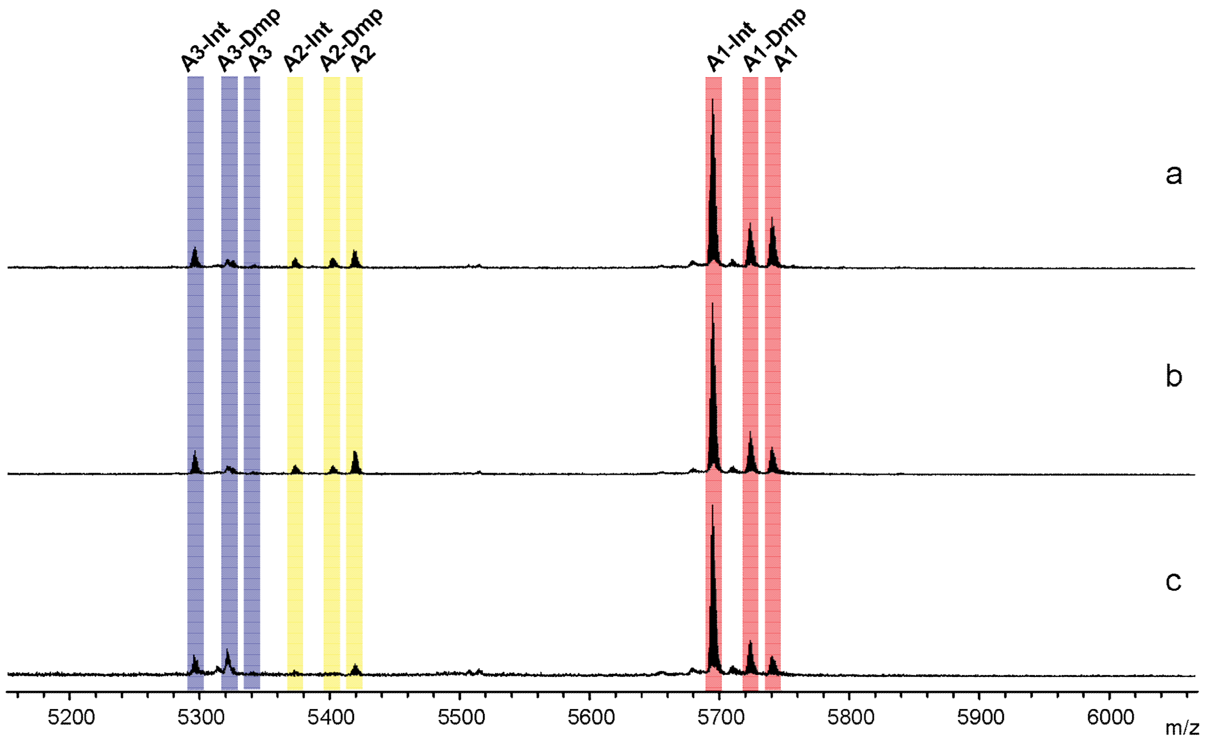

Supplementary Figure 27: Expression of the refactored *mpaABCDE* with pGro7 chaperone plasmid under various arabinose concentrations (a = 0.5 mg/mL, b = 2.0 mg/mL, c = 4.0 mg/mL). Additional expression parameters were: medium, TB; induction OD_{600}, 0.8; [IPTG], 0.1 mM; expression temperature, 18 °C; expression time, 18 h. The ketone/amine intermediates are denoted by "-Int" while the fully modified products are denoted by "-Dmp". Dmp: N_2,N_2-dimethyl-1,2-propanediamine.

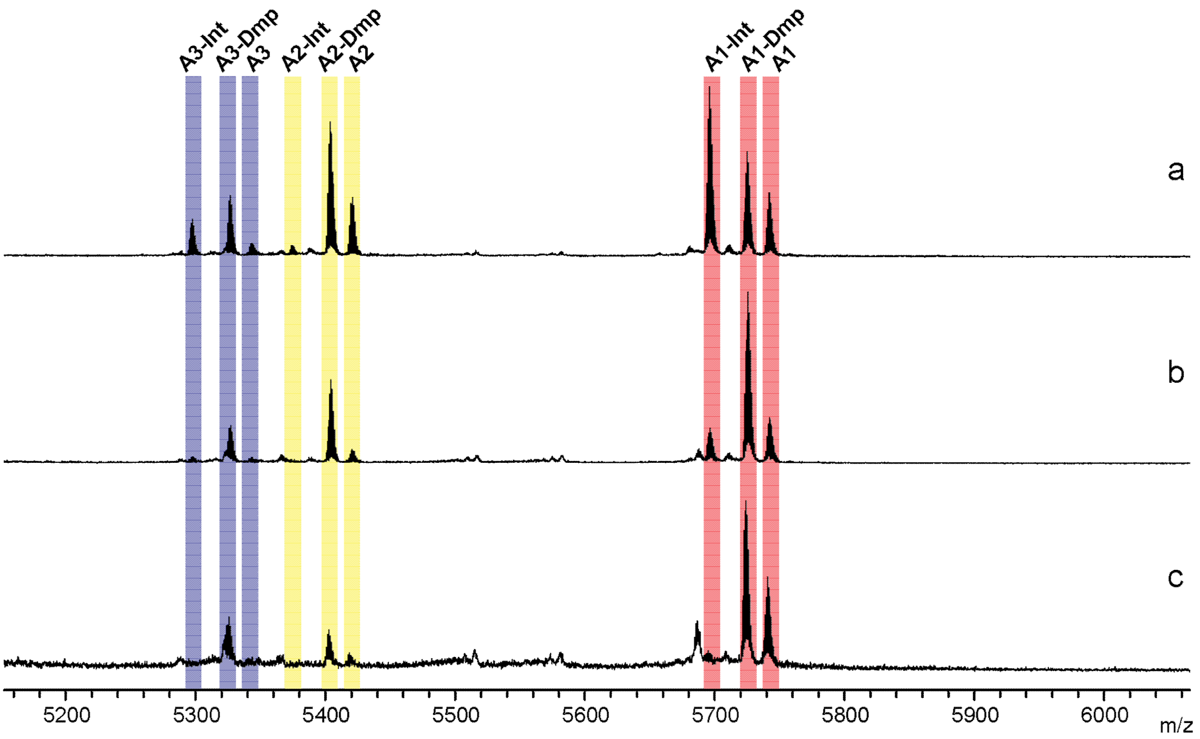

Supplementary Figure 28: Expression of the refactored *mpaABCDE* with pGro7 chaperone plasmid with extended expression time (a = 36 h; b = 72 h; c = 96 h). Additional expression parameters were: medium, M9; [arabinose], 0.5 mg/ml; induction OD_{600}, 0.7; [IPTG], 0.5 mM; expression temperature, 18 °C. The ketone/amine intermediates are denoted by "-Int" while the fully modified products are denoted by "-Dmp". Dmp: N_2,N_2-dimethyl-1,2-propanediamine.

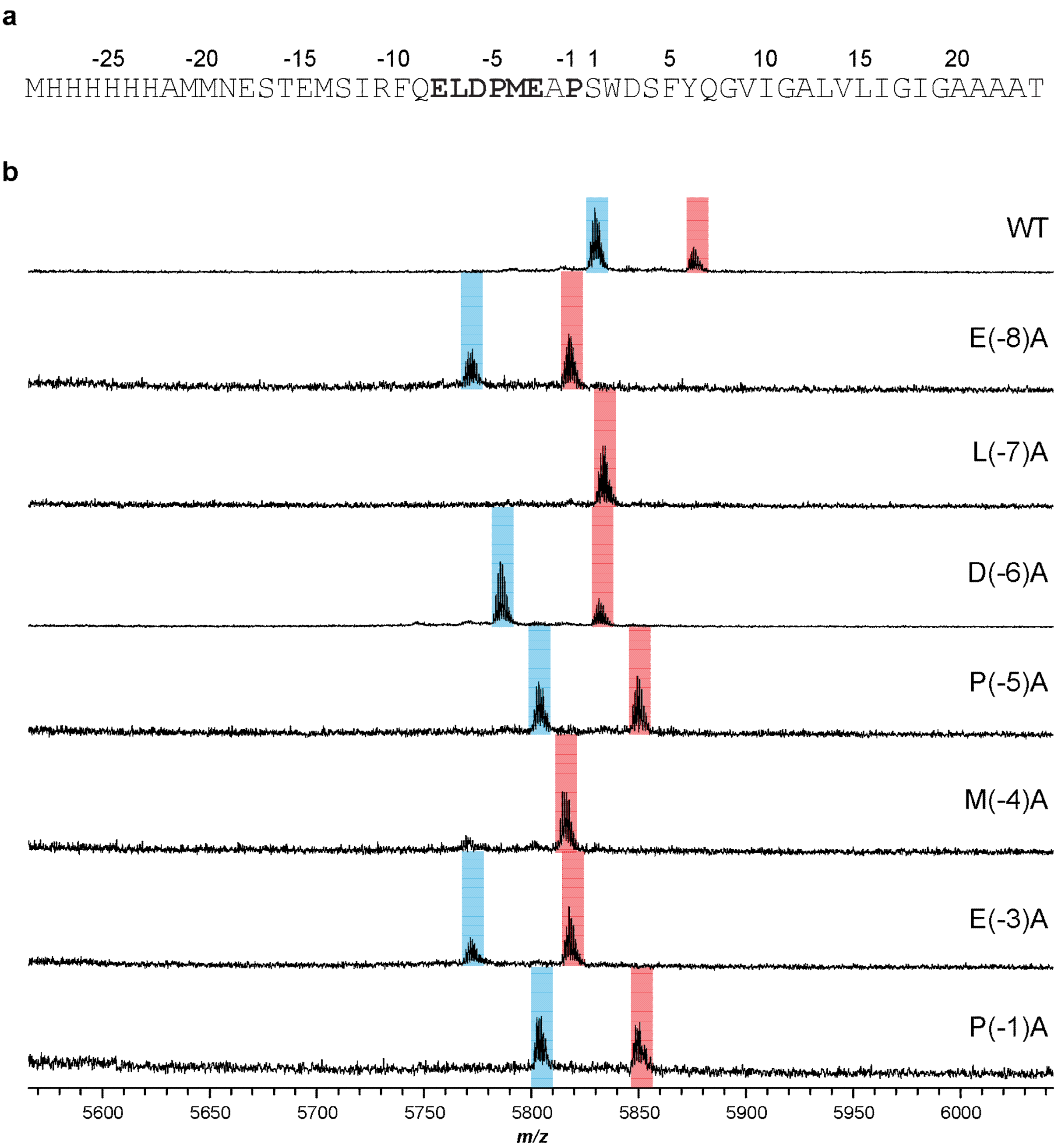

Supplementary Figure 29: MALDI-TOF-MS analysis of MpaA1 leader region variants co-expressed with MpaB and MpaC. (**a**) Sequence of MpaA1 with varied residues shown in bold. (**b**) MALDI-TOF mass spectra of IMAC-purified products after co-expression under the following condition: medium, TB; chaperone plasmid, pGro7; [arabinose], 0.5 mg/mL; induction OD_{600}, 0.8; [IPTG], 0.1 mM; expression temperature, 18 °C; expression time, 18 h. Ions for unmodified and modified peptides are highlighted in red and blue, respectively.

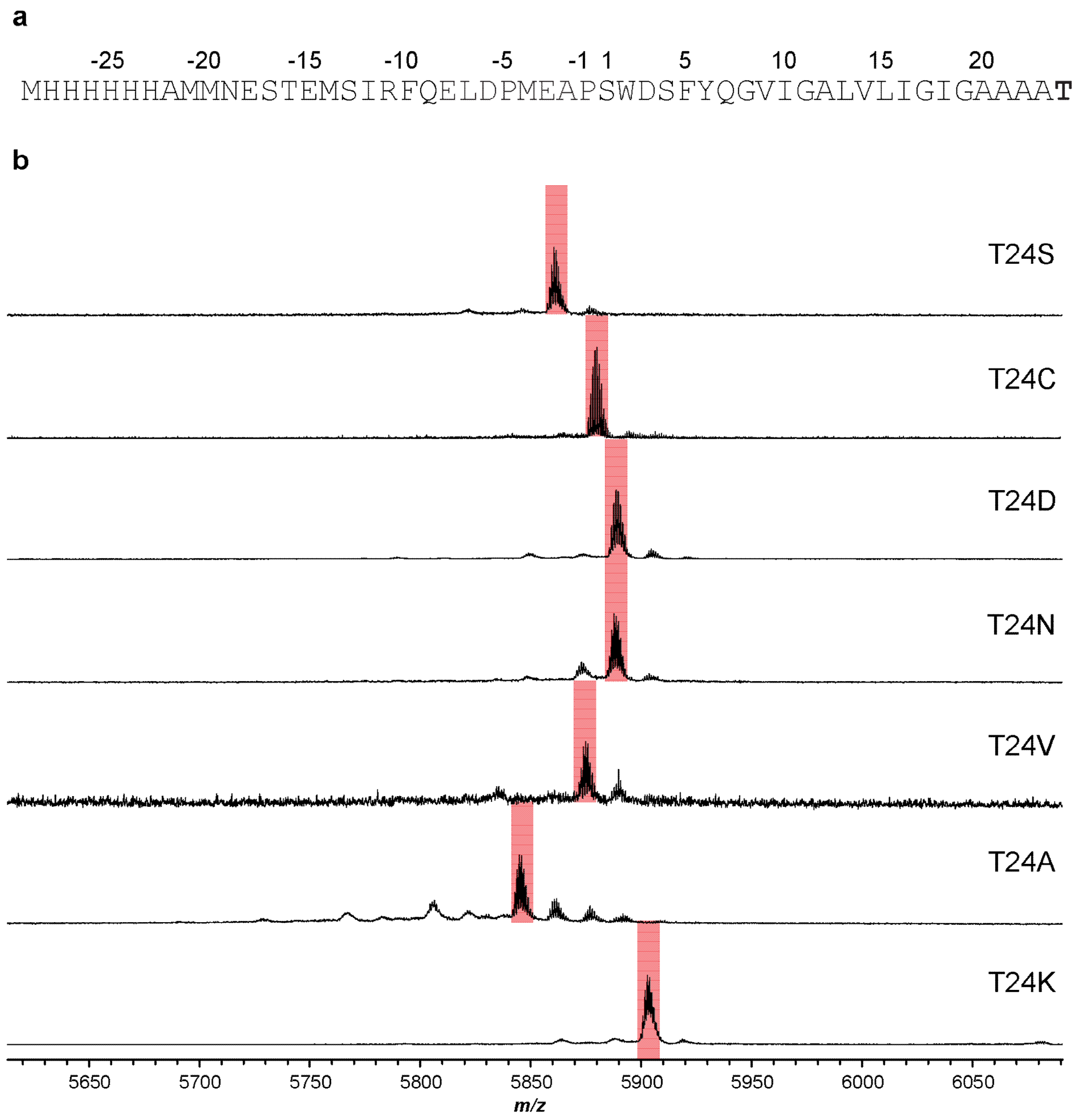

Supplementary Figure 30: MALDI-TOF-MS analysis of MpaA1 *C*-terminal Thr variants that were co-expressed with MpaB and MpaC. (**a**) Sequence of MpaA1 with the replaced C-terminal Thr shown in bold. (**b**) MALDI-TOF mass spectra of IMAC-purified products after co-expression under the following condition: medium, M9; chaperone plasmid, pGro7; [arabinose], 0.5 mg/mL; induction OD_{600}, 0.7; [IPTG], 0.5 mM; expression temperature, 18 °C; expression time, 72 h. Ions for unmodified peptides are highlighted in red. Minor peaks appearing at +16 are presumed to be from Met oxidation.

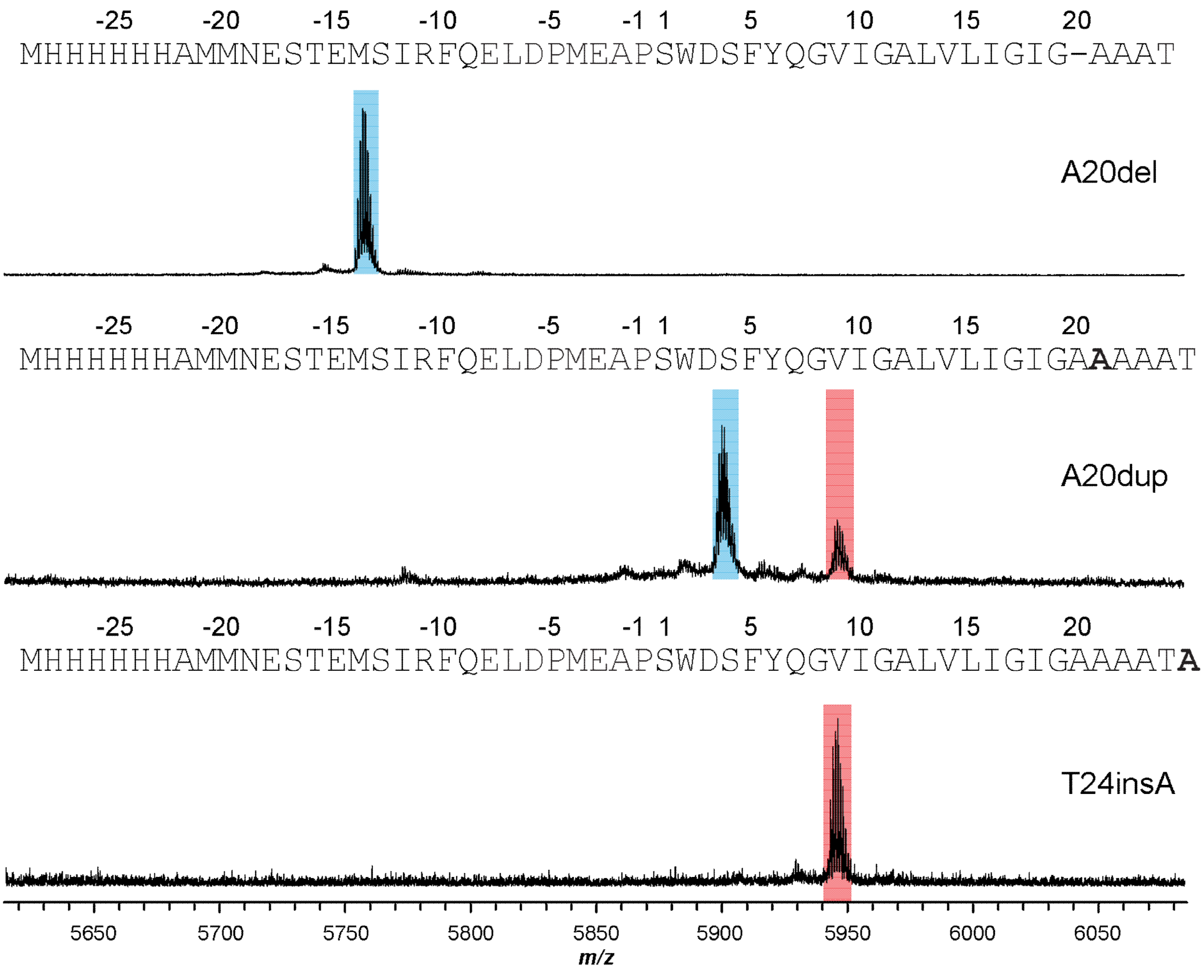

Supplementary Figure 31: MALDI-TOF-MS analysis of MpaA1 insertion and deletion variants at the C-terminus that were co-expressed with MpaB and MpaC. Products were purified by IMAC after co-expression under the following condition: medium, M9; chaperone plasmid, pGro7; [arabinose], 0.5 mg/mL; induction OD_{600}, 0.7; [IPTG], 0.5 mM; expression temperature, 18 °C; expression time, 72 h. Sequences of the MpaA1 variants are shown above their corresponding mass spectra, with the inserted/deleted residues denoted in bold and dash respectively. Ions for unmodified and modified peptides are highlighted in red and blue, respectively.

a

-25 -20 -15 -10 -5 -1 1 5 10 15 20

MHHHHHHAMMNESTEMSIRFQELDPMEAPSWDSFYQ**G**VI**G**AL**V**LI**G**I**G**AAAAT

b

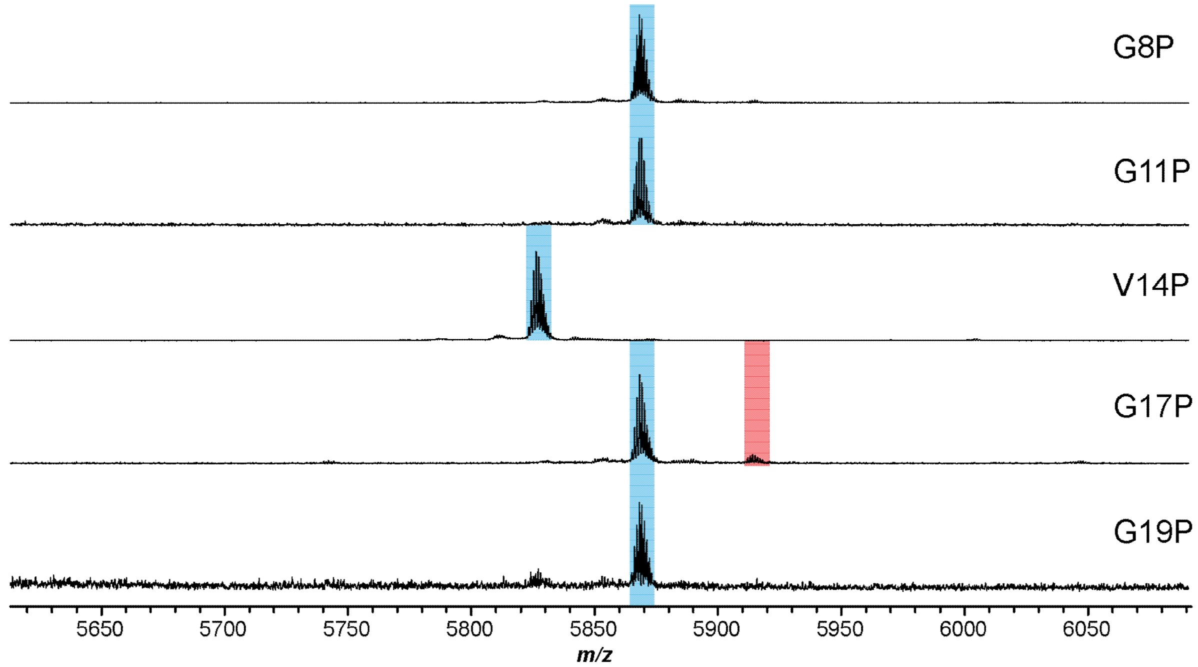

Supplementary Figure 32: MALDI-TOF-MS analysis of MpaA1 variants in the core peptide region that were co-expressed with MpaB and MpaC. (**a**) Sequence of MpaA1 with the mutated residues shown in bold. (**b**) MALDI-TOF mass spectra of IMAC-purified products after co-expression under the following condition: medium, M9; chaperone plasmid, pGro7; [arabinose], 0.5 mg/mL; induction OD_{600}, 0.7; [IPTG], 0.5 mM; expression temperature, 18 °C; expression time, 72 h. Ions for unmodified and modified peptides are highlighted in red and blue, respectively.

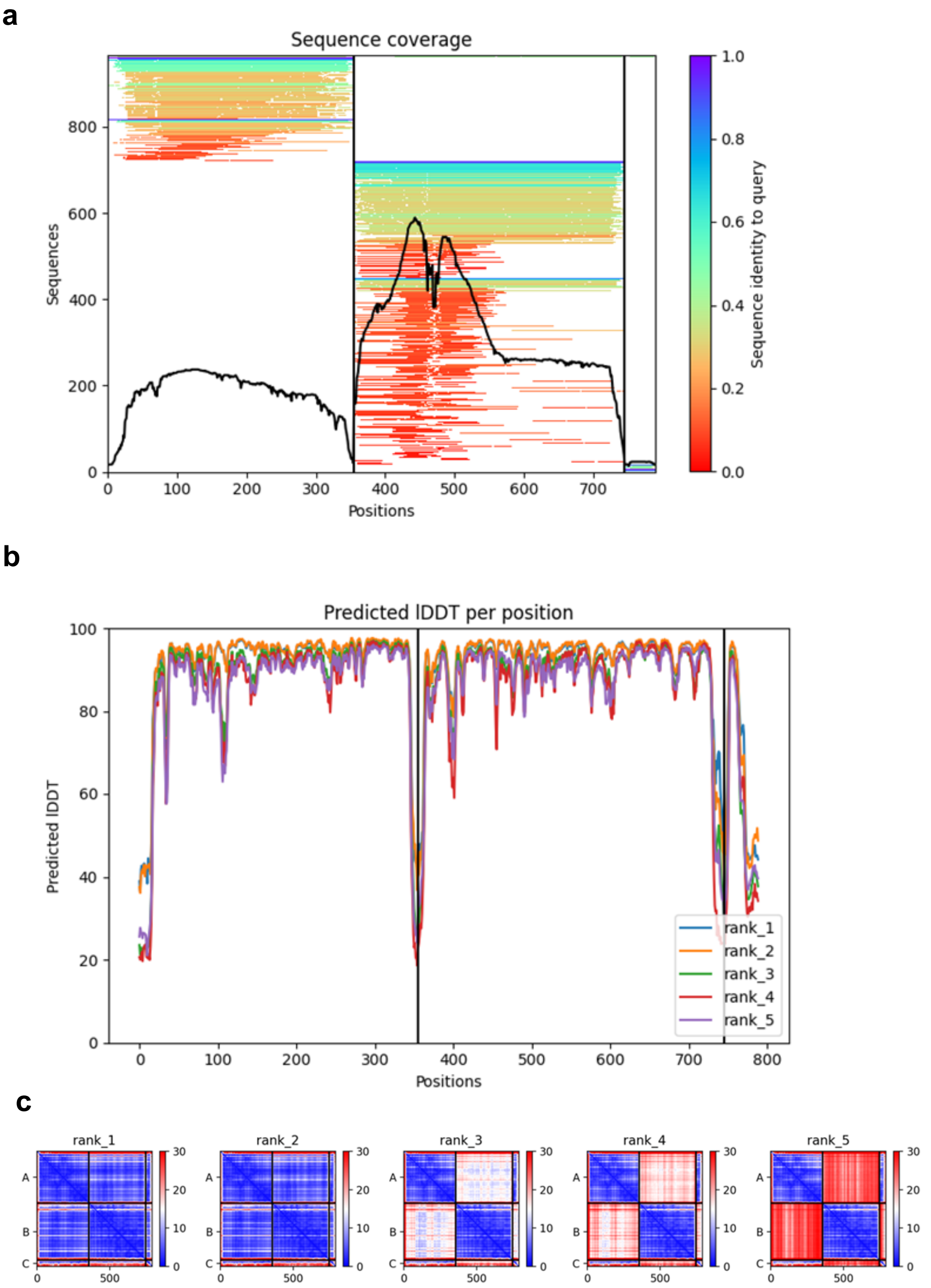

Supplementary Figure 33: Assessment of the AlphaFold-Multimer prediction for the MpaB-MpaC-MpaA1 complex. (**a**) The multiple sequence alignment (MSA) depth during MpaB-MpaC-MpaA1 complex prediction using AlphaFold- Multimer. (**b**) The predicted Local Distance Difference Test (pLDDT) for five predicted models. pLDDT is a per-residue measure of local confidence on a scale from 0-100. (**c**) Predicted Aligned Error (PAE) for five predicted models. The inter PAE between chains is low, indicating a confident prediction.

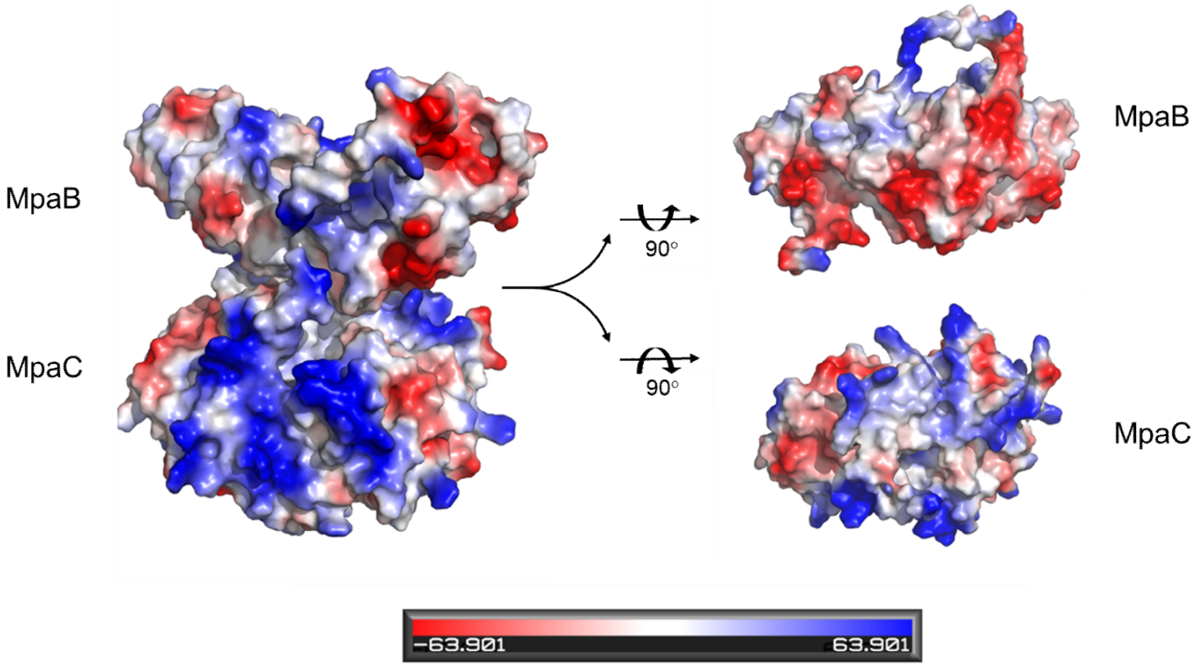

Supplementary Figure 34: Electrostatic surface potential of MpaB and MpaC. MpaB (pI = 4.8) and MpaC (pI = 11.2) are shown as a predicted protein complex on the left with positively and negatively charged surface shown in blue and red, respectively. Views of the interaction surfaces are shown on the right by a 90-degree rotation for each protein. The interaction surface is overall negatively charged for MpaB and positive for MpaC. The electrostatic surface potential is calculated by APBS in PyMOL[16, 17].

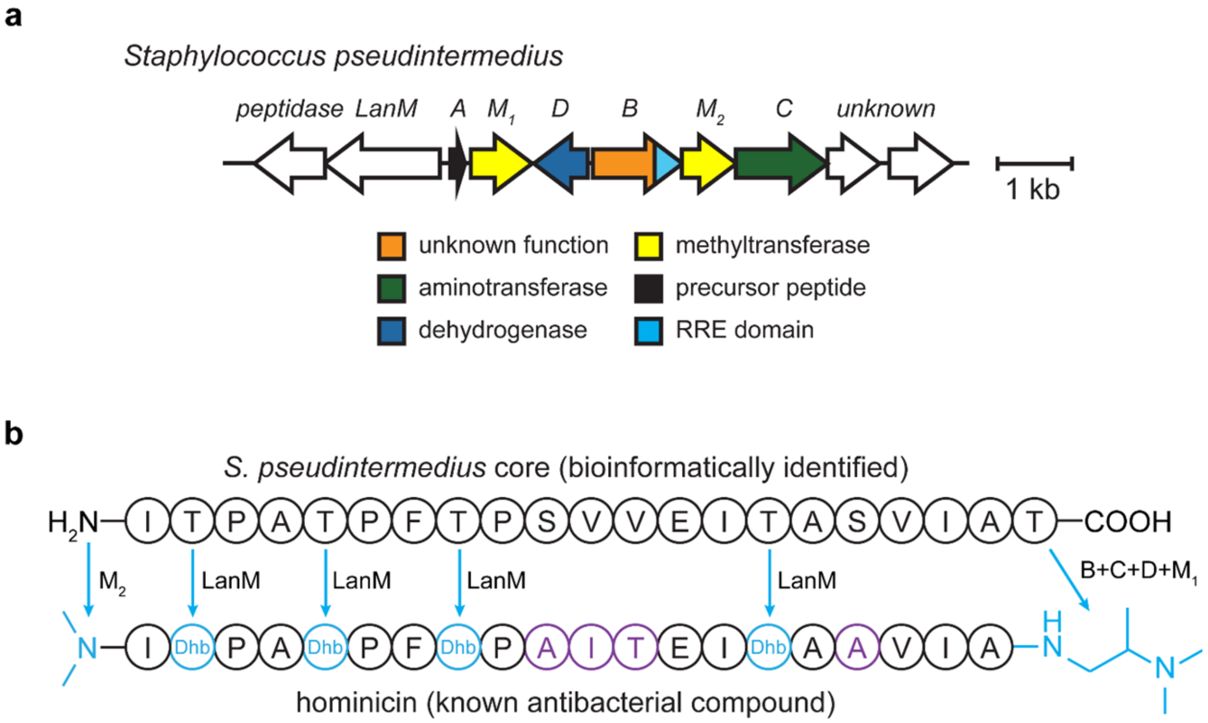

Supplementary Figure 35: Identification of a hominicin-like BGC from *Staphylococcus pseudintermedius*. (**a**) The BGC from *S. pseudintermedius* (DapC protein: WP_214539449.1) is shown. Additionally encoded are a LanM which is proposed to convert the four internal Thr residues to dehydrobutyrine (Dhb), a second methyltransferase (putatively dimethylating the *N*-terminus), a peptidase, and two genes of unknown function. (**b**) Comparison of the core peptide region from the *S. pseudintermedius* BGC and the structure of hominicin[8]. Sites modified in hominicin are cyan and the predicted modifying enzymes are listed. Amino acid variations from the *S. pseudintermedius* core are purple.

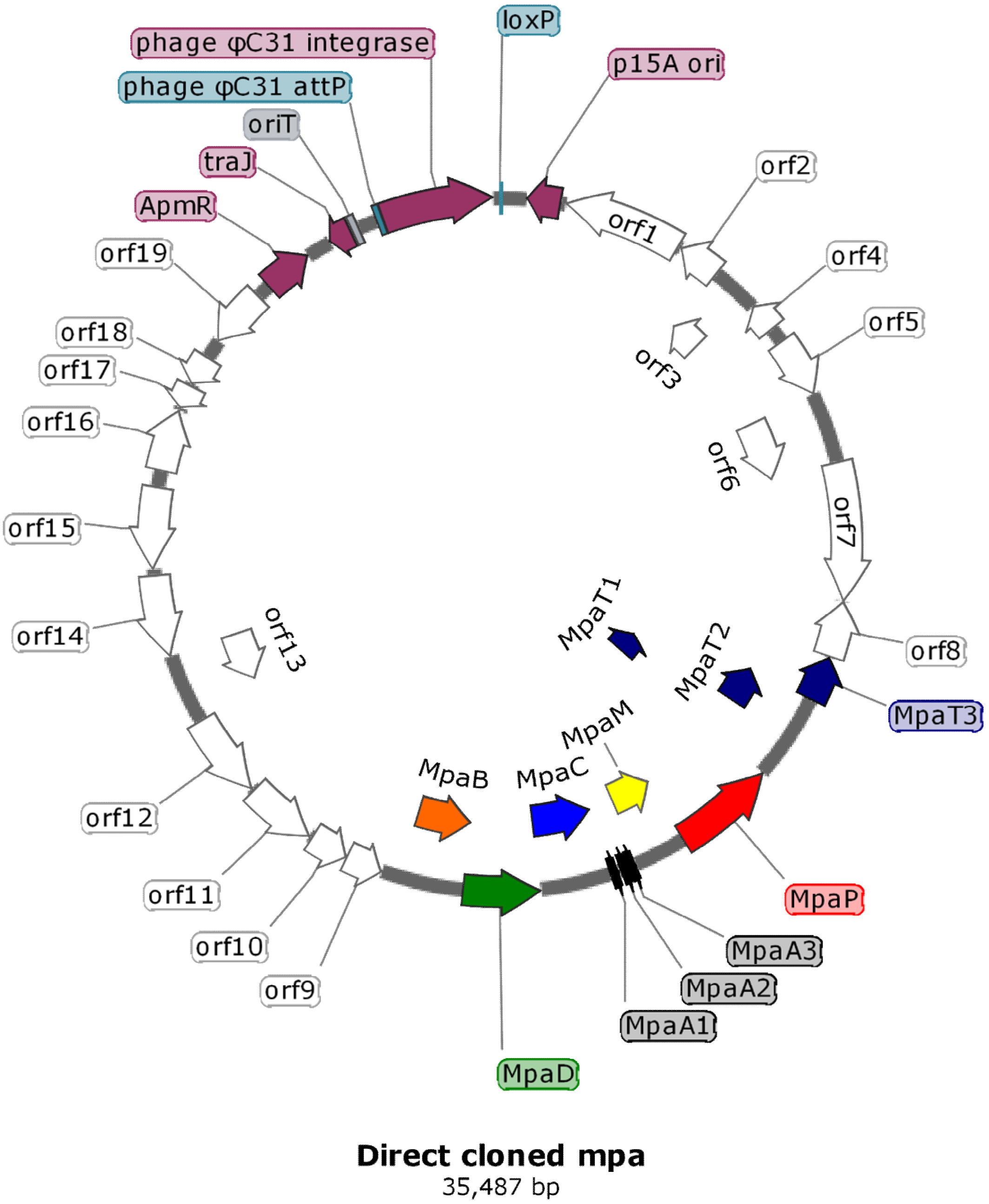

Supplementary Figure 36: The plasmid map of directly cloned *mpa* for heterologous expression in *S. albus* J1074. ApmR: apramycin resistance marker; traJ: conjugal transfer transcriptional regulator traJ; oriT: origin of transfer; loxP: Cre-Lox recombination site; p15A ori: p15A plasmid origin of replication. The plasmid map was drawn with SnapGene (GSL Biotech LLC). The plasmid sequence is available in **Source Data**.

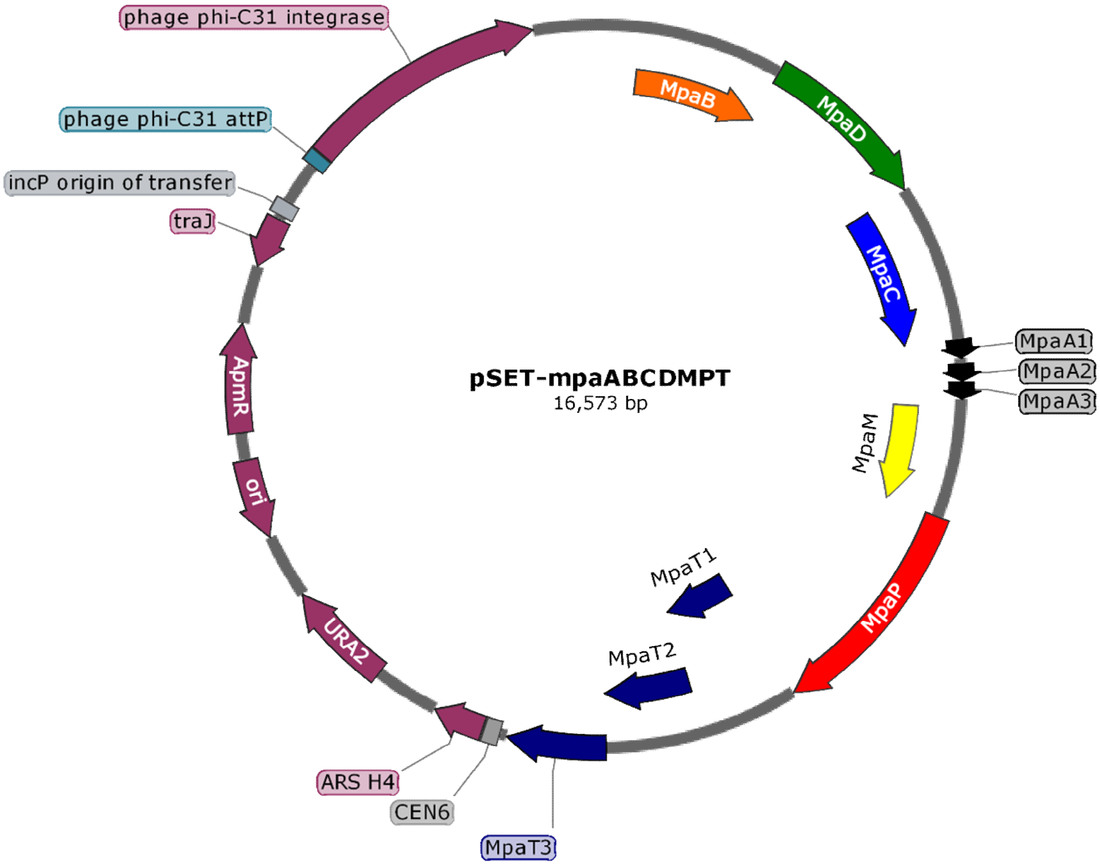

Supplementary Figure 37: Plasmid maps of gene omission for heterologous expression in *S. albus* J1074: pSET-mpaABCDMPT. ApmR: apramycin resistance marker; traJ: conjugal transfer transcriptional regulator traJ; oriT: origin of transfer; loxP: Cre-Lox recombination site; ori: p15A plasmid origin of replication; URA2: uracil auxotrophic marker; ARS H4: yeast artificial chromosome; CEN6: centromere in chromosome VI of *Saccharomyces cerevisiae*. The plasmid map was drawn with SnapGene (GSL Biotech LLC). The plasmid sequence is available in **Source Data**.

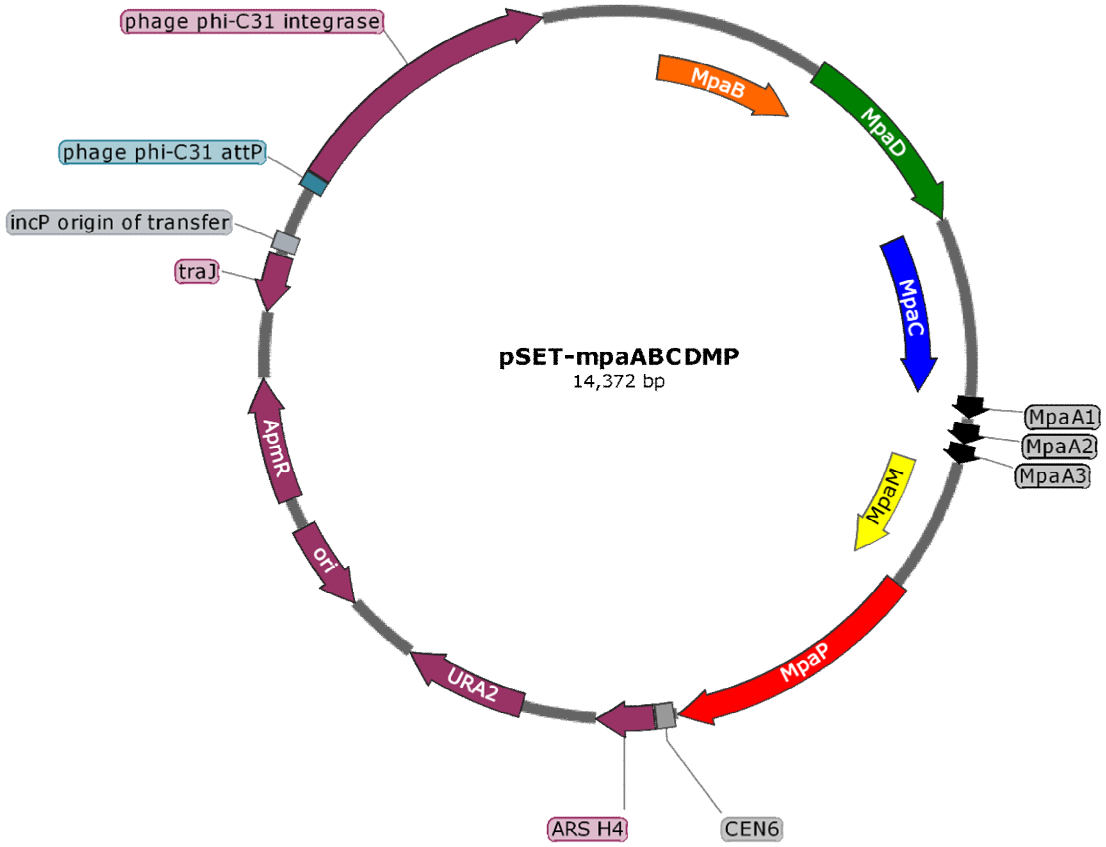

Supplementary Figure 38: Plasmid maps of gene omission for heterologous expression in *S. albus* J1074: pSET-mpaABCDMP. ApmR: apramycin resistance marker; traJ: conjugal transfer transcriptional regulator traJ; oriT: origin of transfer; loxP: Cre-Lox recombination site; ori: p15A plasmid origin of replication; URA2: uracil auxotrophic marker; ARS H4: yeast artificial chromosome; CEN6: centromere in chromosome VI of *Saccharomyces cerevisiae*. The plasmid map was drawn with SnapGene (GSL Biotech LLC). The plasmid sequence is available in **Source Data**.

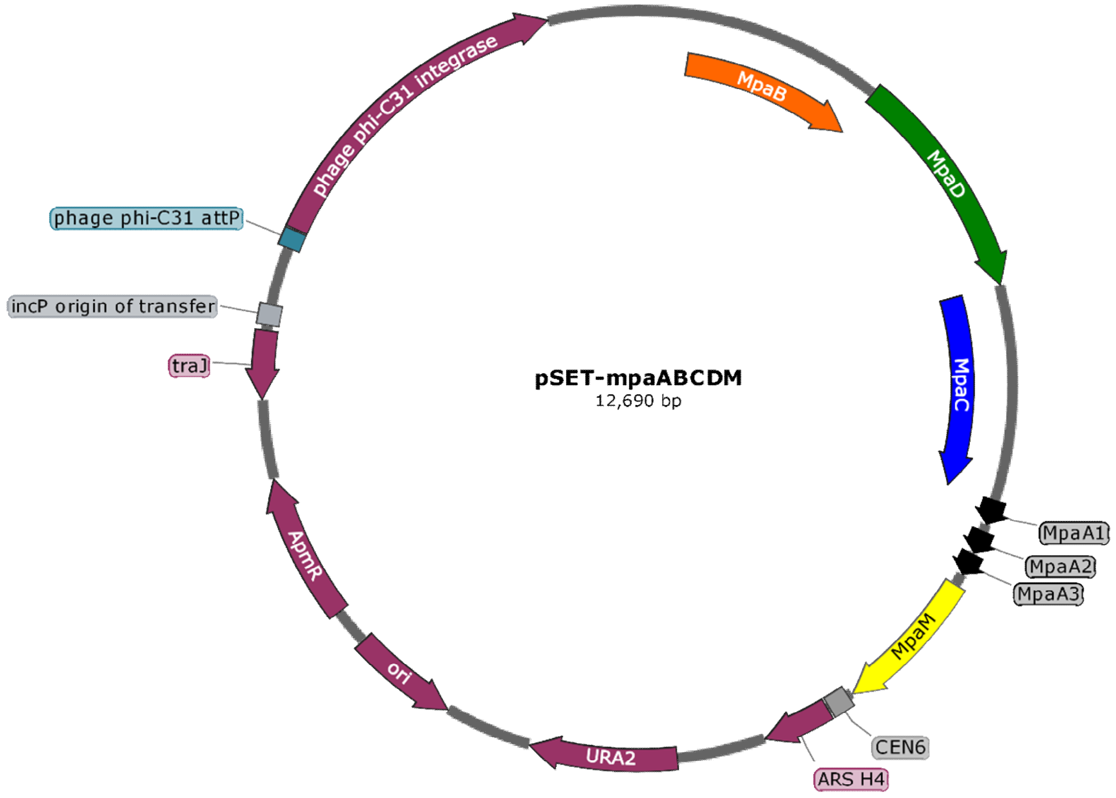

Supplementary Figure 39: Plasmid maps of gene omission for heterologous expression in *S. albus* J1074: pSET-mpaABCDM. ApmR: apramycin resistance marker; traJ: conjugal transfer transcriptional regulator traJ; oriT: origin of transfer; loxP: Cre-Lox recombination site; ori: p15A plasmid origin of replication; URA2: uracil auxotrophic marker; ARS H4: yeast artificial chromosome; CEN6: centromere in chromosome VI of *Saccharomyces cerevisiae*. The plasmid map was drawn with SnapGene (GSL Biotech LLC). The plasmid sequence is available in **Source Data**.

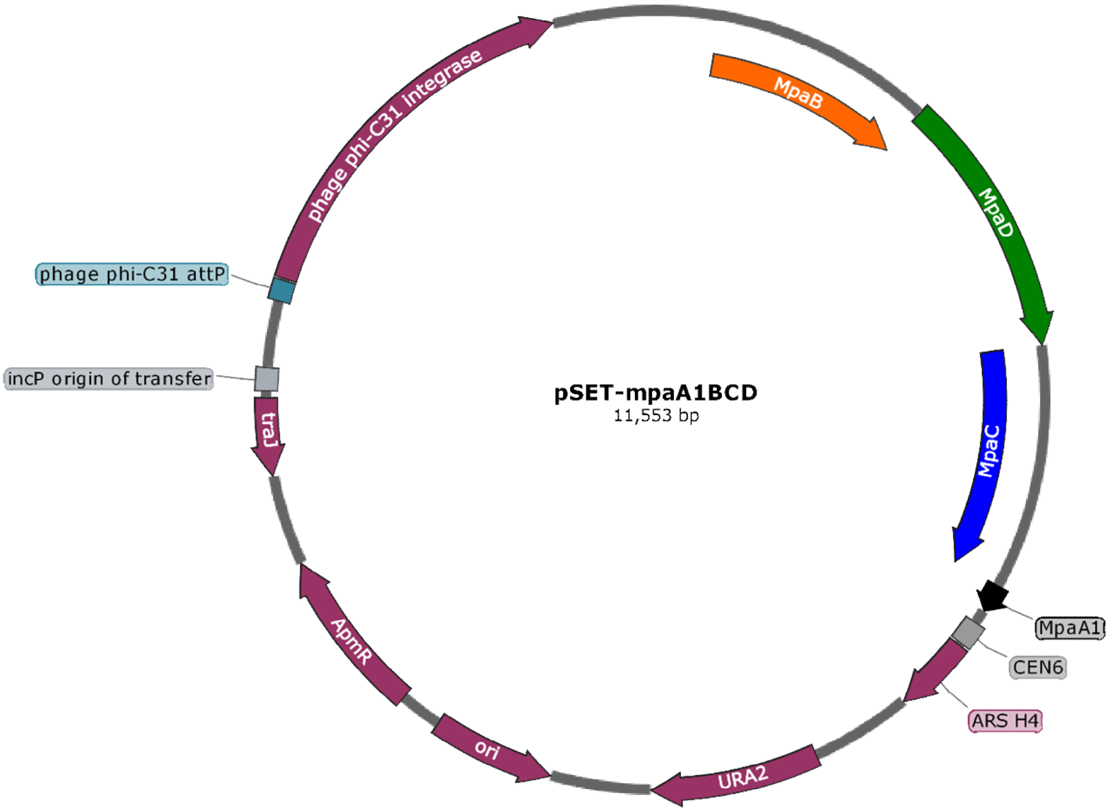

Supplementary Figure 40: Plasmid maps of gene omission for heterologous expression in *S. albus* J1074: pSET-mpaA1BCD. ApmR: apramycin resistance marker; traJ: conjugal transfer transcriptional regulator traJ; oriT: origin of transfer; loxP: Cre-Lox recombination site; ori: p15A plasmid origin of replication; URA2: uracil auxotrophic marker; ARS H4: yeast artificial chromosome; CEN6: centromere in chromosome VI of *Saccharomyces cerevisiae*. The plasmid map was drawn with SnapGene (GSL Biotech LLC). The plasmid sequence is available in **Source Data**.

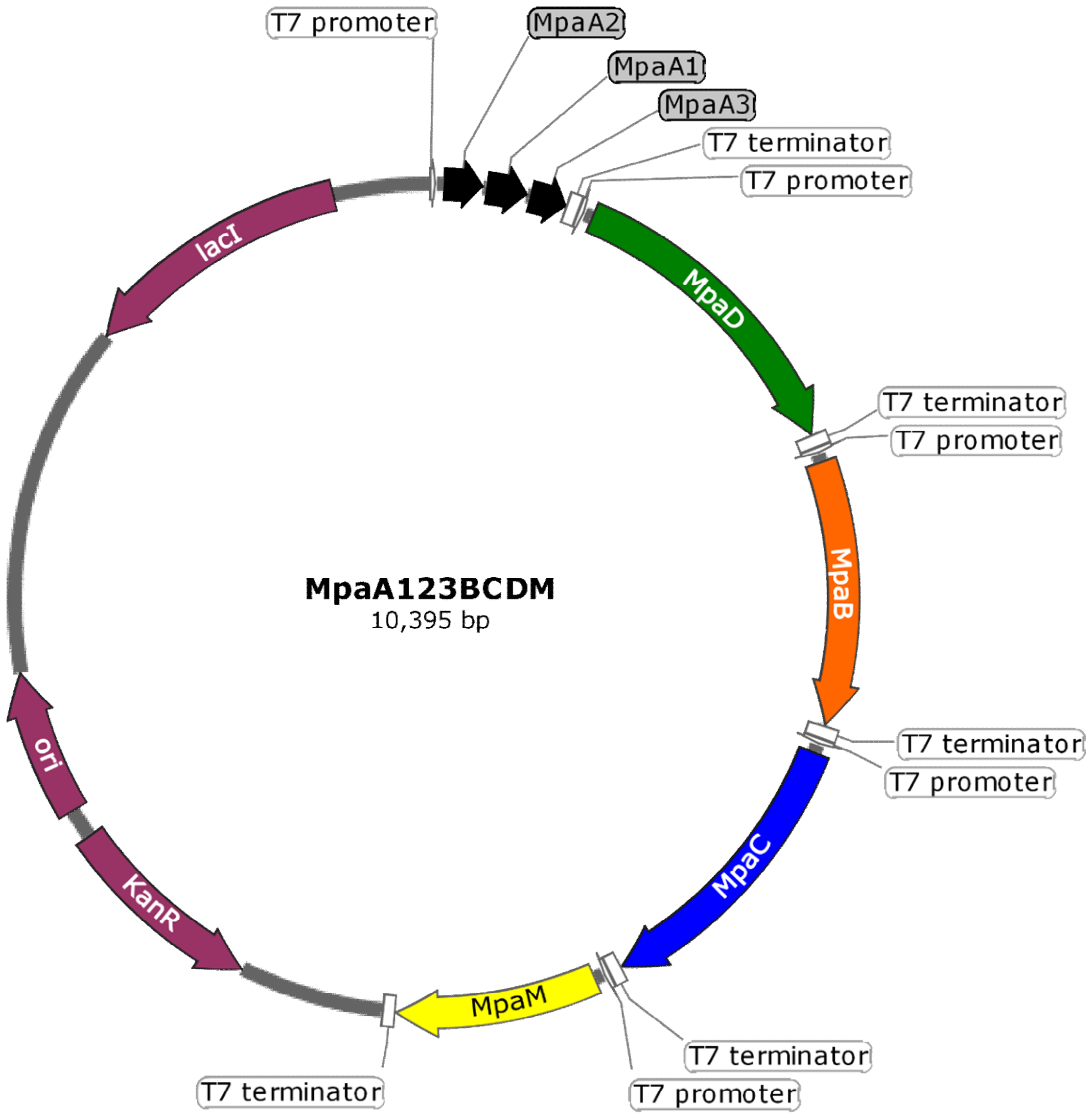

Supplementary Figure 41: Plasmid maps of codon-optimized and refactored genes for heterologous expression in *E. coli*: MpaA1A2A3BCDM. KanR: kanamycin resistance marker; ori: pBR322 plasmid origin of replication; lacI: lactose operon repressor lacI. The plasmid map was drawn with SnapGene (GSL Biotech LLC). The plasmid sequence is available in **Source Data**.

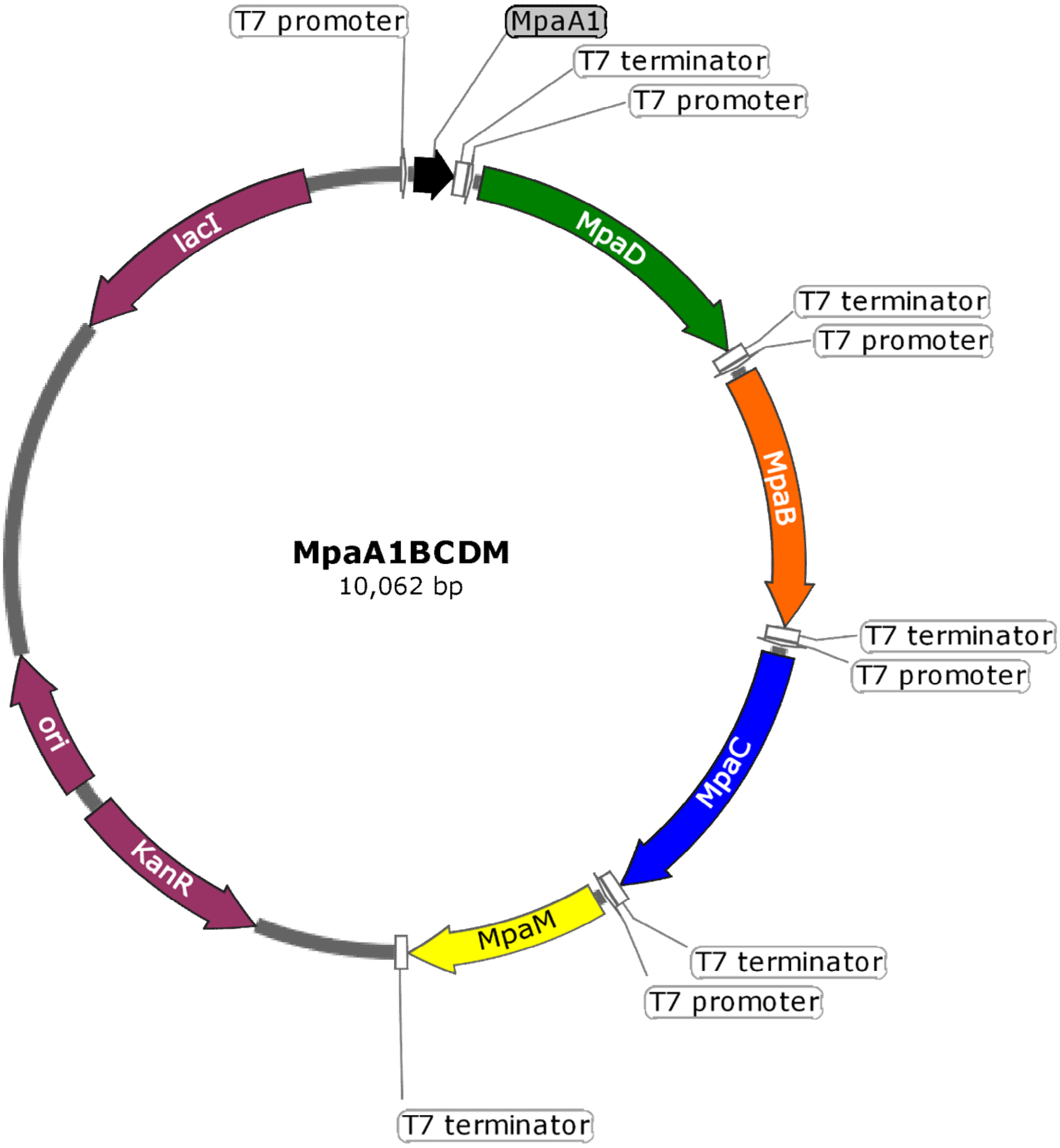

Supplementary Figure 42: Plasmid maps of codon-optimized and refactored genes for heterologous expression in *E. coli*: MpaABCDM. KanR: kanamycin resistance marker; ori: pBR322 plasmid origin of replication; lacI: lactose operon repressor lacI. The plasmid map was drawn with SnapGene (GSL Biotech LLC). The plasmid sequence is available in **Source Data**.

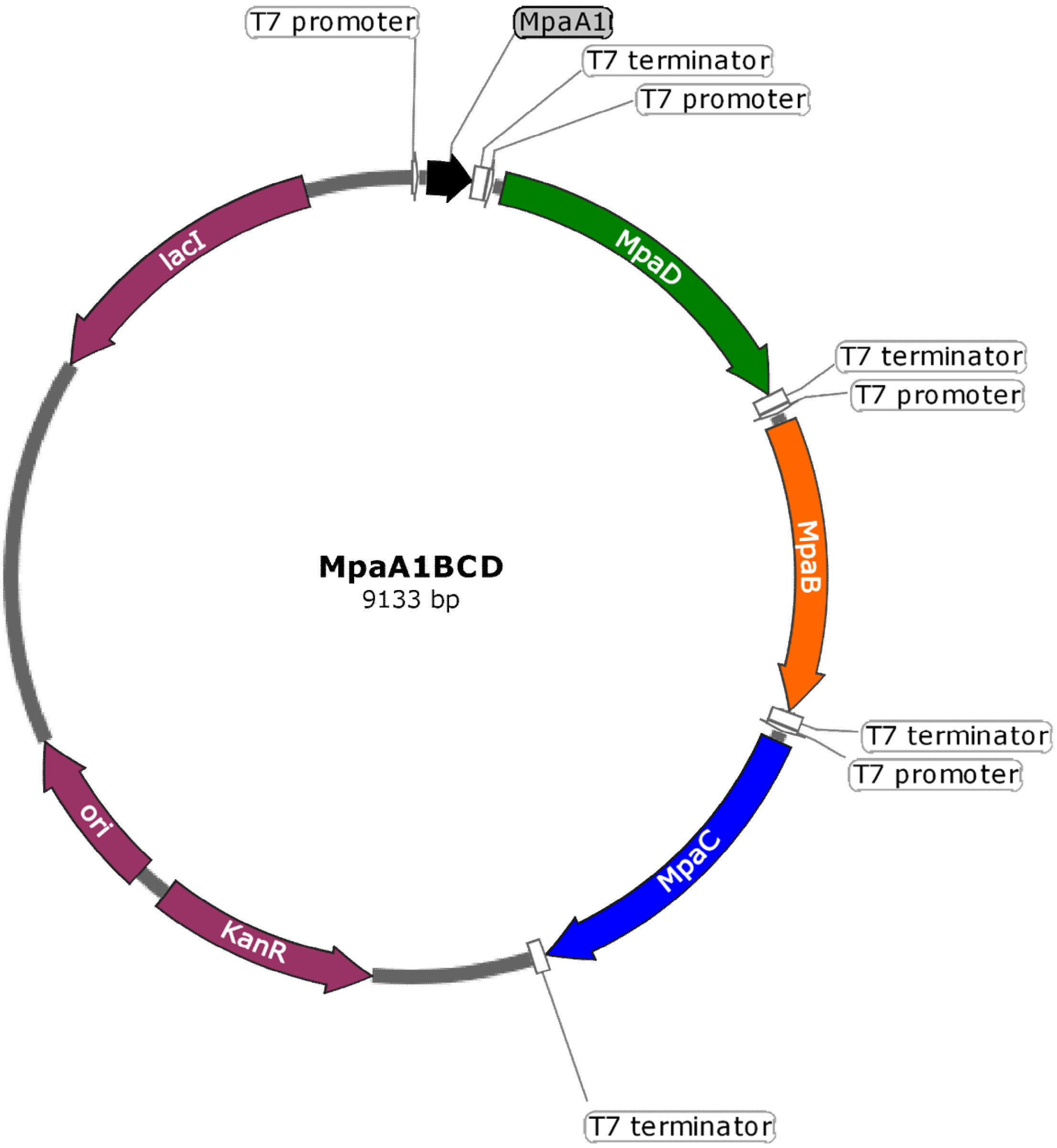

Supplementary Figure 43: Plasmid maps of codon-optimized and refactored genes for heterologous expression in *E. coli*: MpaABCD. KanR: kanamycin resistance marker; ori: pBR322 plasmid origin of replication; lacI: lactose operon repressor lacI. The plasmid map was drawn with SnapGene (GSL Biotech LLC). The plasmid sequence is available in **Source Data**.

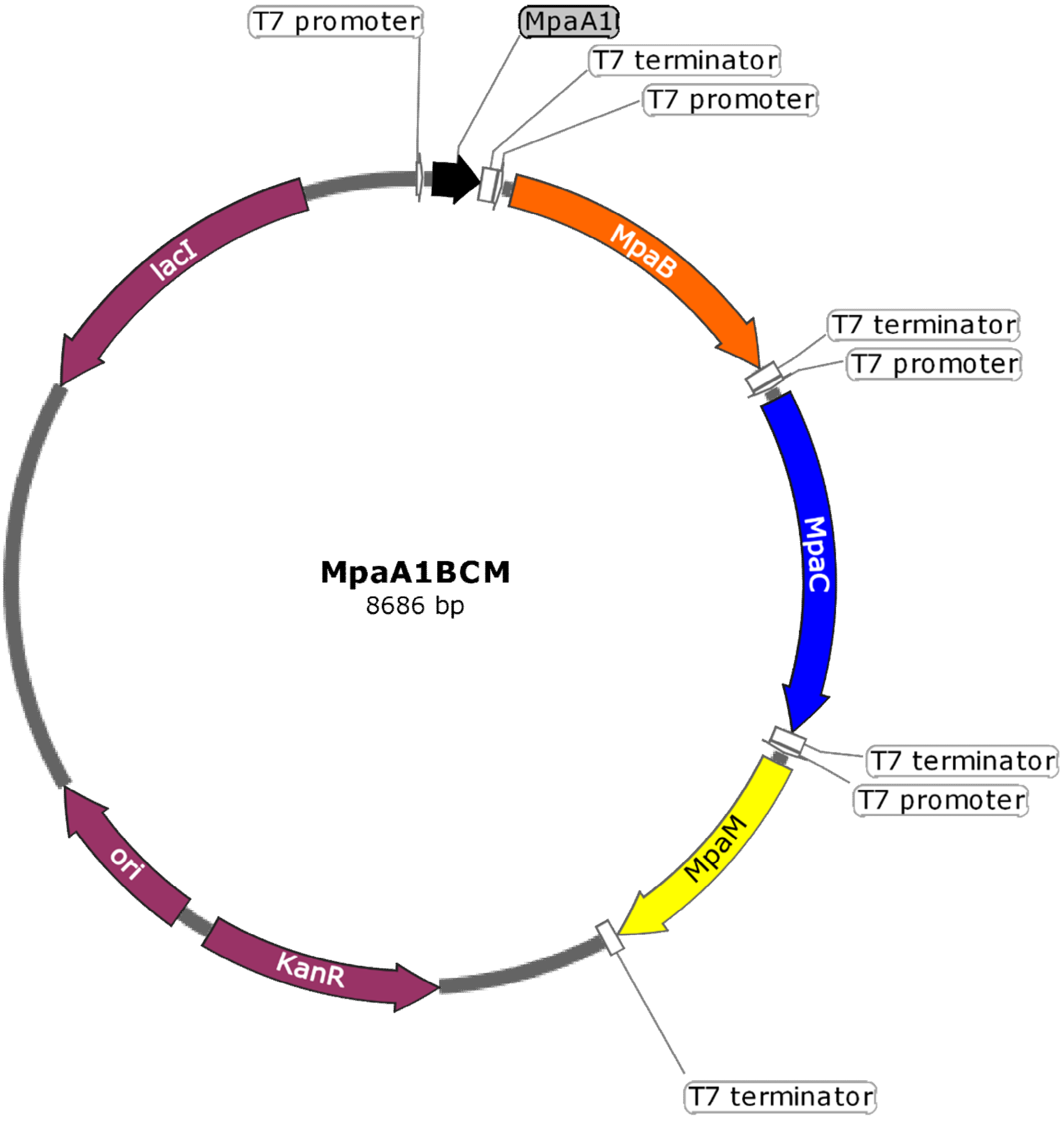

Supplementary Figure 44: Plasmid maps of codon-optimized and refactored genes for heterologous expression in *E. coli*: MpaABCM. KanR: kanamycin resistance marker; ori: pBR322 plasmid origin of replication; lacI: lactose operon repressor lacI. The plasmid map was drawn with SnapGene (GSL Biotech LLC). The plasmid sequence is available in **Source Data**.

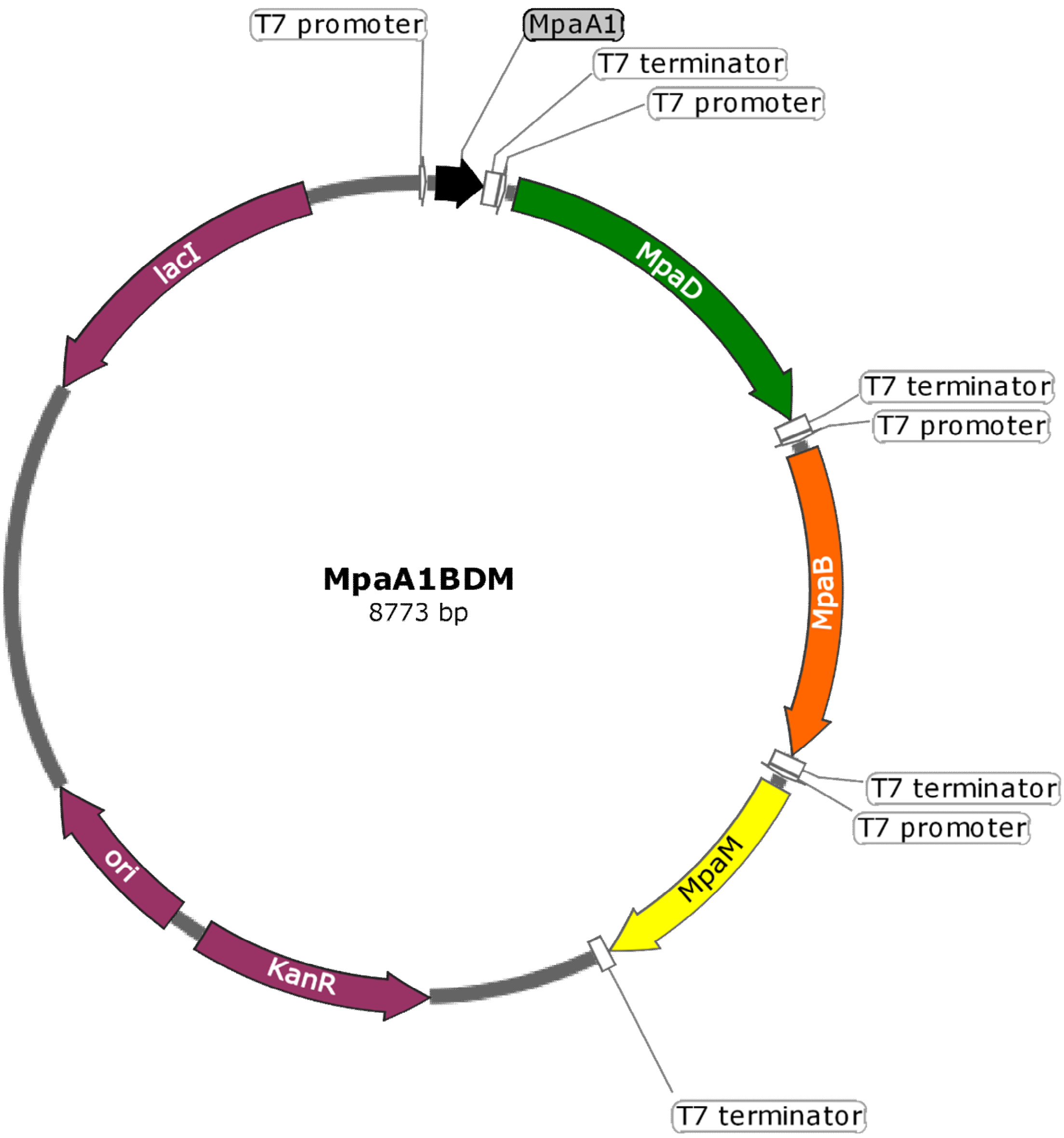

Supplementary Figure 45: Plasmid maps of codon-optimized and refactored genes for heterologous expression in *E. coli*: MpaABDM. KanR: kanamycin resistance marker; ori: pBR322 plasmid origin of replication; lacI: lactose operon repressor lacI. The plasmid map was drawn with SnapGene (GSL Biotech LLC). The plasmid sequence is available in **Source Data**.

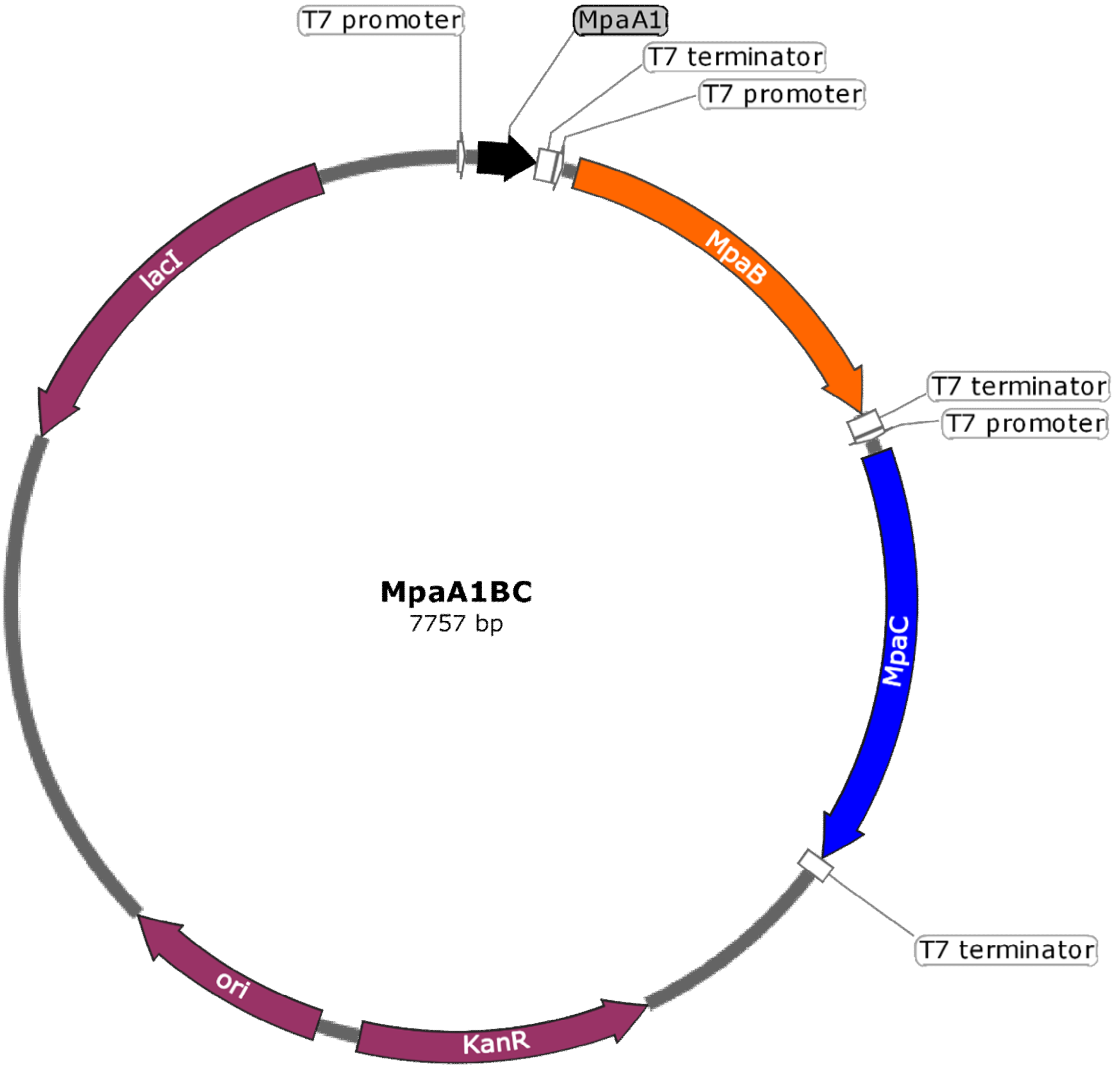

Supplementary Figure 46: Plasmid maps of codon-optimized and refactored genes for heterologous expression in *E. coli*: MpaABC. KanR: kanamycin resistance marker; ori: pBR322 plasmid origin of replication; lacI: lactose operon repressor lacI. The plasmid map was drawn with SnapGene (GSL Biotech LLC). The plasmid sequence is available in **Source Data**.

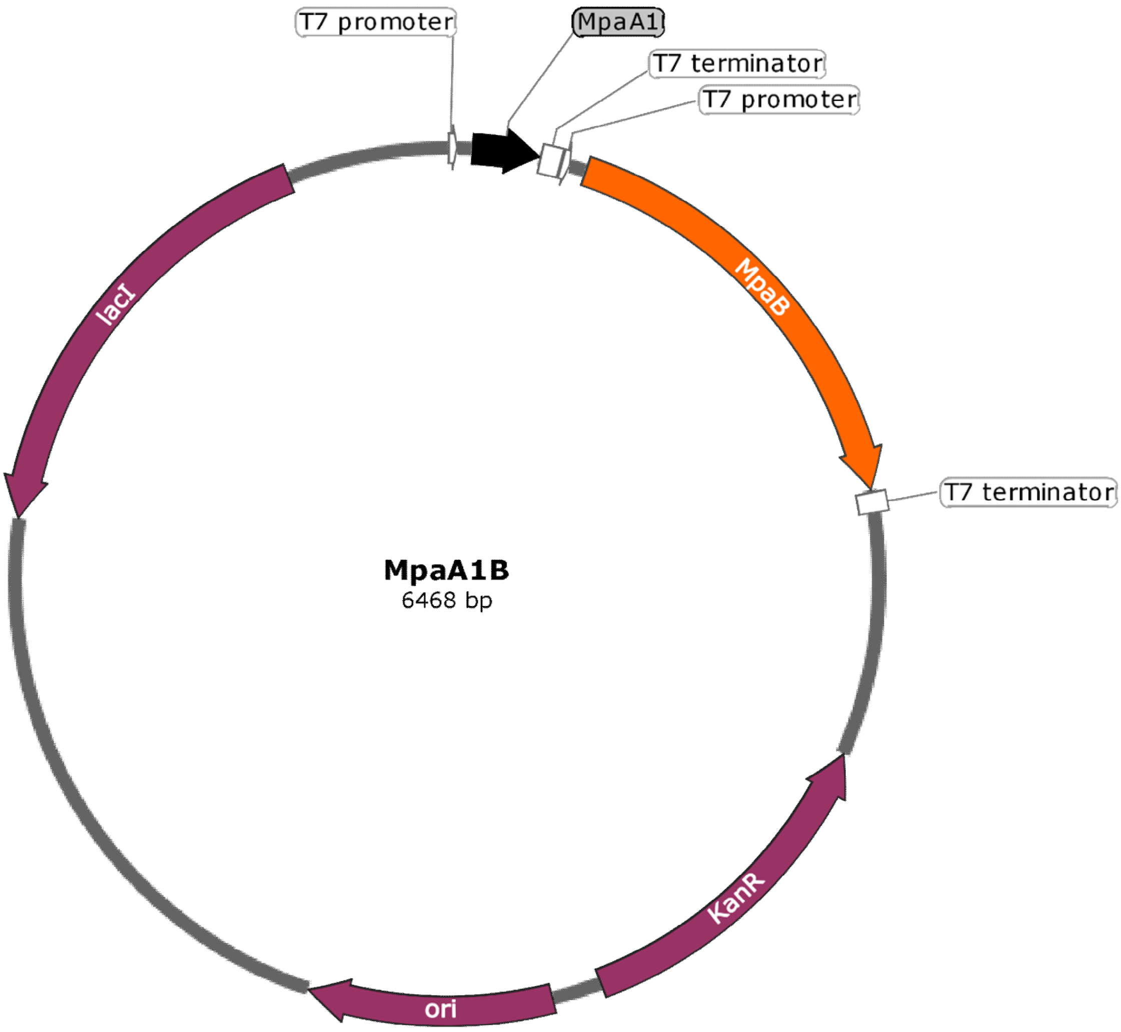

Supplementary Figure 47: Plasmid maps of codon-optimized and refactored genes for heterologous expression in *E. coli*: MpaAB. KanR: kanamycin resistance marker; ori: pBR322 plasmid origin of replication; lacI: lactose operon repressor lacI. The plasmid map was drawn with SnapGene (GSL Biotech LLC). The plasmid sequence is available in **Source Data**.

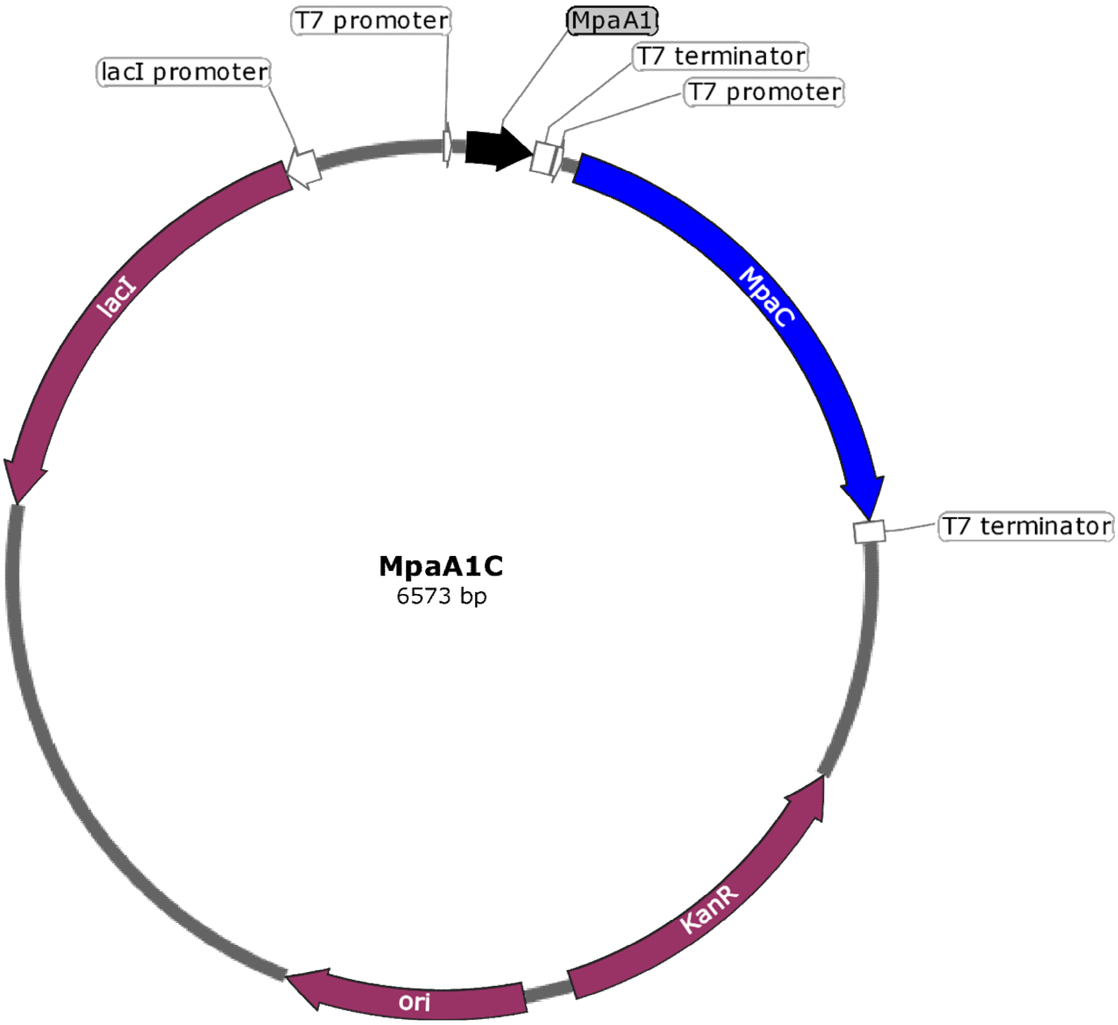

Supplementary Figure 48: Plasmid maps of codon-optimized and refactored genes for heterologous expression in *E. coli*: MpaAC. KanR: kanamycin resistance marker; ori: pBR322 plasmid origin of replication; lacI: lactose operon repressor lacI. The plasmid map was drawn with SnapGene (GSL Biotech LLC). The plasmid sequence is available in **Source Data**.

References

1. Blodgett, J.A. et al. Unusual transformations in the biosynthesis of the antibiotic phosphinothricin tripeptide. *Nat Chem Biol* **3**, 480-485 (2007).
2. Gregor, V.E. Tricyclic compound derivatives useful in the treatment of neoplastic diseases, inflammatory disorders and immunomodulatory disorders. *US 2014/0228350 A1*, 314 (2014).
3. Andres, J.M., Manzano, R. & Pedrosa, R. Novel bifunctional chiral urea and thiourea derivatives as organocatalysts: enantioselective nitro-Michael reaction of malonates and diketones. *Chem-Eur J* **14**, 5116-5119 (2008).
4. Tietz, J.I. et al. A new genome-mining tool redefines the lasso peptide biosynthetic landscape. *Nat Chem Biol* **13**, 470-478 (2017).
5. Mistry, J. et al. Pfam: The protein families database in 2021. *Nucleic Acids Res* **49**, D412-D419 (2021).
6. Haft, D.H. et al. TIGRFAMs: a protein family resource for the functional identification of proteins. *Nucleic Acids Res* **29**, 41-43 (2001).
7. Kloosterman, A.M., Shelton, K.E., van Wezel, G.P., Medema, M.H. & Mitchell, D.A. RRE-Finder: a genome-mining tool for class-independent RiPP discovery. *Msystems* **5**, e00267 (2020).
8. Kim, P.I. et al. Characterization and structure identification of an antimicrobial peptide, hominicin, produced by Staphylococcus hominis MBBL 2-9. *Biochem Bioph Res Co* **399**, 133-138 (2010).
9. Wheeler, T.J., Clements, J. & Finn, R.D. Skylign: a tool for creating informative, interactive logos representing sequence alignments and profile hidden Markov models. *Bmc Bioinformatics* **15**, 7 (2014).
10. Zimmermann, L. et al. A completely reimplemented MPI bioinformatics toolkit with a new HHpred server at its core. *J Mol Biol* **430**, 2237-2243 (2018).
11. Price, M.N., Dehal, P.S. & Arkin, A.P. FastTree 2--approximately maximum-likelihood trees for large alignments. *PLoS One* **5**, e9490 (2010).
12. Fountoulakis, M. & Lahm, H.W. Hydrolysis and amino acid composition analysis of proteins. *J Chromatogr A* **826**, 109-134 (1998).
13. Buchan, D.W.A. & Jones, D.T. The PSIPRED protein analysis workbench: 20 years on. *Nucleic Acids Res* **47**, W402-W407 (2019).
14. Jumper, J. et al. Highly accurate protein structure prediction with AlphaFold. *Nature* **596**, 583-589 (2021).
15. Hong, J.J. et al. How melittin inserts into cell membrane: Conformational changes, inter-peptide cooperation, and disturbance on the membrane. *Molecules* **24**, 1775 (2019).
16. Jurrus, E. et al. Improvements to the APBS biomolecular solvation software suite. *Protein Sci* **27**, 112-128 (2018).
17. Pymol: the PyMOL molecular graphics system, version 1.2r3pre, Schrödinger, LLC.
