# Supplementary material for: Genome mining unveils a class of ribosomal peptides with two amino termini
Source: Nat Commun. 2023 Mar 23;14:1624. doi: 10.1038/s41467-023-37287-1 (PMC10036551; doi:10.1038/s41467-023-37287-1)
Supplement: Supplementary file 3 — Description of Additional Supplementary Files [file 41467_2023_37287_MOESM3_ESM.pdf]

Description of Additional Supplementary Files

Supplementary Data 1: Bioinformatics results for the daptide class. Contains the gene co-occurrence data for all identified daptide BGCs, a list of identified daptide proteases, the list of commonly co-occurring enzyme families, and the list of identified daptide precursor peptides.

Supplementary Data 2: Phylogenetic data for daptide aminotransferase tree. Contains PhyloXML data required for recreation of the daptide aminotransferase phylogenetic tree.
